# Genome and transcriptome analysis of the Mesoamerican common bean and the role of gene duplications in establishing tissue and temporal specialization of genes

**DOI:** 10.1186/s13059-016-0883-6

**Published:** 2016-02-25

**Authors:** Anna Vlasova, Salvador Capella-Gutiérrez, Martha Rendón-Anaya, Miguel Hernández-Oñate, André E. Minoche, Ionas Erb, Francisco Câmara, Pablo Prieto-Barja, André Corvelo, Walter Sanseverino, Gastón Westergaard, Juliane C. Dohm, Georgios J. Pappas, Soledad Saburido-Alvarez, Darek Kedra, Irene Gonzalez, Luca Cozzuto, Jessica Gómez-Garrido, María A. Aguilar-Morón, Nuria Andreu, O. Mario Aguilar, Jordi Garcia-Mas, Maik Zehnsdorf, Martín P. Vázquez, Alfonso Delgado-Salinas, Luis Delaye, Ernesto Lowy, Alejandro Mentaberry, Rosana P. Vianello-Brondani, José Luís García, Tyler Alioto, Federico Sánchez, Heinz Himmelbauer, Marta Santalla, Cedric Notredame, Toni Gabaldón, Alfredo Herrera-Estrella, Roderic Guigó

**Affiliations:** Centre for Genomic Regulation (CRG), Barcelona Institute of Science and Technology (BIST), Dr. Aiguader 88, 08003 Barcelona, Spain; Universitat Pompeu Fabra (UPF), Dr. Aiguader 88, 08003 Barcelona, Spain; Yeast and Basidiomycete Research Group, CBS Fungal Biodiversity Centre, Uppsalalaan 8, 3584 LT Utrecht, The Netherlands; Laboratorio Nacional de Genómica para la Biodiversidad, Cinvestav-Irapuato, CP 36821 Irapuato, Guanajuato Mexico; Garvan Institute of Medical Research, 384 Victoria Street, Sydney, NSW 2010 Australia; New York Genome Center, 101 Avenue of the Americas, New York, NY 10013 USA; IRTA, Centre for Research in Agricultural Genomics (CRAG) CSIC-IRTA-UAB-UB, Campus UAB, 08193 Bellaterra, Barcelona, Catalonia Spain; Instituto de Agrobiotecnología Rosario (INDEAR), Rosario, Santa Fe 2000 Argentina; Department of Biotechnology, University of Natural Resources and Life Sciences (BOKU), Muthgasse 18, 1190 Vienna, Austria; Department of Cellular Biology, University of Brasilia, Biological Science Institute, Brasília, DF 70790-160 Brazil; Genomics Unit, Centre for Genomic Regulation (CRG), Barcelona Institute of Science and Technology (BIST), Dr. Aiguader 88, 08003 Barcelona, Catalonia Spain; CNAG-CRG, Centre for Genomic Regulation (CRG), Barcelona Institute of Science and Technology (BIST), Dr. Aiguader 88, 08003 Barcelona, Spain; Instituto de Biotecnología y Biología Molecular (IBBM), UNLP-CONICET, 1900 La Plata, Argentina; Departamento de Botánica, Instituto de Biología, Universidad Nacional Autónoma de México, 04510 Mexico City, Mexico; Departamento de Ingeniería Genética, Unidad Irapuato, Cinvestav, 36821 Irapuato, Guanajuato Mexico; European Molecular Biology Laboratory, European Bioinformatics Institute, Wellcome Trust Genome Campus, Hinxton, Cambridge CB10 1SD United Kingdom; Facultad de Ciencias Exactas y Naturales, Universidad de Buenos Aires (UBA), C1428EGA, Buenos Aires, Argentina; EMBRAPA Rice and Beans, Biotechnology Laboratory, Santo Antônio de Goiás, GO 75375-000 Brazil; Environmental Biology Department, Centro de Investigaciones Biológicas, (CSIC), 28040 Madrid, Spain; Depto. de Biología Molecular de Plantas, Instituto Biotecnología, Universidad Nacional Autónoma de México, 62210 Cuernavaca, Morelos Mexico; Mision Biológica de Galicia (MBG)-National Spanish Research Council (CSIC), 36080 Pontevedra, Spain; Institució Catalana de Recerca i Estudis Avançats (ICREA), Pg. Lluís Companys 23, 08010 Barcelona, Spain; IMIM (Hospital del Mar Medical Research Institute), 08003 Barcelona, Spain

**Keywords:** Common bean, BAT93, Gene duplication, Tissue expression, Transcriptome, lncRNAs

## Abstract

**Background:**

Legumes are the third largest family of angiosperms and the second most important crop class. Legume genomes have been shaped by extensive large-scale gene duplications, including an approximately 58 million year old whole genome duplication shared by most crop legumes.

**Results:**

We report the genome and the transcription atlas of coding and non-coding genes of a Mesoamerican genotype of common bean (*Phaseolus vulgaris* L., BAT93). Using a comprehensive phylogenomics analysis, we assessed the past and recent evolution of common bean, and traced the diversification of patterns of gene expression following duplication. We find that successive rounds of gene duplications in legumes have shaped tissue and developmental expression, leading to increased levels of specialization in larger gene families. We also find that many long non-coding RNAs are preferentially expressed in germ-line-related tissues (pods and seeds), suggesting that they play a significant role in fruit development. Our results also suggest that most bean-specific gene family expansions, including resistance gene clusters, predate the split of the Mesoamerican and Andean gene pools.

**Conclusions:**

The genome and transcriptome data herein generated for a Mesoamerican genotype represent a counterpart to the genomic resources already available for the Andean gene pool. Altogether, this information will allow the genetic dissection of the characters involved in the domestication and adaptation of the crop, and their further implementation in breeding strategies for this important crop.

**Electronic supplementary material:**

The online version of this article (doi:10.1186/s13059-016-0883-6) contains supplementary material, which is available to authorized users.

## Background

Legumes are the third largest family of angiosperms and include many populous species. The majority of legumes contain symbiotic bacteria within nodules in their roots that mediate nitrogen fixation and provide an advantage towards competing plants. Legume seeds are rich in protein content and thus many species have been used for human or animal consumption over the years. Legumes as a whole constitute the second largest class of crops, including peas, soybeans, peanuts, and beans. Common bean (*Phaseolus vulgaris* L.), a major source of protein that complements carbohydrate-rich rice, maize, and cassava, is fundamental for the nutrition of more than 500 million people in developing countries [1]. Even though the origin of *P. vulgaris* as a species was debated for years [2, 3], recent studies suggest it originated in Mesoamerica [4] and then migrated to the Andean region in South America, giving rise to two wild populations or gene pools. Using a limited number of loci, the splitting of both gene pools was dated 111,000 years ago [5]; however, demographic inferences using polymorphic sites distributed all along the genome resulted in a tight interval of 146,000–184,000 years ago [6]. Both analyses indicate that common bean dispersal along the Americas occurred prior to human migrations. Over 100,000 years after the split of the Mesoamerican and Andean gene pools (~8200–8500 years ago [7]), at least two independent domestication events started, one per population, slowly shaping what we know today as cultivated populations and landraces [8, 9]. The age of the *Phaseolus* stem clade (~6–8 million years ago [10]), the estimated age of diversification of the *Phaseolus* extant species clades (~2 million years ago [10]), the elapsed time after the geographic isolation of the two gene pools, the continuous domestication processes accompanied by population bottlenecks [11], and the evidence of genetic flow between wild and domesticated sub-populations [12–14] open several questions regarding common bean genome shaping (gene duplications, gene family expansions, and the emergence of polymorphisms) that ultimately led to the phenotypic traits we observe in modern cultivars. The availability of the genomic sequences of these two gene pools would certainly contribute to the understanding of this complex evolutionary history. In 2014, the first genome of an Andean *P. vulgaris* landrace was published [6, 15]. Here we determined the complete genome sequence of the *P. vulgaris* Mesoamerican breeding line BAT93, accompanied by a detailed transcriptomic atlas of the different bean organs and tissues through the entire development of the plant. Finally, we reconstructed the evolutionary history of each common bean gene, across the two sequenced varieties and other sequenced plant species.

Our analyses allowed the identification of a set of legume- and *P. vulgaris*-specific coding and non-coding genes, including a core set of conserved plant long non-coding RNAs (lncRNAs). Through the analysis of the patterns of gene expression across organs and developmental stages, we identified organ- and stage-specific genes. We found that, while organ-specific protein coding genes are overwhelmingly expressed in the roots, organ-specific lncRNAs tend to be specific for fruits. Consistently, our analysis of co-expression networks also reveals an important role for a few novel lncRNAs in fruit development.

By overlaying evolutionary information on the transcriptional landscape of BAT93, we found that gene duplication has shaped tissue expression in legumes, with the level of tissue specialization increasing with both time of divergence and number of retained duplicates. Ancient genes without paralogs tend to have broad expression and form the most densely connected hubs in the co-expression network, whereas recently emerged genes and those that belong to large, multi-gene families tend to be expressed narrowly, have fewer co-expressed partners, and are associated with specialized functions in specific tissues. Given the fact that most bean-specific gene family expansions herein detected predate the split of the Mesoamerican and Andean gene pools, we suggest they were key events that facilitated broad distribution of common bean in America, making this species prone to human discovery and further domestication. Altogether, the genomic, transcriptomic and evolutionary features derived from our study constitute a major resource to investigate the common and specific traces of the *P. vulgaris* gene pools, and to understand how members of the same species have adapted to different environmental conditions such as those present in the Andean and Mesoamerican regions.

## Results

### Genome sequencing and assembly

**Fig. 1:**
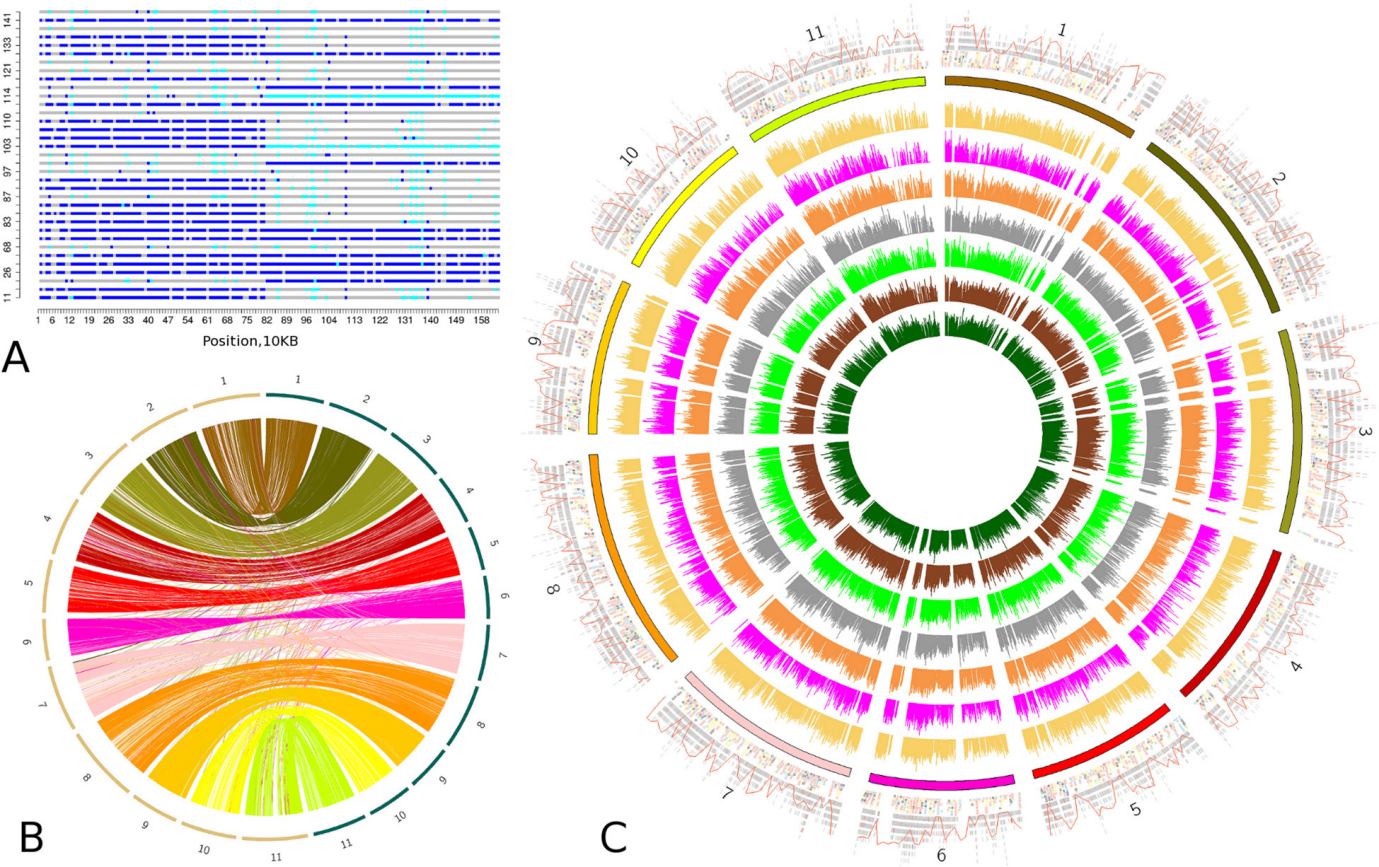
BAT93 assembly overview. **a** An example of a genotype-by-sequencing (GBS) profile for the scaffold scaffold00017. The defined mis-assembly point is at the center. Colors indicate different variants between the GBS samples and the reference genome: *blue*, homozygous variant; *light blue*, heterozygous variant; *grey*, absence of any variant. Colors correspond to the linkage groups. **b** Synteny-like comparison of one-to-one ortologs between BAT93 (*green*) and G19833 (*brown*) linkage groups. Colors correspond to the linkage groups, as in (**c**). **c** Circos plot representing the gene content and transcriptome maps of the linkage groups of *P. vulgaris*. The outer ring represents the localization of genes across bean linkage groups. *Grey regions* are meant to contain genes and *white regions* depleted from annotated genes. The *red line* shows the repeat coverage across the linkage groups. Below, *squares* of different colors represent different types of genes: *red*, smallRNAs; *blue*, lncRNAs; *yellow*, legume-specific; *black*, resistance. The inner rings below the horizontal bar delineating the linkage groups represent RNA-Seq coverage for the different organs: axial meristem, flower, pod, seed, leaf, root and stem

We assembled the *P. vulgaris* Mesoamerican common bean BAT93 genome using a hybrid sequencing strategy involving 454 single reads and 8, 10, and 20 kb mate pair libraries; 3 and 5 kb SOLiD mate pair libraries; and Sanger bacterial artificial chromosome (BAC)-end and genomic read pairs (Additional file 1: Table S1). Data free of redundancies were used as input for a Newbler assembly, and Illumina reads (45× coverage) were used to correct homopolymer errors and close or reduce gaps within scaffolds (Additional file 1: Tables S2 and S3). Illumina genotyping-by-sequencing (GBS) [16] data from a set of 60 F5 lines of a BAT93 × Jalo EEP558 advanced intercross (6.7× coverage per line on average; Additional file 2: Dataset S1), together with 827 public marker sequences, were used for assembly correction and scaffold anchoring. Up to 900,000 variants distinguishing Jalo from BAT93 were scored on scaffolds exceeding 20 kb. Discontinuous genotype profiles observed in 48 cases were manually corrected by breaking scaffolds at the mis-assembly points (Fig. 1a; Additional file 1: Figure S1). Markers were aligned to the assembly and GBS profiles of these scaffolds were used as seeds to place other scaffolds with this or similar profiles onto chromosomes, followed by genetic map calculation. The final BAT93 genome sequence encompassed 549.6 Mb (Table 1), close to previous size estimates [17, 18], with 81 % of the assembly anchored to eleven linkage groups (Fig. 1b; Additional file 1: Tables S4 and S5). The assembly included 97 % of the conserved core eukaryotic genes [19], thus reflecting its completeness.

**Table 1:** Summary of *P. vulgaris* cv. BAT93 genome assembly

	Whole genome	Scaffolds only
Assembly		
Total length	549,604,264	494,957,111
Number of scaffolds/contigs	68,379	9,047
N50(size/number)	433,759 / 324	526,483 / 267
N90(size/number)	2,023 / 8,894	35,958 / 1,484
Range (min-max)	500-3,177,954	2,000-3,177,954
% of Ns	34.96 %	36.99 %
G + C content	38.43 %	36.64 %
Annotation		
Number of protein coding (PC) genes	30,491	29,569
Number of PC transcripts	66,634	65,685
Number of small RNAs	2,529	2,271
Number of long non-coding genes	1,033	870
G + C content exonic (for PC genes)	47.57 %	47.70 %
Number of functionally annotated transcripts	62,713 (94.12 %)	62,594 (95.2 %)

The "*Whole genome*" column corresponds to the entire set of scaffolds and unplaced contigs, while the "*Scaffolds only*" column corresponds only to the set of scaffolds. Complete annotation statistic are provided in Additional file 1: Table S15

### Genome annotation

We identified transposable elements by combining *de novo* and homology-based approaches, finding 35 % of the *P. vulgaris* BAT93 genome assembly to be covered by repeats, mostly long terminal repeats (LTRs; Additional file 1: Table S6). To aid in gene prediction and to obtain a global view of the transcriptome during development, we sequenced with Illumina 61 RNA samples from 34 different organs and/or developmental stages from healthy plants (Additional file 1: Tables S7 and S8). In addition, two normalized libraries derived from 162 RNA samples from plants grown under optimal and stress conditions were used for 454 pyrosequencing (Additional file 1: Tables S9–S12). Illumina and 454 RNA-Seq reads, as well as public expressed sequence tags (EST) and cDNA sequences, were combined with *ab initio* predictions to produce an initial gene set (Additional file 1: Tables S13 and S14). This was filtered to remove genes lacking both similarity to other plant proteins and any evidence of expression, resulting in 30,491 protein coding genes (PCGs), whose 66,634 transcripts encode 53,904 unique proteins (Additional file 1: Table S15). Using protein signatures and phylogeny-based transference of functional annotations we were able to associate functions with 94 % of the bean transcripts, with 76 % of them specifically associated with Gene Ontology (GO) terms (Additional file 1: Tables S16 and S17, Figures S2 and S3).

We compared our PCG model predictions with that of the Andean *P. vulgaris* G19883 genome [6] using a combination of synteny and phylogeny-based orthology assignment between both genomes (details in "Materials and methods"; Additional file 1: Table S18). Out of the 25,991 BAT93 PCGs that could be placed in linkage groups, 20,617 were uniquely mapped to 20,618 PCGs in the Andean genome (Fig. 1b). When considering both placed and unplaced PCGs, 21,600 BAT93 PCGs were mapped to 21,604 PCGs in the G19833 genome. We then aligned the protein coding sequences of these equivalent genes and found that 1186 PCG pairs have sequence identity lower than 95 % when gaps are not considered (Additional file 1: Table S19). These divergent gene pairs are mainly enriched in defense response and terpene synthase activity (Additional file 1: Table S20). Terpene has been described before as an indirect defense mechanism in legumes [20].

Then, we attempted to specifically characterize resistance genes, as the Mesoamerican BAT93 line has been described as less susceptible to diseases such as bean common mosaic virus rust, angular leaf spot, anthracnose or common bacterial blight compared with its Andean counterpart [21, 22]. We identified 852 putative resistance genes in the BAT93 genome (Additional file 1: Table S21), which include 234 belonging to the cytoplasmic NBS-LRR class. In comparison, G19833 had been predicted to contain 376 cytoplasmic NBS-LRR class genes, of which 316 could be mapped to 220 BAT93 genes. Out of the NBS-LRR class, we were able to place 211 and 182 genes from BAT93 and G19833, respectively, into the Mesoamerican linkage groups (Additional file 1: Figure S4). The placement allowed us to recapitulate the gene clusters observed by Schmutz et al. [6]. However, we were unable to find resistance-gene clusters that were specific to either of the two varieties. These results indicate that the genomic clustering of resistance genes predates the split of both gene pools and suggest that the differences in pathogen susceptibility might be due to polymorphisms in these loci, rather than a gene presence–absence effect. Additionally, when BAT93 Illumina reads were mapped to the G19833 assembly we identified 10,193 regions of 1 kb or longer with zero coverage containing a total of 314 PCGs. These genes are likely lost specifically in BAT93. Although no functional enrichment was detected, 17 PCGs are annotated as involved in defense resistance (5.4 %, a proportion almost twice as large as that in the whole BAT93 bean genome, 2.8 %).

**Fig. 2:**
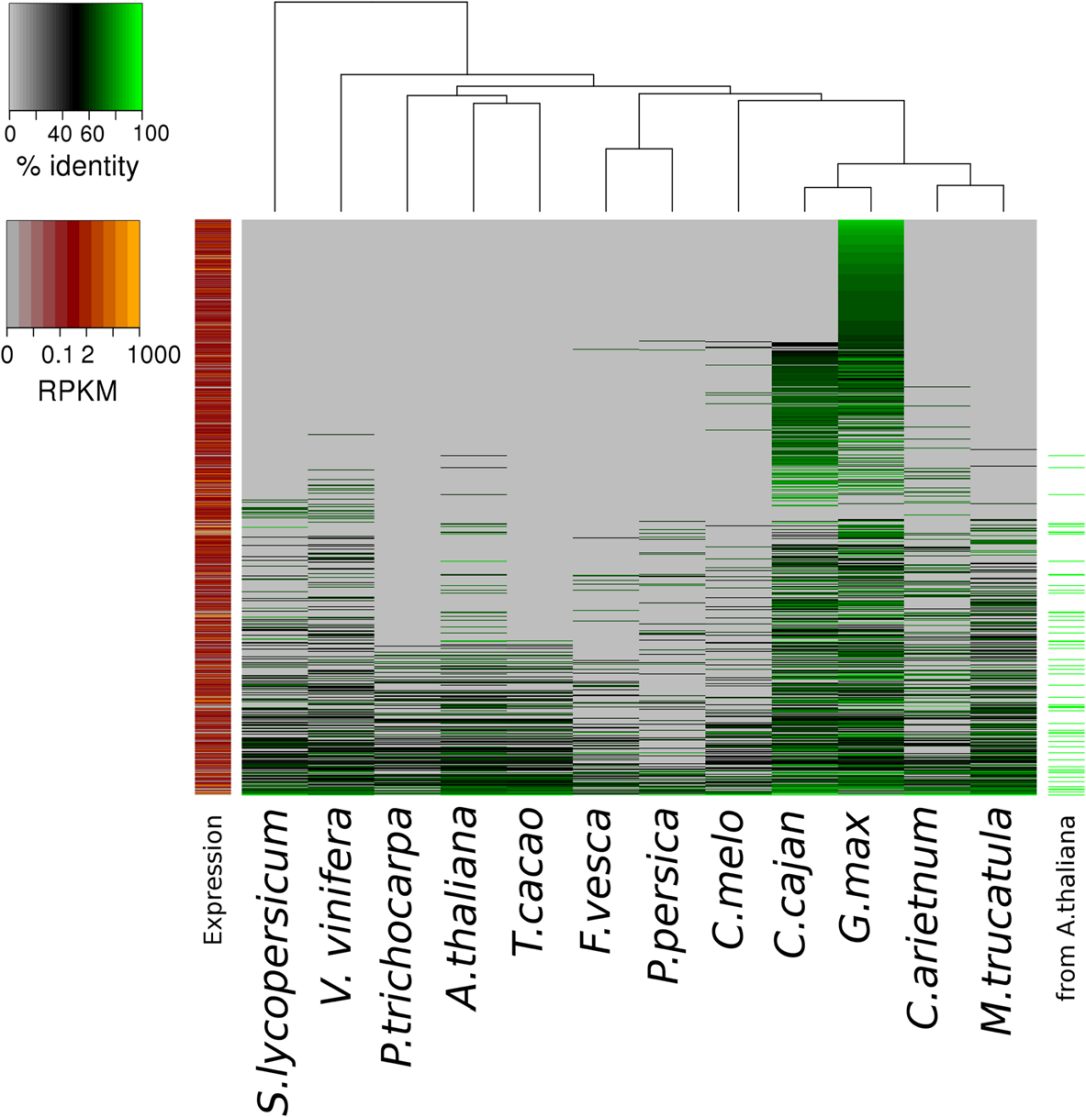
Conservation and expression pattern of lncRNAs in *P. vulgaris*. Phylogenomics profiling of lncRNA transcripts in 12 plant species. Shown are 762 bean transcripts (belonging to 507 genes) conserved in at least one other plant species. Percentage of sequence identity with bean is shown as a heat map, where *green* denotes high similarity and *grey* missing transcripts. The leftmost column indicates average expression levels in bean, the rightmost column marks 56 transcripts inferred from *A. thaliana* homologues

In addition to PCGs, we identified and annotated small RNA (sRNA) and long non-coding RNA (lncRNA) sequences. In silico homology modeling based on sRNA sequencing led to the identification of 2529 sRNAs belonging to plant known families (Additional file 1: Table S22, Figure S5). lncRNAs were identified by combining *Arabidopsis thaliana* homology-based predictions and computationally predicted transcript models based on RNA-Seq data. Once filtered from single exon models, putative open reading frames (ORFs), and transcripts mapped within 1 kb of annotated PCGs [23], we obtained 1033 intergenic lncRNAs (38 inferred from *A. thaliana*), coding for 1858 transcripts (Additional file 1: Table S23). We found 94 % of the lncRNAs in the Mesoamerican genome were also present in the Andean genome*.* Homology profiling against 12 other complete plant genomes revealed 526 bean-specific lncRNA genes and only five lncRNAs conserved in all 12 plant genomes (Fig. 2; Additional file 1).

### The bean phylome

**Fig. 3:**
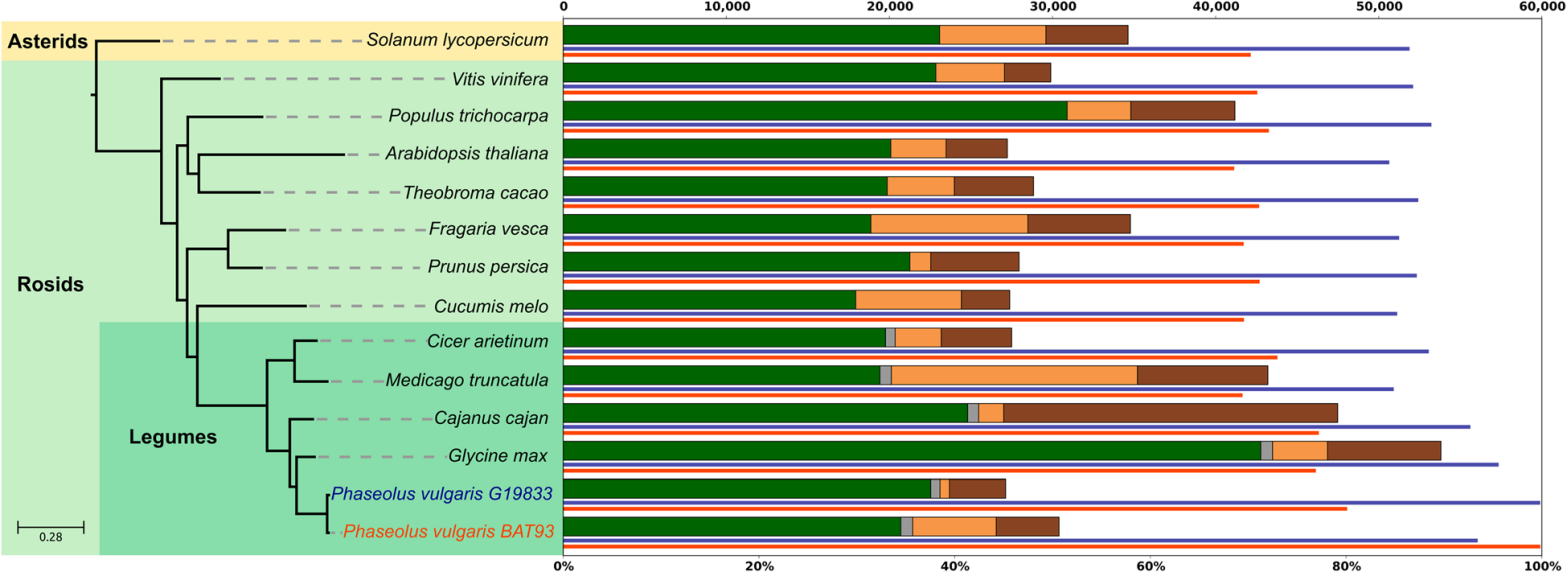
Phylogenomics analysis. The species phylogeny is based on maximum-likelihood analyses of a concatenated alignment of 172 widespread, single-copy orthologous genes. The two different *P. vulgari*s accessions used in this phylogeny are colored differently. *Bars* represent the total number of genes for each species (scale on the top) and are divided to indicate different types of phylogenetic profiles: *green*, widespread proteins which are found in at least 12 of the 14 species; *grey*, widespread but legume-specific proteins which are found in at least four of the six legumes species; *light-orange*, genes without a clear phylogenetic profile; *brown*, species-specific genes with no (detectable) homologs in other species. The *thin blue line* under each bar represents the percentage of *P. vulgaris* G19833 genes which have homologs in a given species. Conversely, the *thin orange line* represents the percentage of *P. vulgaris* BAT93 genes which have homologs in a given species

To gain insight into *P. vulgaris* genome evolution*,* we reconstructed its phylome, i.e., the complete collection of evolutionary histories of bean genes, using PCG sets derived from either BAT93, G19833 or both genomes. We obtained 27,986 trees for the BAT93 phylome (available through PhylomeDB [24, 25]), and scanned them to detect and date gene duplication events, delineate orthology and paralogy relationships [26, 27], and annotate functions (Additional file 1: Tables S24–S27). We reconstructed a species phylogeny using two complementary approaches: (i) the analysis of 172 sets of widespread groups of one-to-one orthologs, and (ii) a super-tree reconstruction using 82,365 single-gene trees from the three phylomes above. Both approaches yielded an identical topology (Fig. 3), which provides an evolutionary framework for downstream comparative genomics analyses. From this phylogeny we defined four evolutionary periods as the lineages preceding the divergence of *Phaseolus*: basal to *Phaseolus*; basal to legumes; basal to rosids; and basal to the split of rosids and asterids. We then assigned the duplications inferred from gene trees to each of these periods (Additional file 1: Tables S28 and S29). The resulting pattern of duplication densities is consistent with the proposed wave of whole genome duplication events at the split of rosids and asterids [28], and at the base of legumes [29, 30]. However, in contrast to what has been observed in soybean [31], we found no footprints that a recent whole genome duplication occurred in any of the two sequenced *P. vulgaris* lineages. We assessed functional enrichment among genes restricted to specific clades or specifically duplicated in the lineages described above. The largest gene family expansion specific to BAT93 corresponded to putative cellular receptors with extracellular domains (Additional file 1: Figure S6–S8; Additional file 2: Dataset S2). We found two additional BAT93-specific expansions that were functionally enriched in seed development and the ubiquitin pathway. We found several gene family expansions common to BAT93 and G19833 in which the gene tree topologies suggested that duplications preceded the divergence of the two lineages. These duplications are enriched in genes involved in defense response and response to stress (Additional file 2: Dataset S3). Genes widespread in legumes but absent from other species were enriched for functions related to symbiosis with soil microorganisms and pathogen response (Additional file 1: Dataset S4). Interestingly, functions related to response to nematodes, which often parasitize leguminous plants, and regulatory response to auxin and oxygen were enriched among families duplicated at the base of legumes.

### The transcriptional landscape of *P. vulgaris*

**Fig. 4:**
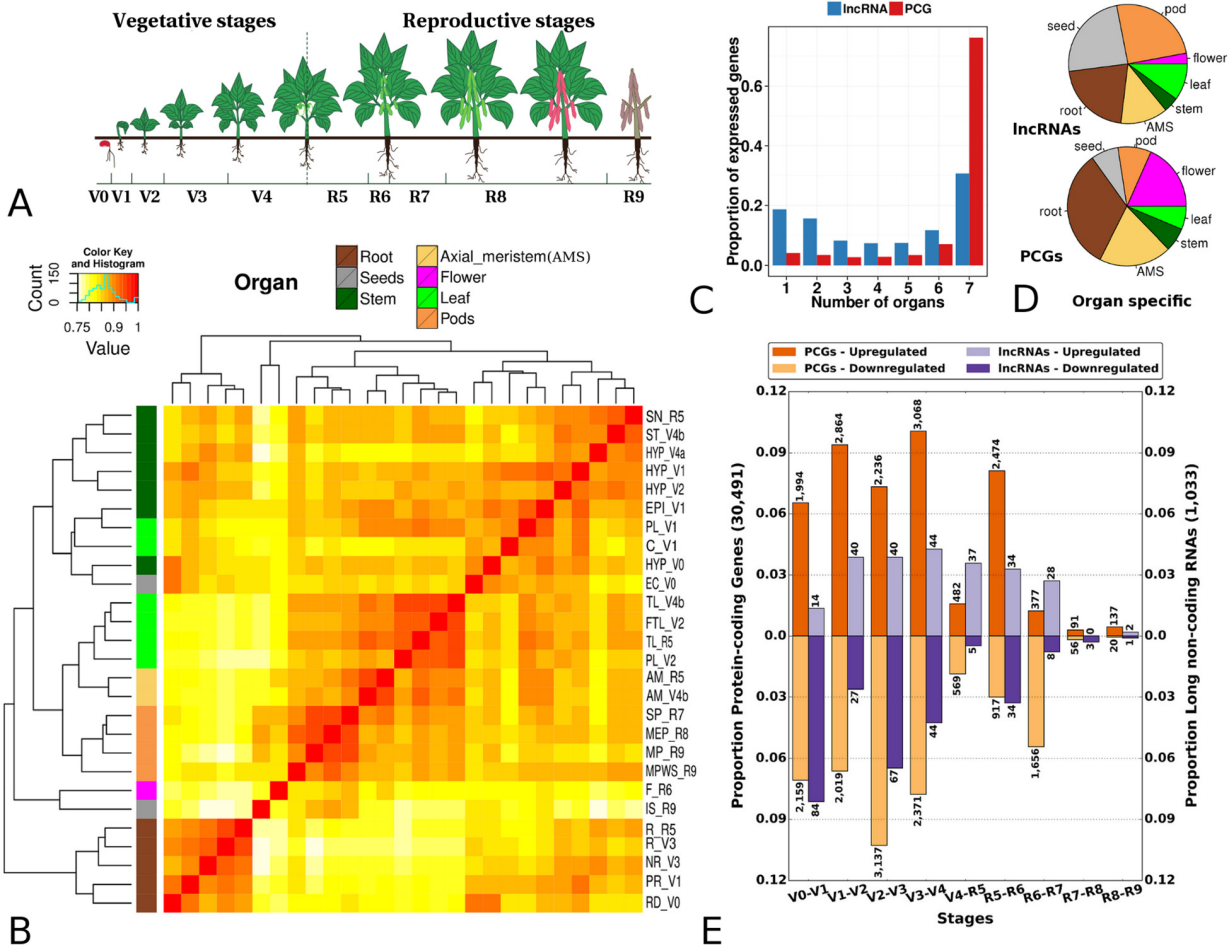
Transcriptome dynamics. **a** Development stages of the common bean. Modified with permission from the technical guide for the bean growing by the “Instituto Interamericano de Cooperación para la Agricultura” (IICA) [33]. **b** Hierarchical clustering of bean samples based on expression levels of protein coding genes (*PCG*). The sample labels are described in Additional file 1: Table S8. **c** Tissue specificity of the PCGs and lncRNA genes. The bar plot represents the proportion of genes expressed in a given number of organs **d** The pie charts represent the distribution of organ-specific PCG and lncRNAs across organs. The color code for organs is specified in (**b**). **e** Differential PCG and lncRNA expression during development. Each bar corresponds to the number of genes differentially expressed in a given developmental stage compared with the previous one. Values above and below zero indicate the proportion of up-regulated and down-regulated genes, respectively; the number of regulated genes is shown at the tip of the corresponding bar

We used RNA-Seq libraries from 27 organs/developmental stages for which we have technical replicates (7 of the 34 conditions only had one sample) to generate a gene expression atlas across organs and during plant development. Libraries were classified into seven organs (root, leaf, seed, pod, stem, flower and axial meristem) and into developmental stages (V0–R9, expanding from 48 hours to 86 days) [32, 33] (Fig. 4a; Additional file 3: Dataset S5). Hierarchical clustering of the samples based on PCG expression recapitulates tissue types, the main separation being between the root and aerial samples (Fig. 4b). This separation was recapitulated when we included in the analysis 21 samples from leaves from different accessions in Bellucci et al. [34], and 24 samples from seven organs in O’Rourke et al. [35] (Additional file 1: Figure S9). Hierarchical clustering based on lncRNA expression also recapitulates tissue type, but in this case pods and seeds are clearly separated from the rest of the tissues (Additional file 1: Figure S10). At a threshold of gene expression of 1 RPKM, we identified 20,525 (67 %) PCGs, and 521 (52 %) lncRNAs expressed in at least one organ (Additional file 1: Table S30; Additional file 3: Datasets S6 and S7), and 12,261 (40 %) PCGs and 99 (10 %) lncRNAs were expressed in all organs. On average, we detected 64 % of PCGs and 28 % of lncRNAs expressed per organ (Additional file 1: Figures S11 and S12).

We defined putative PCGs as house-keeping genes when they were within the top 10 % of the expressed genes with lowest coefficient of variation across all samples (Additional file 4: Dataset S8). This resulted in 2811 genes. GO analysis revealed that these genes preferentially carry out functions related to fundamental cell processes (Additional file 4: Datasets S9–S11). Using orthology predictions derived from the phylome, we compared this set with the two previously defined sets of legume housekeeping genes: 1000 soybean genes [36] and ~2500 genes from the common bean transcription atlas [35] (Additional file 1: Figure S13). Remarkably only 195 genes are common between the three sets, and only half (1279 genes) are common between the two common bean sets. This reflects either low conservation of housekeeping genes or, most likely, the reduced number and divergent set of conditions in which transcription has been monitored in these studies. Further, we identified a core set of 25 lncRNA genes that are both ubiquitously expressed in all organs and evolutionarily conserved in at least seven of the twelve species used for comparative analysis and thus may play crucial roles similar to those played by housekeeping PCGs. In general, highly conserved lncRNAs tend to have a higher level of expression (Additional file 1: Figure S14).

We performed differential gene expression analysis for PCGs across all pairs of samples, both in individual samples as well as in sets of samples grouped into organs and developmental stages (Additional file 5: Datasets S12–S22). We found that 937 PCGs had organ-specific expression (details in "Material and methods"; Additional file 1: Figure S15; Additional file 4: Dataset S8), a third of them are from root samples (Fig. 4c, d). Organ-specific genes are generally enriched for functions characteristic of the physiology of the organ (Additional file 4: Dataset S10). We also found 171 lncRNAs expressed in one organ only, which represents a proportion (17 %) about four to five times higher than that measured for PCGs (4 %; Fig. 4c, d). Of these, about half (84) are fruit-specific, in contrast with organ-specific PCGs, which are enriched in roots (32 % of organ-specific PCGs are root-specific; Additional file 1: Table S30).

### Transcriptome dynamics during plant development

We compared gene expression in each stage of plant development (Fig. 4d) with the previous stage globally, as well as independently in each of the four organs where we had sufficient numbers of samples at different stages: root, leaf, stem and pooled flower/pod/seeds, referred to here in after as fruits (Additional file 1: Figure S16). Overall, a larger number of transcriptional changes occur during the vegetative as compared with the reproductive stage for both PCGs and lncRNAs (Fig. 4e). For instance, during the establishment of primary leaves, over 1000 genes are differentially expressed, including 20 lncRNAs, while this number drops to less than 120 when comparing leaves during the later stages. We found similar numbers of differentially expressed genes during root, leaf and stem development (2165, 2220 and 2859, respectively), and a larger number (4869) during fruit formation. The functions enriched in genes that are differentially expressed between different stages in each organ are consistent with the physiological changes associated with the development of that organ (Additional file 4: Data S14–S21).

We also identified 624 genes specifically expressed in a given developmental stage (Additional file 1: Figure S17; Additional file 4: Datasets S8 and S11). Genes specific to early vegetative stages (V0–V1, ~19 %) are enriched in enzyme regulator and oxidoreductase activity, whereas genes specific to late vegetative stages (V2–V4, ~20 %) are enriched in functions related to photosynthesis, cell division and defense response. Functions related to nitrogen fixation and metabolisms are enriched in early reproductive stages (R5, R6, ~46 %), while in late reproductive stages (R7, R8 and R9, ~15 %), the most enriched functions are related to cell fate determination, regulation of defense response and telomere maintenance.

### Co-expression network

**Fig. 5:**
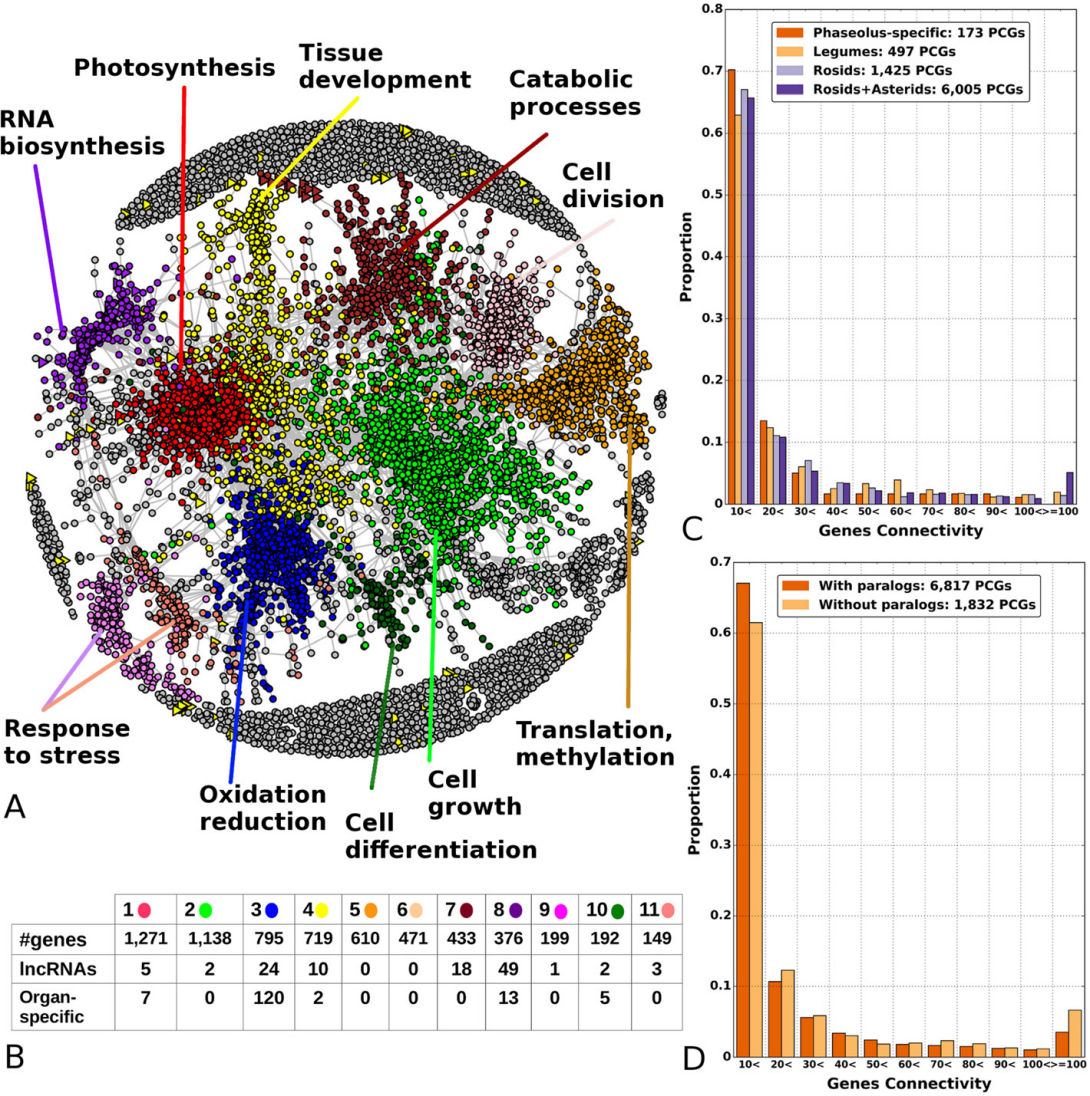
Co-expression network. **a** Co-expression network layout; the 11 largest modules are colored differently, and labeled with their putative function. **b** Composition of the largest modules in the co-expression network (number of PCGs and lncRNAs, and of organ-specific genes). Colors correspond to those in the network in (**a**). **c** Gene connectivity as a function of evolutionary age. **d** Gene connectivity as a function of presence/absence of paralogs

To provide deeper insights into the transcriptomic bases of bean cellular processes, we constructed a co-expression network and analyzed its topological properties. We used the set of 21,560 PCGs and lncRNA genes that were expressed in at least one sample at more than three counts per million (CPM; "Materials and methods"; Additional file 1). From the resulting network we selected a sub-graph that includes nodes with at least one connection and comprises 8884 genes (including 197 lncRNAs) and 81,220 edges (Fig. 5a). On average, each node in the network has 18 co-expression links; lncRNAs show a much stronger connectivity, with 30 co-expression links on average. The most connected node, plastid lipid-associated protein, has 260 connections. We found that the 125 most-connected genes (>150 links) were all inter-connected to each other, forming a dense hub. This dense hub was not observed in a random network generated with the same node degree (Additional file 1). Similar to results in *A. thaliana* [37], the most enriched GO categories of these hub genes are related to photosynthesis and NADP metabolic process. Among lncRNAs, two are highly connected — XLOC_000314 and XLOC_004014 — with 101 and 105 connections, respectively, belonging to a co-expression cluster related to synergid differentiation. XLOC_000314 is about 9 kb away from the auxin-induced 15A-like gene, which may reflect a functional relationship, since lncRNAs have been proposed to regulate the expression of nearby PCGs [23].

Genes included in the co-expression network were then analyzed considering their relative evolutionary age and number of paralogs, as inferred from the phylome. For this, we used a phylostratigraphic approach using the furthest detectable ortholog (or homolog for genes without detectable orthologs) as a proxy for the evolutionary origin of the genes. The co-expression network was enriched in ancient genes, with 75 % of the genes assigned to the oldest relative age (Additional file 1: Table S31) compared with the whole genome (~58 %). Consistently, the network was depleted in *Phaseolus*-specific genes (~2 %) with respect to the whole genome (~19 %). We then assessed whether the age and the co-expression connectivity of a gene were related (Fig. 5b; Additional file 1: Figure S18). We found that ancestral gene families were enriched among highly connected genes (>100 connections, Fisher exact test *p* value 1.9377e-12), whereas no *Phaseolus*-specific genes were present in this class. Finally, we divided genes in the network into two categories — with or without paralogs — and found that singletons had a significantly higher number of connections compared with genes with at least one paralog (22.72 versus 17.11 connections on average; t-test *p* value 1.8821e-08). Conversely, we found that most singletons were assigned to highly connected genes (>100 connections), whereas genes with few connections tended to have paralogs (Fig. 5c; Additional file 1: Figure S19). Our findings support the hypotheses that (i) older genes and (ii) genes without paralogs tend to have a broad expression and a large number of co-expression partners, whereas gene duplication leads to more specialized expression patterns, fewer co-expression partners, and therefore less constrained expression.

We used a fast-greedy community algorithm to divide the network into inter-connected modules and carried out functional enrichment analyses of the 11 modules having more than 100 genes (Fig. 5d; Additional file 5: Datasets S23 and S24). The largest module had 1271 genes with 39,041 edges and an average connectivity of 50, and included the densely interconnected hub already described above. This module has more than 170 significantly enriched GO terms (*p* value < e-5), of which most are related to photosynthesis. The second largest module (1138 genes) is related to protein localization and cell growth processes. These two modules are strongly depleted from both lncRNAs and organ-specific PCGs. The third module is enriched in genes specific to the root and, consistently, the majority of their functions are related to oxidation-reduction, flavonoid processes and root development. In module eight, we found enrichment in genes specific to pods and seeds, as well as a strong enrichment for lncRNAs. Among significantly enriched functions, we found RNA biosynthetic processes and regulation of gene expression, as well as those related to ovule and floral organ development. We studied the distribution of gene ages among these clusters (Additional file 1: Table S32) and found that all modules were enriched in ancient genes. Interestingly, modules associated with root development (module 3) and flowering activity (module 8) are enriched in legume-specific genes, with approximately two-fold enrichment with respect to the genomic average.

### Gene duplication and divergence in expression patterns

**Fig. 6:**
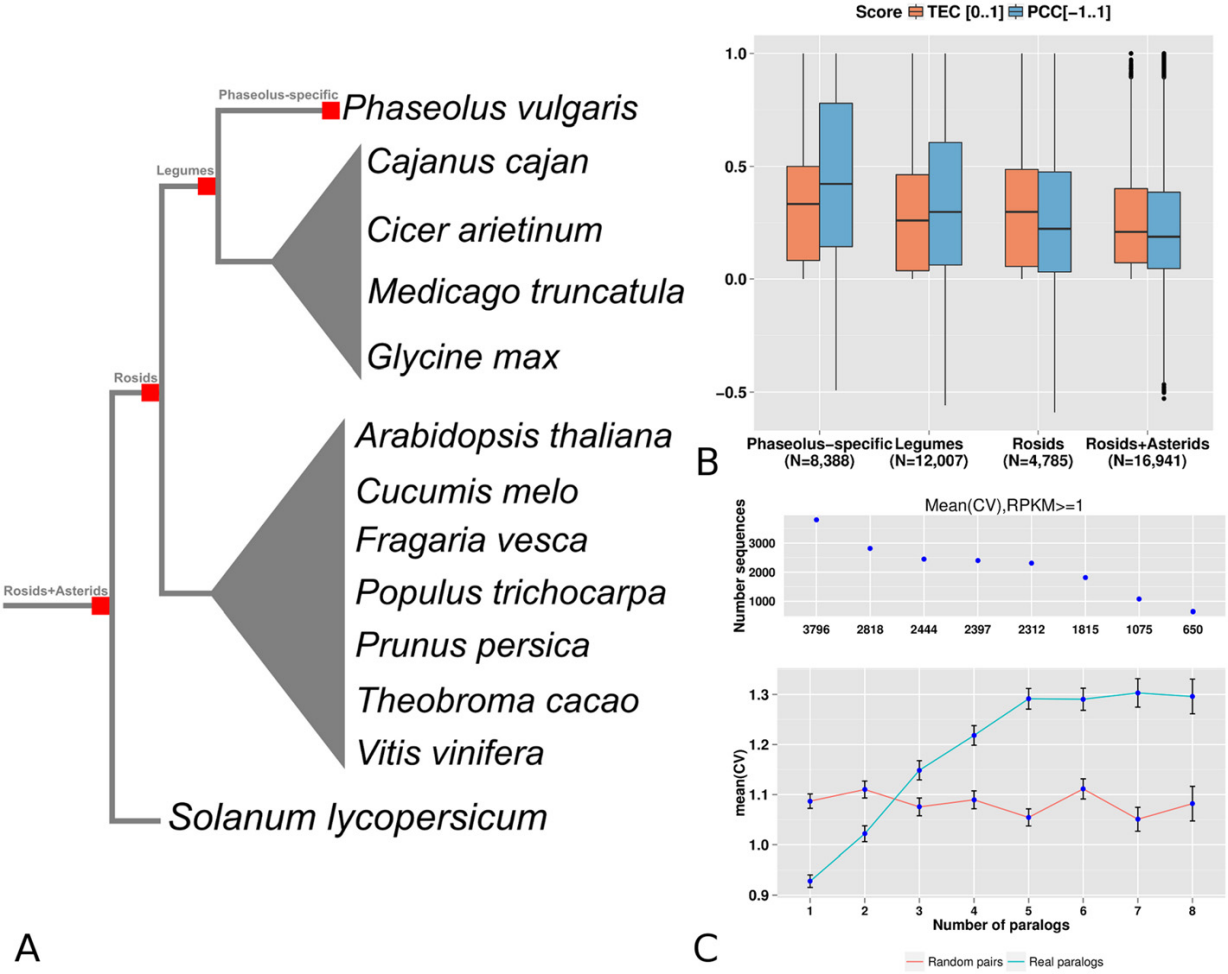
Analysis of dated duplicated genes. **a** Species list assigned to different relative evolutionary periods. *Red squares* represent a duplication event. **b** Average Pearson correlation coefficient (PCC) and tissue expression complementarity (TEC) scores computed for the proteins assigned to particular ages. The number of genes duplicated at a particular age is indicated in parentheses on the x-axis. **c** Relationship between gene expression variation and gene duplications. The *blue color* represents the mean coefficient of variation (CV) for a real set of paralogs and *red* for a randomly assigned one. The last class on the x-axis (*8*) contains eight or more paralogs

Gene duplication is considered a major source of biological functional innovation [38]. The genetic redundancy introduced by a duplication event enables the evolution of novel interactions and functions, although the underlying mechanisms of how this is achieved are poorly understood. Here, we exploited the availability of a comprehensive expression atlas and the phylome for *P. vulgaris* BAT93 to study the temporal and spatial patterns of expression diversification for genes duplicated at different evolutionary periods. In this regard, we detected and dated gene duplications by automatically scanning all bean gene phylogenies (see "Materials and methods"; Fig. 6a; Additional file 1: Table S28). For each duplication event detected we dated the time of duplication and computed the level of tissue expression divergence between the resulting paralogous genes using the Pearson correlation coefficient (PCC) and the tissue expression complementarity (TEC; see "Materials and methods") [39]. In brief, TEC measures the fraction of tissues in which only one of the two genes is specifically expressed with respect to the total number of tissues in which any of the two genes are expressed. Thus, the higher the TEC, the bigger the expression complementarity of both genes. Our results show that genes assigned to older duplication events are less correlated and have more complementary expression profiles than those assigned to younger events (Fig. 6b). We then used the coefficient of variation to quantify the fluctuations of expression levels across samples for genes with different numbers of paralogs. Our results (Fig. 6c) show that, similar to what has been observed in *Caenorhabditis elegans* and human [40], variability in gene expression increases with the number of paralogs.

## Discussion

Although the common bean (*P. vulgaris*) is one of the most important food legumes in the world [41], until very recently genomics resources available were scarce. Together with the recent sequencing of the genome of an Andean landrace [6], the phylogenetic, genomic and transcriptomic data generated in this study provide invaluable resources to understand the biology and evolution of Mesoamerican common bean, and its differences from the Andean lineage, offering new tools and methodologies to generate superior varieties.

Here we focused on the investigation of the patterns of gene expression underlying organ development and formation, and how this relates to underlying gene evolution. Overall, our results are consistent with previous analysis of the common bean transcriptome [34, 35, 42–45]. We found that about 70 % of genes exhibit modulated expression during development or across organs; with some genes being extremely highly expressed in particular stages, i.e., ribulose-bisphosphate carboxylase and storage proteins from the phaseolin families in the leaf and seed samples, respectively (RPKMs of about 50,000). Additionally, our sampling included the embryonic stage V0, which allowed the identification of genes contributing to early organ formation. Thus, we found that genes preferentially expressed in early stages of development are enriched in enzymatic and oxido-reduction functions, and that it is only later during development that photosynthetic functions are activated.

One of the main traits of *P. vulgaris* is the high protein content of its seeds. Seed and fruit development are complex processes that require coordinated expression and regulation of several genes [46, 47]. Our results show that the transcriptional changes occurring during fruit development are enriched in genes related to aleurone grain, nutrient reservoir activity, DNA replication, cell cycle, epigenetic and polysaccharide biosynthesis processes, and embryo morphogenesis. Similar results have been found in *Lotus japonicus* and other legumes [48]. Notably, our results suggest that lncRNAs may play an important role in fruit development. Indeed, organ-specific lncRNAs are preferentially expressed in the fruit. This parallels the bias observed towards lncRNA expression in mammalian gonads [49]. lncRNAs have been proposed to play a role during spermatogenesis [50], and we have actually found that the two most transcriptionally connected lncRNAs are part of a cluster related to synergid differentiation, and are thus very likely involved in synergid development. These observations could hint at an ancient program common to plants and animals involving lncRNA in sexual reproduction. Also, as in animals, bean lncRNAs show low levels of conservation: less than one-third of the transcripts are conserved beyond *Glycine max*, suggesting rapid lncRNA turnover, as reported in insects and vertebrates [51].

Organ-specific PCGs, in contrast, are preferentially expressed in the root. In particular, we found that, in this organ, PCGs involved in nitrogen fixation and nodulation are preferentially expressed in pre-flowering and flowering stages (R5 and R6, respectively), suggesting that plants may already adapt their metabolism to the symbiosis from these stages. Co-expression network analysis is a powerful approach to investigate the concerted action of genes, to infer gene functions and provide novel insights into the system-level understanding of cellular processes [52–54]. Our results suggest that the largest sets of *P. vulgaris* genes with concerted expression are involved in basic plant functions, such as photosynthesis, cell cycle, protein synthesis, etc., as previously reported [34, 37]. We also observe large modules of species-specific genes, such as those related to root development, nodulation and symbiosis. Among others, enrichment in these modules in functions related to abiotic stress, stimulus and floral development may be related to domestication [34]. Interestingly, while PCGs show stronger sequence conservation than lncRNAs, we found little overlap between the set of housekeeping genes defined here and other housekeeping gene sets, previously defined in soybean and the bean Andean landrace [35, 36], most likely because of the limited set of organs and conditions profiled in those studies.

The availability of comprehensive catalogues of evolutionary histories of genes and of the dynamics of their expression across tissues and developmental stages has enabled us to assess at a genome-wide scale, and for the first time in plants, how the number and age of gene duplications affect patterns of tissue expression. It has been hypothesized that the partitioning of gene expression in a spatial or temporal manner — a form of sub-functionalization — has played a major role in the initial retention of duplicates, because complementary expression patterns achieved through differential degeneration of the ancestral gene expression profile may render both copies indispensable [55, 56]. Further evolutionary events may result in other forms of functional diversification, including the acquisition of novel expression patterns and functional activities, so that the divergence in terms of expression is expected to increase with time.

Massive gene duplications, including those resulting from whole genome duplications, are widespread in flowering plants and constitute a driving force in angiosperm diversification and adaptation. However, in contrast to vertebrates or fungi, the diversification of genome-wide expression patterns after duplication has not been widely studied in plants. Previous work has focused on measuring expression divergence between duplicates within a given evolutionary period such as an ancient whole genome duplication [57], or globally measuring divergence between paralogs, without stratifying them by duplication periods [58]. Our results suggest an important role of gene duplication in enabling tissue and temporal specialization of genes.

In fact, the divergence in tissue expression patterns among paralogs increases both with their time of divergence, as inferred from the gene phylogeny, and with the number of paralogs in a gene family. This indicates that diversification in tissue gene expression levels accumulates with time, as duplications occur. This finding is consistent with the co-expression network analysis, in which old singletons are highly enriched among highly connected genes, while younger genes and families with many paralogs tend to be enriched in more specialized modules, less densely connected and tightly associated with a specific organ or development stage.

Given that BAT93 and G19833 genotypes derive from independent domestication events, we can assess, for the first time, whether genomic changes leading to phenotypic features characteristic of domestication predate or not their divergence. Seed size, for instance, is a phenotypic trait that differentiates domesticated accessions from their wild relatives, and also distinguishes Andean from Mesoamerican bean accessions even at the wild state (the weight of 100 seeds is 3.5–6.5 g for wild Mesoamerican beans compared with 11.6–13.9 g for wild Andean beans). Two BAT93-specific gene family expansions were found to be functionally enriched in seed development and the ubiquitination pathway, whose role in germination and seed development has been established in another legume species, *Lupinus albus* L. [59]. Even though it remains unknown if such specific expansions preceded or occurred in parallel to the domestication process in Mesoamerica, they suggest that a similar phenotype — larger seeds — has been achieved through different pathways and genetic components in the two gene pools. In contrast to this scenario, the origin of resistance gene clusters was proposed to precede the geographic separation of the wild common bean gene pools [60]. Indeed, we found that all resistance gene clusters are shared between the two lineages, suggesting they were established in their wild ancestor and that the observed differences in disease susceptibility are due to polymorphisms in these loci. Indeed the genes with higher divergence between the two lines are often involved in defense response mechanisms, supporting ongoing co-evolution with pathogens [61]. Similarly, we found that all *Phaseolus*-specific gene family expansions common to both Mesoamerican BAT93 and Andean G19833 emerged from duplications that predate the divergence of the two lineages, and thus are not the result of parallel (convergent) expansions. Other adaptations relevant for the crop, such as symbiosis with soil organisms and resistance to pathogens such as nematodes, seem to stem from innovations within the broader legume lineage. In particular we found that the two bean genotypes harbor a gene cluster whose expansion in soybean has been related to resistance to nematodes [62], which are common parasites of legumes. Although the genes from this cluster were highly expressed in both accessions, the depth of read coverage did not reveal the presence of a higher copy number in common bean. Overall these results suggest that genomic adaptations could have facilitated a broad distribution of *P. vulgaris* populations in America, making them prone to human discovery and further domestication. Moreover, *P. vulgaris* belongs to one of the two principal clades of *Phaseolus* that includes four of the five main domesticated species (i.e., *P. acutifolius*, *P. coccineus*, *P. dumosus*, and *P. vulgaris*). Species of this clade collectively flower during either the dry or rainy season, are mostly not sensitive to disturbance, and some can tolerate a long frost period (e.g., *P. coccineus*, *P. angustissimus*). Phaseolus species are distributed from southeastern Canada south through eastern USA and across southern USA to southeastern California, throughout Mexico and Central America, and in the Andean region of South America. They are broadly distributed in elevation gradients throughout this range, from lowland dry and wet forests up to pine-oak and pine forests. Thus, the commonness of some of this species may have facilitated, in part, their discovery for domestication [10, 63]. Whether the gene family expansions described in this study are *P. vulgaris*-specific or shared by other sister species should be addressed in future studies. Ultimately, sequences from additional domesticated and wild accessions, together with the genome sequences of closely related *Phaseolus* species, will be needed to disentangle with higher resolution which genome changes preceded and most likely enabled domestication or occurred concomitantly to it.

## Conclusions

We present genomic, transcriptomic, and phylogenomic data on a Mesoamerican variety of common bean, which will serve as important resources for breeders and for understanding the domestication process in this important crop. Our results comparing two independently domesticated lineages suggest that most bean-specific gene family expansions, including those involving resistance genes, predate the split of the Mesoamerican and Andean gene pools and thus predate domestication. This suggest the possibility that key pre-existing adaptations may have facilitated domestication of certain species. Our transcriptome atlas shows that lncRNAs are particularly enriched in germ-line related tissues (pods and seeds), which suggests a possible role in fruit development. Of note, the association with germ-line tissues is reminiscent of what has been described for lncRNAs in animals. More generally our results point to an important role of gene duplication in shaping differential tissue and developmental expression in plants, which parallels previous observations in animals. As gene families get larger through successive duplication rounds their expression patterns become more narrower and different from each other.

## Materials and methods

### Plant material

*P. vulgaris* BAT93 is a breeding line developed at the International Center for Tropical Agriculture (CIAT, Cali, Colombia) and derived from a double cross involving four Mesoamerican genotypes. The biological material collected for this analysis included other important accessions: Jalo EEP558 and 60 F_5_ BAT93/Jalo EEP558 intercross plants [64]. Plants were grown under greenhouse conditions and young trifoliate leaves were collected for DNA extraction. For total RNA extraction, the breeding line BAT93 was growth at ±25 °C, 80 % humidity, and 16 h light:8 h dark photoperiod (Additional file 1).

### DNA/RNA sequencing and assembly

Single-read and mate-pair libraries for BAT93 were prepared for sequencing on Roche, Illumina, SOLiD and Sanger platforms. A BAC library derived from the BAT93 line was sequenced at the Arizona Genome Institute (AGI, USA) using the automated sequencing platform ABI3730xl® (Applied Biosystems). TruSeq libraries were run on a HiSeq2000 instrument on five lanes of paired-end 100 bp sequencing reads. The reference genome sequence from BAT93 was assembled based on Roche/454, SOLiD and Sanger reads using Newbler v2.6 [65]. Assembly improvement, verification and chromosomal anchoring utilized GBS data, generated on the Illumina sequencing platform from 60 progeny of an F_5_ advanced intercross of BAT93/Jalo EEP558 (Additional file 1). BAT93 RNA-Seq libraries were prepared using the Illumina TrueSeq RNA-Seq ibrary preparation protocol. Pooled sequencing of indexed libraries was performed on the Illumina HiSeq with v3 sequencing chemistry and approximately 50 million read pairs (2 × 75 nucleotide sequencing protocol) were generated per sample. sRNA sequencing on the same samples was carried out with non-fragmented RNA. We used the Illumina small RNA v1.5 protocol and selected inserts of size 20–100 nucleotides. Pooled sequencing of indexed libraries on the HiSeq resulted in 7–11 million reads per sample (50 nucleotide single reads). Furthermore, RNA was extracted from different BAT93 samples under more than 100 biotic and abiotic stress conditions, as well as different developmental stages and sequenced using the 454-titanium platform. After two sequencing runs, we obtained 1,830,138 reads that were assembled by Newbler v2.5 into 21,628 isogroups that include 28,601 isotigs with an average length of 1047 bp (Additional file 1).

### Repeat detection

For the *de novo* predictions of repeat elements, the REPET pipeline [66] was used. The predicted LTR retrotransposon family was further refined using the programs LTRharvest [67] and LTRdigest [68]. The final prediction for LTR retrotransposons is the union of this procedure and REPET-based predictions. Homology-based transposable element identification was performed using RepeatMasker [69] against plant-specific repeat families in RepBase v.17.11 [70]. Additionally, we ran RepeatMasker v3.2.8 against plant-specific repeat families and *G. max* repeat library from RepBase to identify interspersed repeats.

### Gene annotation

For the PCG annotation, RNA-Seq reads, 454 isotigs assembled from a pyrosequenced normalized cDNA library and ESTs/mRNAs present in GenBank [71], and proteins from Uniprot [72] were aligned to the genome. *Ab initio* gene prediction software, GeneID, SGP2, AUGUSTUS and GlimmerHMM [73–76], were first trained using a set of PASA training set candidates and then run on the reference assembly. All these sources were combined with Evidence Modeler [77] into consensus PCG models, which were passed through two rounds of annotation updates using PASA to add untranslated regions and alternative splicing variants.

Functional annotation was performed using an in-house developed pipeline which performs an electronic inference of function that is based on the sequence similarity between the bean predicted proteins and known proteins in different public repositories: InterPro [78], KEGG [79], Reactome [80], SignalP [81], PhylomeDB [24] and Blast2GO [82].

Plant disease resistance genes were predicted by two methods: 733 genes were annotated by using the Disease Resistance Analysis and Gene Ontology (DRAGO) pipeline [83]; and 120 resistance genes were identified by the presence of a NB-ARC domain in their sequences for a final set of 852 genes (Additional file 1).

### Long non-coding RNA

Homology-based lncRNAs were predicted in bean taking *A. thaliana* lncRNA transcripts as templates. These were compared using blast [84] against the masked bean assembly and the hits were then used as anchor points to realign the *A. thaliana* queries with surrounding genomic regions using exonerate [85] as a split aligner. Final conservation was estimated on T-Coffee [86] pairwise re-alignments between the query and its predicted spliced model. *Ab initio* lncRNA models were predicted using Cufflinks, and then Cuffmerge [87] was used to combine transcript models from all samples into a single set of consensus models. Single-exon models, transcripts within 1 kb of coding regions, and putative ORFs were filtered out [23]. Sets of transcripts overlapping by at least 1 nucleotide were clustered into gene models. Sequence conservation of transcripts was determined applying the procedure described above for homology-based prediction to the 12 plant genomes using all bean transcript models as templates. lncRNA transcript expressions were obtained using the Flux Capacitor [88].

### Small non-coding RNA

Small non-coding RNAs were predicted using the CMsearch tool from the Infernal package (v.1.1rc2) [89]. An E-value cutoff of 0.01 allowed detection of 2529 non-overlapping hits; of these, 258 are in contigs and 2271 in scaffolds. We were able to classify 2371 of them into different general categories as shown in Table S22 in Additional file 1.

### Transcriptome analysis

Reads were independently aligned to the reference *P. vulgaris* assembly v10 using the GEMtools RNA-Seq pipeline v1.6.2 [90]. On average, 89 ± 5 % of the reads were mapped across samples, 69 ± 10 % of the reads mapping uniquely. Flux Capacitor v1.2.4 [88] was used to quantify genes, transcripts, exons and splice junctions in each sample separately; expression levels are given in reads per kilobase per million mapped reads (RPKM) [91] and in read counts. For the differential expression analysis and co-expression network construction we have normalized read counts into counts per million (CPM). In addition, to quantify annotated elements, we built *de novo* contigs by merging overlapping RNA-Seq reads. Cumulatively across all samples, 85 % of exonic, 75 % of intronic and 5 % of intergenic nucleotides were covered by contigs. To identify the organ-specific PCGs we calculated average expression values for each organ; genes having average RPKM ≥ 0.1 in a given organ and less in all others were considered organ-specific. The same procedure was performed to identify stage-specific genes. Differential expression was estimated with the software package edgeR (R v3.0.1, edgeR v3.2.4) [92]. Hierarchical clustering analysis of the expression profiles were performed using the hclust command in R and default complete linkage method. The GO and enrichment analyses were performed using the Blast2GO [82] and topGO [93] with a false discovery rate ≤ 0.05. The bean co-expression network was constructed using the entire set of PCGs and lncRNA genes. Genes with low expression (<3 CPM) were filtered out. In total we used 21,560 genes for the initial network construction. Gene expression values were log-transformed and the resulting expression matrix was scaled along both the genes and the samples; pairwise PCC was calculated between all pairs of genes. Graphical Lasso [94] was used to construct the network. The graph was drawn using the Fruchterman-Reingold layout [95]. Downstream analyses were performed on the sub-networks with more than one edge between nodes. The network was subdivided by using a fast-greedy community algorithm [96].

### Phylogenetic and comparative analysis

The database used for the phylome reconstruction contained 30,405 unique protein sequences for common bean. The resulting phylome comprises 27,986 gene trees, representing 92 % of the predicted proteins. To build the gene trees, a Smith-Waterman search was used to retrieve homologs of each bean protein. These homologous sequences were aligned using MUSCLE v3.8 [97], MAFFT v6.712b [98], and KAlign v2.08 [99] and then the resulting alignments were combined using M-Coffee [100] and trimmed with trimAl v1.4 [101]. Phylogenetic trees based on the maximum likelihood approach were inferred from these alignments. Maximum likelihood trees were reconstructed using the two best-fitting evolutionary models. The evolutionary models best fitting each protein family were selected using BioNJ [102] and PhyML v3 [103]. Orthology and paralogy relationships among *P. vulgaris* genes and those encoded by the other considered genomes were inferred using a phylogenetic approach, implemented in ETE v2 [104]. The resulting orthology and paralogy predictions can be accessed through http://phylomedb.org/ (Additional file 1). Two additional phylomes following the same strategy were reconstructed to include in the comparative analyses the *P. vulgaris* G19833 genome. One of the phylomes was reconstructed using the *P. vulgaris* BAT93 genome as reference while the other one was reconstructed using the *P. vulgaris* G19833 genome as the reference. For all analysis we used v.218 of G19833 obtained from Phytozome v10 [105]. Phylomes have 30,405 and 27,126 bean unique proteins which led to 28,075 (92.34 %) and 26,304 (96.97 %) reconstructed single trees, respectively. We used these two additional phylomes to predict orthology relationships among proteins from both genomes. One-to-one orthologs were used to compute the level of similarity in terms of gene content among bean genomes. Additional gene pairs were added in cases (1) where identical sequences were found in both genomes, (2) with perfect gene order conservation in terms of linkage group/chromosomal placement and surrounding genes, and (3) of single genes which have more than one orthologous gene in the counterpart genome without those genes being linked to any other genes. We aligned those gene pairs using MAFFT v6.712b [98] and analyzed those for which the sequence identity was lower or equal to 0.95 before and after removing gaps. Analyzing only homologous sites, e.g., without gaps, avoids any bias introduced by the different gene annotation strategies followed in each project.

To identify regions in the Andean genome absent in the Mesoamerican one, we mapped the BAT93 genomic Illumina reads into the G19833 genome. Reads were aligned with BWA-mem v0.7.12 [106] using default parameters. Read coverage was computed for each base in G19833 (i.e., the number of reads overlapping a given base). We found 10,193 regions ranging from 1 to 1130 kb with continuous zero coverage. These regions contained 314 genes and were distributed equally across all chromosomes and some unplaced scaffolds.

Single-gene trees from BAT93 phylomes were scanned to detect and date duplication events using a previously described algorithm [26]. Duplications events were assigned to four different relative evolutionary periods: basal to *P. vulgaris*, basal to legumes, basal to rosids, and basal to the split of rosids and asterids. Only events including the seed protein of each gene tree were considered for downstream analyses. Expression data for pairs of duplicated bean proteins together with their assigned relative age were used for computing the PCC and the TEC scores. The number of paralogous sequences to the seed protein of each single tree was also computed. The mean coefficient of variation (CV) for the expression data was computed grouping proteins according to the number of paralogs detected. Finally, speciation events detected for single-gene trees in the BAT93 phylome were used to date bean proteins. The furthest orthologous sequence, according to the previously mentioned ages, was selected as the age of each seed protein. We dated 24,098 proteins (~79 %) using this approach. For the remaining proteins, the relative age was assigned after detecting the most distant homologous sequence among the BLAST results. In this particular analysis, the limit of 150 sequences was ignored.

### Data availability

Raw sequence reads and quality scores were deposited in the Sequence Read Archive (SRA) of the National Center for Biotechnology Information (NCBI). Primary accession numbers: PRJNA221782 (BioProject ID); SRS488731 (genomic 454, SOLiD and HiSeq reads); SRS488023, SRS488025, SRS489191-255 (GBS HiSeq reads); and SRS498664, SRS498673-76, SRS498904-933 (RNA-Seq HiSeq reads). The *P. vulgaris* BAT93 genome assembly is available at NCBI Whole Genome Shotgun database under accession number LPQZ00000000. Additionally, unmasked sequence data and annotations are available at the CoGe database (https://genomevolution.org/CoGe/SearchResults.pl?s=20365) under Genome ID 20365. The BAT93 genome and transcriptome can be accessed and browsed at http://denovo.cnag.cat/genomes/bean. The entire set of the linkage groups with anchored markers can be viewed at http://phasibeam.crg.eu/wiki/LinkageGroups. All phylogenetic trees and alignments of the three *P. vulgaris* phylomes are publicly available through phylomeDB (http://www.phylomedb.org/, phylome ids 8, 9, and 10).

### Ethics approval

Ethics approval was not required for the study.

## Additional files

Additional file 1:
**Supplementary text, Figures S1–S19, Tables S1–S32, supplementary dataset descriptions.** (PDF 5381 kb) Additional file 2:
**Supplementary datasets S1–S4.** (XLS 125 kb) Additional file 3:
**Supplementary datasets S5–S7.** (XLS 20553 kb) Additional file 4:
**Supplementary datasets S8–S11.** (XLS 395 kb) Additional file 5:
**Supplementary datasets S12–S24.** (XLS 2190 kb)

## Abbreviations

BAC: Bacterial artificial chromosome
CPM: counts per million
EST: Expressed sequence tag
GBS: Genotyping-by-sequencing
GO: Gene Ontology
lncRNA: Long non-coding RNA
LTR: Long terminal repeat
ORF: Open reading frame
PCC: Pearson correlation coefficient
PCG: Protein coding gene
RPKM: Reads per kilobase per million mapped reads
sRNA: Small RNA
TEC: tissue expression complementarity
