# Supplementary material for: Genome and transcriptome analysis of the Mesoamerican common bean and the role of gene duplications in establishing tissue and temporal specialization of genes
Source: Genome Biol. 2016 Feb 25;17:32. doi: 10.1186/s13059-016-0883-6 (PMC4766624; doi:10.1186/s13059-016-0883-6)
Supplement: Additional file 1: — Supplementary text, Figures S1–S19, Tables S1–S32, supplementary dataset descriptions. (PDF 5381 kb) [file 13059_2016_883_MOESM1_ESM.pdf]

Supplementary materials

The genome and the transcriptome analysis of the Mesoamerican common bean and the role of gene duplications in establishing tissue and temporal specialization of genes.

Anna Vlasova[1,2,24], Salvador Capella-Gutiérrez[1,2,3,24], Martha Rendón-Anaya[4,24], Miguel Hernández-Oñate[4], André Minoche[5], Ionas Erb[1,2], Francisco Câmara[1,2], Pablo Prieto-Barja[1,2], André Corvelo[6], Walter Sanseverino[7], Gastón Westergaard[8], Juliane C. Dohm[9], Georgios Joanis Pappas Jr[10], Soledad Saburido-Alvarez[4], Darek Kedra[1,2], Irene Gonzalez[2,11], Luca Cozzuto[1,2], Jessica Gómez-Garrido[2,12], María A. Aguilar-Morón[2,11], Nuria Andreu[2,11], O. Mario Aguilar[13], Jordi Garcia-Mas[7], Maik Zehnsdorf[2,11], Martín P. Vázquez[8], Alfonso Delgado-Salinas[14], Luis Delaye[15], Ernesto Lowy[16], Alejandro Mentaberry[17], Rosana P. Vianello-Brondani[18], José Luís García[19], Tyler Alioto[2,12], Federico Sánchez[20], Heinz Himmelbauer[9], Marta Santalla[21], Cedric Notredame[1,2], Toni Gabaldón[1,2,22], Alfredo Herrera-Estrella[4] , and Roderic Guigó[1,2,23.]

Affiliations:

[1]Bioinformatics and Genomics Programme, Centre for Genomic Regulation (CRG), Dr. Aiguader 88, 08003 Barcelona, Spain. [2]Universitat Pompeu Fabra (UPF), Dr. Aiguader 88, 08003 Barcelona, Spain. [3]Yeast and Basidiomycete Research Group. CBS Fungal Biodiversity Centre. Uppsalalaan 8. 3584 LT Utrecht, The Netherlands. [4]Laboratorio Nacional de Genómica para la Biodiversidad, Centro de Investigación y de Estudios Avanzados del IPN (Cinvestav), 36821 Irapuato, Guanajuato, Mexico. [4]Genomics Unit, Centre for Genomic Regulation (CRG) and Universitat Pompeu Fabra (UPF), 08003 Barcelona, Catalonia, Spain. [5]Garvan Institute of Medical Research,384 Victoria Street, Sydney NSW 2010, Australia [6]New York Genome Center, 101 Avenue of the Americas, New York, NY 10013, USA [7]IRTA, Centre for Research in Agricultural Genomics (CRAG) CSIC-IRTA-UAB-UB, Campus UAB, 08193 Bellaterra, Barcelona, Catalonia, Spain. [8]Instituto de Agrobiotecnología Rosario (INDEAR), Rosario, Santa Fe 2000, Argentina. [9]Universität für Bodenkultur, Muthgasse 18, 1190 Vienna, Austria [10]University of Brasilia, Biological Science Institute, Department of Cellular Biology, Brasília, DF, 70790-160, Brazil. [11]Genomics Unit, Centre for Genomic Regulation (CRG), Dr. Aiguader 88, 08003 Barcelona, Catalonia, Spain. [12]CNAG-CRG, Centre for Genomic Regulation (CRG), Dr. Aiguader 88, 08003 Barcelona, Spain [13]Instituto de Biotecnología y Biología Molecular (IBBM), UNLP-CONICET, 1900 La Plata, Argentina. [14]Departamento de Botánica, Instituto de Biología, Universidad Nacional Autónoma de México. 04510 Mexico City, Mexico. [15]Departamento de Ingeniería Genética, Unidad Irapuato, Cinvestav. 36821 Irapuato, Guanajuato. Mexico. [16]European Molecular Biology Laboratory, European Bioinformatics Institute, EMBL-EBI, Wellcome Trust Genome Campus, Hinxton, Cambridge

CB10 1SD, UK. [17]Facultad de Ciencias Exactas y Naturales, Universidad de Buenos Aires (UBA), C1428EGA Buenos Aires, Argentina. [18]EMBRAPA Rice and Beans, Biotechnology Laboratory, Santo Antônio de Goiás, GO, 75375-000, Brazil. [19]Environmental Biology Department, Centro de Investigaciones Biológicas, (CSIC). 28040 Madrid, Spain. [20]Depto. de Biología Molecular de Plantas, Instituto Biotecnología, Universidad Nacional Autónoma de México. 62210 Cuernavaca, Morelos, Mexico. [21]Mision Biológica de Galicia (MBG)-National Spanish Research Council (CSIC). 36080 Pontevedra, Spain. [22]Institució Catalana de Recerca i Estudis Avançats (ICREA), Pg. Lluís Companys 23, 08010 Barcelona, Spain. [23]IMIM (Hospital del Mar Medical Research Institute), 08003 Barcelona, Spain.
[24]These authors contributed equally to this work.

Corresponding authors: roderic.guigo@crg.eu; aherrera@langebio.cinvestav.mx; toni.gabaldon@crg.eu

R.G. Center for Genomic Regulation, C/ Dr. Aiguader 88, E-08003, Barcelona, Catalonia, Spain. Fax: +34 93 3969983 Phone: +34 93 3160110
email: roderic.guigo@crg.eu
A.H.-E. Laboratorio Nacional de Genómica para la Biodiversidad, Cinvestav-Irapuato. CP 36821, Irapuato, Guanajuato, Mexico. Fax (52)/462/6245849 Phone: 52/462/1663041
e-mail: aherrera@langebio.cinvestav.mx
T.G. Center for Genomic Regulation, C/ Dr. Aiguader 88, E-08003, Barcelona, Catalonia, Spain. Fax: +34 93 3969983 Phone: +34 93 3160281
email: toni.gabaldon@crg.eu

Materials and Methods

Plant Material

1. BAT93 accession

BAT93 is a breeding line developed at the International Center for Tropical Agriculture (CIAT, Cali, Colombia) and derived from a double cross involving four Mesoamerican genotypes: Veranic2 (G3709)/Tlalnepantla 64 (G1320)//Jamapa (G3645)/Tara (G5478). BAT93 was selected as our reference genome because 1) it presents multiple resistance genes against pathogens such as the Bean Common Mosaic Virus (BCMV; *I* gene), *Xanthomonas campestris* pv. *phaseoli* (causing common bacterial blight), *Uromyces appendiculatus* (causing rust), *Colletotrichum lindemuthianum* (causing anthracnose) and *Phaeoisariopsis griseola* (causing angular leaf spot) [1, 2]; 2) it is the female parent of the recombinant inbred population BAT93/Jalo EEP558, the core common bean mapping population [3] that has been useful to trace several QTL. Plants were grown under greenhouse conditions and young trifoliate leaves were collected for DNA extraction.

2. F_5 BAT93/Jalo EEP558 intercross

The high-resolution genetic map used for anchoring the scaffolds was derived from a cross between the inbred line BAT93 and the Andean genotype Jalo EEP558 [4]. For the establishment of a new set of recombinant inbred lines (RILs) the population was advanced by single-seed descent (SSD) unto the F_5 generation at the Embrapa Rice and Beans (Brazil) in a greenhouse under controlled conditions. Genomic DNA from the parents and the RILs was isolated from fresh young trifoliate leaves using the commercial DNeasy Plant kit® (Qiagen).

3. RNA samples. Total RNA extraction

We used the breeding line BAT93 grown at ±25ºC, 80% humidity, and 16h light: 8h dark photoperiod. Samples of embryo, cotyledon, radicle, hypocotyl, epicotyl, stem, leave, flower, pod, seed, and root were separately collected according to the stages of plant development [5] relative to the time of sowing (DAP) (Table S7). Each of these samples was represented by a bulk of one up to twelve individual plants ground together in liquid nitrogen. For each sample, 100 mg of ground material was added to 1ml of TRIzol (TRIzol® ReagentKit, Invitrogen), following manufacturer’s instructions, except for flower samples for which the cetyltrimethylammonium bromide (CTAB) protocol was used. The quality and quantity of total isolated RNA were checked by gel electrophoresis and spectrophotometer (Nanodrop 2000c, Thermo Scientific).

Genomic/transcriptomic sequencing

1. Genomic BAT93 sequencing

Preparation of sequencing libraries (single reads, mate-pairs) for sequencing on Roche, Illumina, SOLiD and Sanger platforms was performed as recommended by the manufacturers of these sequencing platforms.

2. BAC library and ends sequencing

A BAC library derived from the BAT93 line developed by Kami et al[6] and made available for commercial purpose was sequenced at the Arizona Genome Institute (AGI, USA). This library was constructed using HindIII inserts with an average size of about 125 Kbps and covering 20X of the genome. A total of 30,000 BAC clones were randomly selected and individually sequenced from both the forward and reverse ends using the automated sequencing platform ABI3730xl® (Applied Biosystems) as described by Kim et al [7].

3.Illumina transcriptome sequencing

RNA-Seq libraries were prepared using the Illumina TrueSeq RNA-Seq library preparation protocol (non-directional method). Briefly, following polyA+ selection, mRNA was fragmented, and used to generate double stranded cDNA by random priming. End-repair and A-tailing was followed by adapter ligation and library inserts with a peak insert size of 130-190 nt were selected. Pooled sequencing of indexed libraries was performed on the Illumina HiSeq with v3 sequencing chemistry and approximately 50 million read pairs (2 x 75 nt sequencing protocol) were generated per sample. Small RNA sequencing on the same samples was carried out with non-fragmented RNA. We used the Illumina small RNA v1.5 protocol and selected inserts of size 20-100 nt. Pooled sequencing of indexed libraries on the HiSeq resulted in 7-11 million reads per sample (50 nt single reads).

4. 454 transcriptome sequencing

We sequenced the transcriptome of *P. vulgaris* breeding line BAT93 by 454 pyrosequencing. RNA was extracted from different samples under more than 100 biotic and abiotic stress conditions, as well as different developmental stages. From samples listed in Tables S9-S11 equimolar quantities of RNAs were pooled to create two normalized libraries that were sequenced using the 454-titanium platform.

After two sequencing runs, we obtained 1,830,138 reads that were assembled (Newbler v2.5 http://454.com/products/analysis-software/index.asp, default parameters) into 21,628 isogroups that

include 28,601 isotigs with an average length of 1,047 bp; form these assembled transcripts, 24% showed splicing variants (Table S12). The comparison of the transcriptome sequences and the genomic scaffolds of BAT93, revealed that 99.6% of the assembled isotigs could successfully map a genomic region. Additionally, the isotigs were blasted against various plant databases and NCBI-NR, showing a large number of hits with other legumes such as *Glycine max*, *Medicago truncatula* and *Lotus japonicus*. Only 773 isogroups had no significant hit with any predicted gene or protein.

Genome assembly

1. P. vulgaris BAT93 assembly

We assembled a reference genome sequence from the *P. vulgaris* genotype BAT93 based on Roche/454, SOLiD and Sanger reads using Newbler v2.6 (Roche) (Table S1). Roche/454 sequencing reads underwent trimming for the emulsion PCR primer sequence 5´-CAAGGCACACAGGGGATAGG-3'. Redundant MP and paired-end data were removed based on their map positions in a pre-assembly. SOLiD MPs were only used for the scaffolding step. SOLiD MPs were also removed based on mapping coordinates as above. The final Newbler assembly was calculated from 32,055,678 Roche/454 single reads, 2,356,123 8kb Roche/454 MPs, 347,562 10kb Roche/454 MPs, 308,966 20kb Roche/454 MPs, 7,783,597 3kb SOLiD MPs, 3,987,667 5kb SOLiD MPs, 20,840 Sanger BAC-end read pairs, and 171,654 Sanger genomic read pairs from a library with insert size 1.3 kb.

2. Assembly correction

Correction of consensus errors in the final Newbler assembly was performed using BAT93 genomic Illumina reads (45-fold genome coverage of raw data). Reads were filtered according to Minoche et al., [8] and Dohm et al., [9] Filtered reads were aligned to the assembled genome with BWA v.0.6.2 [10] allowing 3 mismatches. Variant calling was performed with SAMtools mpileup v.0.1.18 [11], and only uniquely mapped reads were considered. We corrected homopolymer consensus errors with length 3 nt or larger, covered by 10 or more reads (both in forward and reverse directions), and at least 60% of reads supporting an alternative correct sequence. Intra-scaffold gaps were closed with GapCloser (SOAP v.1.3 package) [12] on the consensus-corrected assembly using Illumina BAT93 reads (Tables S2 and S3).

Additional assembly improvement and verification was done by utilized genotyping-by-sequencing data, generated on the Illumina sequencing platform from 60 progeny of an F_5 advanced intercross of (BAT93/ Jalo EEP558). Each line was sequenced to an average coverage of 6.7-fold. We first utilized 35 lines to assign genotype profiles to scaffolds and to detect misassemblies (Figures S1 and 1a) The entire set of 60

lines was used for scaffold ordering. Genomic paired-end reads were filtered as above, and aligned onto the consensus-corrected, gap-closed reference assembly using BWA v0.5.8 (3 mismatches tolerated). Only uniquely matching read pairs of correct distance and orientation were considered further. Picard v1.48 tools (http://picard.sourceforge.net) were used to merge, sort and remove duplicate reads; indels were re-aligned using GATK v1.6.5 [13]; we performed variant calling with SAMtools mpileup v. 0.1.16 and GATK v1.6.5. The resulting variants were filtered to exclude false-positives calls by applying the "HARD_TO_VALIDATE" filter; we also adjusted the read depth thresholds depending on the sample coverage. Variant clusters within flanking windows of 20 bp were excluded. Positions identified as variants between BAT93 Illumina reads and the BAT93 reference assembly were not taken into account. We used a sliding window of 10kb for scaffolds with length >=100kbp and a sliding window of 1kbp for scaffolds below 100 kb; genotype scores were assigned to all scaffolds larger than 20 kb. Then, a genotype profile was constructed by aggregation of all genotypes from all lines for each scaffold. Unambiguous profiles were assigned to 1,908 scaffolds. Scaffolds with a profile change at the same position in more than 20 lines were considered as misassembly candidates, and 48 scaffolds were edited following visual inspection. Broken scaffolds were newly connected using SSPACE v.2.0 [14] if supported by read pairs and GBS data.

3. Chromosomal anchoring

Chromosomal anchoring of the assembly was performed using 827 publically available markers, which had been located on an integrated *P. vulgaris* genetic map (http://phaseolusgenes.bioinformatics.ucdavis.edu/), together with the genotype profiles obtained by using 60 GBS lines as described above. The markers were aligned against the BAT93 assembly with Blastn (e-value cutoff 1e-15); 688 markers aligned to unique positions within 399 scaffolds. These scaffolds were used as seeds, and their profiles were compared to all other profiles, tolerating at most three mismatches. Scaffolds with identical or very similar patterns were clustered and were assigned to the same linkage group as the respective seed scaffolds. The genotype profiles were then converted into MapMaker format, and genetic maps were constructed using AntMap v.1.2 [15] (Table S4).

Annotation

1. Repeat masking

Transposable element (TE) annotation was performed on the assembly v.9.0 by a combination of software tools and refined by manual inspection. For the de novo predictions, based on the identification of repetitive signatures dispersed across the genome, the REPET [16] pipeline was used. This pipeline initially compares the genome sequence against itself, with similar segments being clustered and used to generate multiple alignments and deriving respective consensus regions. Following this procedure a repeat database was built and used to rescan the genome to refine the discovery process and to categorize the bona fide TE copies according to a recommended hierarchical classification framework [17].

The predicted LTR retrotransposon family was further refined using the programs LTRharvest [18] and LTRdigest [19]. These predicted LTR retrotransposons based on characteristic structural features, such as long terminal repeats and distinctive protein domains. The final prediction for LTR retrotransposons is the union of this procedure and REPET-based predictions.

Homology-based TE identification was performed using RepeatMasker [20] against plant-specific repeat families in RepBase [21] (version 17.11).

The collection of TE elements from the assembly v.9.0 was mapped into the assembly v.10.0 using the BLAT[22] and Blastn [23] alignment tools with the low complexity filter turned off. All families from the repeat database built on the v9.0 assembly, as described above, were successfully identified and localized in the v.10.0 assembly, with 33% of the *P. vulgaris* genome being spanned by transposon elements (Table S6). Additionally, we ran RepeatMasker v3.2.8 against plant-specific repeat families and *G. max* repeat library from RepBase to identify interspersed repeats.

2. Protein-coding gene annotation

First, RNA-Seq reads from 34 tissues (Table S8) were aligned with GEM [24] to the reference genome. Cufflinks models [25] derived from these alignments, along with isotigs assembled from a pyrosequenced normalized cDNA library and ESTs/mRNAs present in Genbank, were aligned and assembled on the genome by the Program to Assemble Spliced Alignments (PASA r2012-06-25) [26]. The *P. vulgaris* assembly was masked using a custom *P. vulgaris* repeat library combined with the glycine max RepeatMasker library (RepBase 06/04/2009)[21]. Then *ab initio* gene prediction software (GeneID, SGP2, AUGUSTUS and GlimmerHMM) [27–30] were first trained using a set of PASA training set candidates filtered by BLAST search against nr for full-length coding sequences and then run on the reference assembly(Table S13). Proteins from Uniprot were aligned to the genome. Then, PASA assemblies, gene predictions and protein alignments were combined with Evidence Modeler (EVM r2012-06-25)[25] into consensus protein coding gene models, which were passed through two rounds of annotation updates using PASA to add UTRs and alternative splicing variants(Table S14).

Initial gene set was filtered out in order to remove genes with PFAM [31] domains related to transposon activity, no homologs in other plants and without RNA-Seq data support. The final gene set comprised a total of 66,634 predicted transcripts (54,109 proteins) in 30,491 protein-coding genes(Table S15).

3. Functional annotation

Functional annotation was performed by using in-house developed pipeline (Figure S2). The pipeline performs an electronic inference of function that is based in the sequence similarity between the bean predicted proteins and known proteins in different public repositories: InterPro[32], KEGG [33], Reactome [34], PhylomeDB [35] and Blast2GO [36] Additionally, SignalP [37] was used to predict the presence and location of signal peptide cleavage sites. In total 62,713 (94.12%) transcripts and 26,635 (87.35 %) genes had some type of functional annotation (Tables S16-S17, Figure S3).

We used InterProScan (v5)[38] to inspect the bean proteins for signatures using all available InterPro databases and applications. Blast2GO was used running a BLAST search against the NCBI non-redundant (NR) collection of protein sequences (release 2013-04) and mapping the obtained hits to existing annotation associations. This analysis was run with the local p2gpipe v2.5.0 with databases go_201403 (March 2014) and assigned a description to 47,254 (71%) of the transcripts. KEGG Automatic Annotation Server (KAAS) was used to map each protein sequence into KEGG orthology (KO) groups by using a bi-directional best hit (BBH) method. Analysis was performed against a representative gene set from 26 different species, including *Glycine max*, *A. thaliana* and *Oryza sativa*. KO identifiers were then used to retrieve the relevant functional annotation using the KEGG REST-based API service. In total 16,123 (24%) transcripts were assigned to 3,522 different KO groups. In order to derive Reactome annotations

we used the 109,173 one-2-one orthologs identified in 14 different plant species that corresponded to 18,343 Bean proteins. Orthology-based derivation was performed using the PhylomeDB. We used orthologs Uniprot identifiers in order to scan, using the Reactome URL/XML query system, the Reactome DB (version 45, June 2013) and obtain the reaction, complex and pathway in which the query proteins are involved.

4. Small non-coding RNAs annotation

To detect small structured non-coding RNAs in the bean genome, we used the CMsearch tool from the Infernal package (version 1.1rc2) [39]. We scanned the genome looking at every RNA model stored in the Rfam database (version 11) [40]. Overlapping hits were removed by selecting the hit with the lowest E-value. Setting an E-value cut-off of 0.01 allows to detect 2,717 non-overlapping hits; of these 353 are in contigs and 2,364 in scaffolds. Small RNA sequencing libraries were made after a size selection step. Reads from each small RNA libraries were independently aligned to the assembly v.10 using Bowtie2 [41]. Resulting mappings and de-novo predicted small RNAs were used as an input to htseq-count, HTSeq v.0.6.1[42] to quantify small RNA features. We checked for the presence of sequences similar to rRNA by using the riboPicker tool [43]. As shown on Figure S5, the majority of the small RNAs are microRNAs, tRNAs and snoRNAs. In the Table S22 we have summarized number of the small RNAs annotated in the *A.thaliana* and legume plants in the Rfam database.

5. Prediction of plant disease resistance genes

An automated pipeline, the Disease Resistance Analysis and Gene Ontology (DRAGO) pipeline [44], was used to predict resistance genes (R-genes). This pipeline combines an homology search against a set of curated R-genes having experimental verification, followed by the search of the canonical domain combinations conferring the disease resistance function. The search for sequence homology consists of running BLASTP (with an e-value cutoff of 1e-10) against a manually curated set of 112 R-genes and performing the domain analysis using InterProScan v.5. Finally, proteins are assigned to each of the particular resistance classes depending on the domain combination they have.

The high number of predictions belonging to the RLK and RLP transmembrane classes indicates some type of overprediction by our pipeline. This can be due to the fact that both classes contain a general Kinase domain that is not specific for the disease resistance activity. Additionally, PTO-like R-genes were not predicted by our automated pipeline because this class contains only a general kinase domain. This class was initially identified in the tomato and interacts with the avrPto gene product of the bacterial pathogen *Pseudomonas syringae* and launches a disease sensitive response. In order to identify the PTO-like genes we used the available experimental evidence supporting the presence of this PTO-like

family in Bean by doing a BLASTP (e-value cutoff 1e-10) search of the proteins identified in the cited experimental work against our set of proteins.

Additionally, 120 R-genes were identified following a similar protocol by Schmutz et al [46]. Briefly, InterProScan v.5 results were scanned to identify sequences containing the NB-ARC domain (PF00931). After filtering results by the "trusted cutoff" established by PFAM (e-value equivalent to 1e-5), 174 genes were identified as containing NBS domains. Additional domains such as TIR (PF01582) and LRR (PF00560, PF07723, PF07725, PF12799, PF13306, PF13516, PF13504 and PF13855) were identified for this set of R-genes applying the same cutoff. Finally, those genes which were not previously identified were added to the R-genome of *P. vulgaris* BAT93 (Figure S4, Table S21).

<u>Long non coding RNA analysis</u>

Homology based lncRNAs were predicted using the strategy reported in [45], taking *A. thaliana* lncRNA transcripts taken from [47] as templates. These were blasted against the bean assembly previously masked for interspersed repeats using RepeatMasker [20] and the hits were then used as anchor points to re-align the *A. thaliana* queries with surrounding genomic regions using exonerate as a split aligner. Gene models thus recovered were then filtered for their coverage of the original query (>70%) and their content in plant specific ancestral repeats (<20%) as well as proximity to protein coding genes (>1kb). Final conservation was estimated on T-Coffee [48] pairwise re-alignments between the query and its predicted spliced model (excluding introns).

Ab-intio lncRNA models were predicted using Cufflinks [25] to build transcript models on all RNA-Seq samples, processed one at a time. Models containing only one exon or overlapping with protein-coding genes were filtered out. We then used Cuffmerge (from the Cufflinks suite) to combine transcript models from all samples into a single set of consensus models. These were then filtered by excluding models having high ancestral repeat coverage (>20%), coding potential (CPC [49] with default settings), any overlap with GeneID [27] predicted ORFs onto the genome, or ORF covering more than 25% of their length. These cut-offs were selected so as to retain the trusted predictions of non-coding genes obtained by homology. The full set was further filtered by eliminating all transcripts having both an RPKM lower than 0.1 in any of the original 27 RNAseq libraries and no homology support in any of the 12 plant genomes considered here. Sets of overlapping transcripts (>=1nt) were clustered into gene models and further filtered by removing all genes containing one or more transcripts within 1kb of protein coding genes.

Phylogenomic profiling was carried out by applying the homology-based strategy described above using the complete set as queries and the 12 genomes as targets. The horizontal clustering (Figure 3, main text) was obtained by imposing the 12 species phylogenetic tree topology. Gene level conservation was

estimated by considering, within each bean gene, the transcript conserved in the largest number of species. LncRNA transcript expressions were obtained using the Flux Capacitor (http://flux.sammeth.net/capacitor.html). Analysis was then done at the gene level, summing up the expression of transcripts belonging to the same loci. We only kept for further analysis genes with an expression of at least 0.1 RPKM in at least one condition. Housekeeping lncRNA genes were defined as the overlap between genes expressed with at least 0.1 RPKM in each of the organs with the set of lncRNA genes conserved in at least 7 species (Figure S14).

Transcriptome analysis

1.Read mapping and quantification

Prior to mapping RNA-Seq reads were inspected for the presence of adapter sequences and trimmed if found; no other filtering was performed. Then reads from each sample were independently aligned to the reference *P. vulgaris* assembly v.10 using the gemtools RNA-Seq pipeline v.1.6.2 (https://github.com/gemtools/gemtools). This pipeline uses GEM as a mapper [24], which performs full paired-end, quality-aware alignment and does not require preliminary filtering by quality. At the same time GEM is split-aware, and finds gapped matches, that is, it can correctly align reads into the genome originating from exon junctions. Gemtools maps reads independently to the genome, transcriptome and de novo-created splice junctions and then merges results of this three mappings into a single output file. Additional filtering steps were performed allowing less than 2 mismatches and less than 10 multimappings. On average, 89±5% of the reads were mapped across samples, 69±10% of the reads mapping uniquely (Dataset S5).

We used the Flux Capacitor (http://flux.sammeth.net/capacitor.htm) v1.2.4 to quantify genes, transcripts, exons and splice junctions in each sample separately. Expression levels were given in Read Per Kilobase per Million mapped reads (RPKM)[50] and in read counts. We have calculated Spearman correlation coefficient for each pair of replicates and performed hierarhical clustering and PCA of the whole pool of samples. Technical replicates that could generate noise in the analysis was removed: FTL_V2.2, ST_V4b.1, MPWS_R9.1, AM_V4b.1 and AM_R5.2. For remaining replicates we have applied “non-parametric irreproducible discovery rate” criteria [51]. The npIDR values were calculated for each genomic element independently (genes, transcripts, exons and junctions). The elements are assumed to be reproducible if they had in both replicates >0 reads mapped and its npIDR score was <0.1. We consider a gene to be expressed if it has RPKM value >=1 in at least one sample. In total we found expression in 21,472 (70%) genes across samples, in average 16042±1117 per sample(Table S30; Figure S10, Datasets S5-S7). Ubiquitous expression in all samples was found for 9,881(32%) genes.

For the differential expression analysis and co-expression network construction we have normalized read counts into counts per million (CPM) [52].

The libraries were classified into organ groups by its phenotype: root, stem, leaf, flower, axial meristems, pods and seeds. Also we grouped the libraries by developmental stage of the plant - 5 vegetative stages: V0 (Germination), V1 (Emergence), V2 (Primary leaves), V3 (1st. trifoliate leaf), V4 (3rd. trifoliate leaf) and 5 reproductive stages: R5 (Preflowering), R6 (Flowering), R7 (Pod formation), R8 (Pod filling), R9 (Maturation) (see Table S8).

2.Housekeeping genes (ubiquitous expression)

Coefficient of variation(CV) value was used to identify putative housekeeping genes — we have calculated CV for each gene and have selected top 10% of the genes with lowest CV. In total, we assign 2811 genes into housekeeping category(Dataset S8).

Previously published data sets — 1000 soybean and 2500 common bean, variety Negro Jamapa(PvGEA), housekeeping gene set were downloaded from the supplementary materials of the corresponded papers — Severin et al., Additional file 8 [53] and O'Rourke et al., Additional file 22 [54]. Ortology one-2-one relationships from the phylomeDB was used to compare selected gene sets. Beside the orthologs we can identify 92% of PvGEA genes and 78% soybean genes. In common between three data sets were 195 genes; and 1279 genes were in common between PvGEA and our data sets (Figure S13).

3. Specific gene expression across organs and stages

Organ and stages specific expression was estimated with applying a threshhold of ≥0.1 RPKM to average gene expression in a given organ/stage, compare to the other organs/stages. GO terms enrichment analysis was done with R bioConductor package topGO using whole set of genes as a background.

In total we assigned 937 protein-coding and 171 long non-coding RNA genes to be organ specific. As stages-specific we identified 624 protein-coding and 130 long non-coding genes.(Table S30, Figures S14,S17)

4. Differential expression

Differential expression was estimated with the edgeR package (R version: 3.0.1, edgeR v. 3.2.4); using statistical methods based on generalized linear models (GLMs) [55, 56]. To estimate the common dispersion in the absence of biological replicates we choose pseudo-biological replicas: SP_R7 / MP_R9, NR_V3 / R_V3, AM_V4b / AM_R5 FTL_V2 / TL_V4b. The common dispersion calculated (0.12887) was use for the rest of the analysis of differential expression. Genes that exhibited an FDR ≤ 0.05 and a log2 Fold Change ≥ 1 (log2FC) were determined to have differential expression (Datasets S12-S22).

5. Co-expression network

Gene co-expression networks provide a high level view on the relations between the expression of individual genes and help to identify sets of gene sharing expression profiles across multiple conditions. In a co-expression network two genes are connected if their transcriptional profiles have significant correlation. For the construction of the bean co-expression network we used all protein-coding and long ncRNA genes that have >3CPM at least in one sample. From this set, we removed outlier genes that capture ~30% of expression in three tissues (Figure S12) since they could introduce noise into network; 21,560 genes remained. The resulting data matrix was standardized both along genes (rows) and samples (columns). Standardization guarantees that only the profile and not the magnitude of gene expression contribute to covariance estimation. Standardization was done with the R-function 'scale'. The Pearson correlation matrix was calculated from the scaled data, and graphical lasso [57] with penalty parameter RHO =0.9 was applied to this matrix to reconstruct the network. The resulting network has 8,884 genes (vertex) connected with one or more edges (max is 260 connections per one vertex, mean is 18; density 0.002; transitivity 0.45) and 12,676 singletons. Downstream analyses were performed on the sub-network with vertex having one or more edges.

A random network with the same node degree distribution was generated using erdos.renyi.game command from the igraph R package. The resulting network had a maximum of 35±3 connections, and a density and transitivity 0.002.
A Fast-greedy community algorithm [58] was used to divide network into the modules. Modules are the set of nodes with a higher number of connections within module members than between members from different modules In total 738 modules were identified with 2-1271 genes in each module; 11 modules had more than 100 genes (Figure 5, main text).

The GO terms enrichment analysis with the R package topGO was performed for the 11 largest modules (FDR ≤ 0.05, Datasets S23,S24).

6. Analysis of duplicated and paralogous genes expression.

For each pair of duplicated genes we have calculated pearson correlation coefficient and TEC score as indicator of their expression similarity.

Pearson correlation coefficient was calculated on normalized RPKM values across all samples.
To compute tissue expression complementarity (TEC) score we have transformed vector of the expression values among all samples for a given gene into set composed of 1 and 0; where 1 or 0 is corresponded to the expression above or below threshold of selected gene in a given sample (presence/absence of expression). Here we used 1RPKM as a threshold.

TEC score for two genes *i* and *j* was calculated by the formula suggested by the Huerta-Cepas et al. [59]:
$TEC_{ij}=(d_i/t_i + d_j/t_j)/2$, where d_i is the number of samples in which gene i is specifically expressed (i.e. gene j is not expressed) and t_i is the total number of samples in which gene i is expressed.

For each group of paralogous genes we have calculated its coefficient of variation (CV) as standard deviation normalized by mean expression level of the gene, for the genes having ≥ 1 RPKM in at least one sample. The CV value was used as a level of fluctuation of the gene expression across all samples. In additional we have performed few rounds of simulations in which genes were assigned to paralogous groups randomly (Figure 6C, main text).

7. Publicly available transcriptomic data

RNAseq data from the Bellucci et al [60] and Rourke et al [54] studies were downloaded from the SRA archive [61]. The reads from each sample were independently aligned to the *P. vulgaris* assembly by using the gemtools RNAseq pipeline and gene expression was quantified by using Flux Capacitor, as was described earlier in the section 1. On average 91±2.8% / 80.7±7.4% reads were mapped, and 73.1±15.5%/ 77.6±7% reads were uniquely mapped for the Bellucci and Rourke data sets, respectively. In total across our and these additional samples, at a threshold of RPKM>1 we detected expression in 22,682 genes (74%), in average 16,150±1041 per sample,. In order to reduce non-biological variability between samples originated from the different studies, we have corrected for the batch effect with the R package 'combat'.
Hierarchical clustering analysis of the expression profiles were performed using the hclust command in R and default complete linkage method (Supplementary figure S9)

Phylogenetic analysis

1. Phylome reconstruction

Proteins encoded in 14 fully-sequenced plant genomes, including the two common bean genomes BAT93 and G19833, were downloaded from various sources (Table S24). Three different phylomes were reconstructed following the same procedure. The first phylome only contains sequences from the BAT93 accession. This phylome contained 30,405 BAT93 unique longest transcripts which led to reconstruct 27,986 single-gene trees. The second and third phylomes contain sequences from both bean accessions. 30,405 BAT93 and 27,126 G19833 unique longest transcripts were used for reconstructing those phylomes. The resulting phylomes yielded 28,075 and 26,304 single-gene trees, respectively.

In order to perform the phylome reconstruction, a Smith-Waterman [62] search was used to retrieve homologs using an e-value cut-off of 1e-5, and considering only sequences that aligned with a continuous region representing more than 50% of the query sequence. Then, selected homologous sequences were aligned using three different programs: MUSCLE v3.8 [63], MAFFT v6.712b [64], and KAlign v2.08 [65]. Alignments were performed in forward and reverse direction (i.e. using the Head or Tail approach [66], and the six resulting alignments were combined using M-Coffee [67]. The resulting combined alignment was subsequently trimmed with trimAl v1.4 [68], using a consistency score cutoff of 0.1667 and a gap score cutoff of 0.1, to remove poorly aligned regions.

Phylogenetic trees based on the Maximum Likelihood (ML) approach were inferred from these alignments. ML trees were reconstructed using the two best-fitting evolutionary models. The selection of the evolutionary models best fitting each protein family was performed as follows: A phylogenetic tree was reconstructed using a Neighbour Joining (NJ) approach as implemented in BioNJ [69]. The likelihood of this topology was computed, allowing branch-length optimization, using nine different models (JTT, LG, WAG, Blosum62, MtREV, CpREV, VT, DCMut and Dayhoff), as implemented in PhyML v3 [70]. The two evolutionary models best fitting the data were determined by comparing the likelihood of the used models according to the AIC criterion [71]. Then, the two ML trees were derived using these models with the default tree topology search method NNI (Nearest Neighbor Interchange). A similar approach based on NJ topologies to select the best-fitting model for a subsequent ML analysis has been shown previously to be highly accurate. Branch support was computed using an aLRT (approximate likelihood ratio test) parametric test based on a chi-square distribution, as implemented in PhyML. In all cases, a discrete gamma-distribution with four rate categories plus invariant positions was used, estimating the gamma parameter and the fraction of invariant positions from the data.

2. Orthology/paralogy predictions

Orthology and paralogy relationships among *P. vulgaris* genes and those encoded by the other considered genomes were inferred using a phylogenetic approach [72] (Tables S25 and S26). In brief, a species-overlap algorithm, as implemented in ETE v2 [73], was used to label each node in the phylogenetic tree as duplication or speciation depending on whether the descendant partitions have, at least one, common species or not (i.e. using a Species Overlap Score of 0). The resulting orthology and paralogy predictions can be accessed through phylomeDB.org [35]. These predictions have been used in subsequent analyses such as orthology-based functional annotation, identification of gene expansions, or duplication dating.

3. Functional gene annotation using orthology.

To complement the functional annotation based on proteins signatures, we functionally annotated BAT93 genes using orthology relationships. 13,522 one-to-one orthology relationships among *P. vulgaris* BAT93 genes and genes from other 5 species used in this project were found. Using these pairs, 43,028 GO terms were transferred from *P. vulgaris* BAT93 counterparts genes to them. Taking into account the annotation based in proteins signatures, we were able to add 20,696 new GO terms to 5,211 genes of which 365 genes were functionally annotated for the first time.

4. Gene mapping between *P. vulgaris* BAT93 and G19833 accessions.

Phylomes containing both bean accessions were used to assess to what extend the gene-set predicted for *P. vulgaris* BAT93 overlapped with the one for *P. vulgaris* G19833. 21,600 (~69.8%) genes in BAT93 were mapped to 21,604 (~79.5%) ones in G19833 (Table S18). Single gene-trees from both phylomes were scanned to predict one-to-one orthology relationships among bean sequences. Reciprocal one-to-one predictions constitute the biggest proportion of the mapping (~93.9%). One-to-one predictions detected from only one accession constitute the second source of mapping. Protein sequences were also scanned looking for identical sequences. Cases without orthology predictions but with identical sequence in both genomes were included as part of the mapping. Gene mapping was complemented by analyzing the gene-order conservation between bean accessions. Genes mapping to the same pseudo-chromosome/linkage group surrounded by genes already mapped were added to the final set.

5. Conservation level of orthologous PCGs of P. vulgaris BAT93 and G19833 acessions.

We aligned all orthologous pairs to assess their conservation in terms of sequence identity using MAFFT (parameter --auto) [64]. Identity was estimated using trimAl [68] before and after removing columns with gaps (parameter -nogaps). Orthologous pairs with sequence identities up to 0.95 were grouped into

different bins (Supplementary table S19). Enrichment analyses for over-represented GO terms in these proteins compared with the whole set of annotated P. vulgaris BAT93 and G19833 PCGs was performed using FatiGO as implemented in the Babelomics webserver [74]. A Fisher exact test looking for overrepresented terms in specific sets of proteins against the whole annotated genome was used with a e-value cutoff of 0.001. Supplementary table S20 contains enriched GO terms at different sequence identity thresholds.

6. Species tree reconstruction

A phylogeny for the species included in the phylome was inferred using two complementary approaches, which rendered identical topologies. Firstly, a super-tree was inferred from all the trees in the three phylomes (82,365 trees) by using a Gene Tree Parsimony approach as implemented in the dup-tree algorithm. This procedure finds the species topology which minimizes the number of total duplications implied by a collection of gene family trees, i.e. the phylomes. Secondly, 172 gene families with a clear, phylogeny-based, one-to-one orthology present in the 14 species included in the analyses, were used to perform a multi-gene phylogenetic analysis. Protein sequence alignments were performed as described above and then concatenated into a single alignment. Species relationships were inferred from this alignment using a Maximum Likelihood (ML) approach as implemented in PhyML [70], using JTT as evolutionary model, since in 153 out of 172 gene families this model was the best-fitting. Branch supports were computed using an aLRT (approximate likelihood ratio test) parametric test based on a chi-square distribution.

7. Comparative genomics, in terms of homologous relationships, among plant species

A comparative analysis, in terms of homology relationships, was performed among the 14 species included in two of the three phylomes. To carry out such analysis, a BLAST search of all species against all species was performed to retrieve homologous sequences with a cut-off e-value of 1e-5 and a minimum coverage of 50% between query and target sequences. Then, results showing different patterns of homology, i.e. genes present in all species or specifically restricted to legumes, were computed and plotted in the species tree inferred (see Figure 3, main text).

8. Legume-specific proteins detected in *P. vulgaris* BAT93

P. vulgaris BAT93 proteins with detectable homologous sequences at legume species level were analyzed looking for any functional enrichment. Two patterns of homology were selected: 1) proteins with homologs in all 5 legumes species, and 2) proteins with homologs in at least one of the legume species included in this study but not BAT93. Enrichment analyses for over-represented GO terms for

legume-specific proteins as compared with the whole set of annotated *P. vulgaris* BAT93 proteins were performed using FatiGO as implemented in Babelomics webserver [74]. A Fisher exact test looking for overrepresented terms in specific sets of proteins against the whole annotated genome was used with e-value cutoff of 0.001. Table S27 shows over-represented terms grouped by homology profiles and ontologies.

9. Accession specific expansions in *P. vulgaris* BAT93

We focused on accession specific expansions of the BAT93 genome for which 4,163 genes (~13.65%) were mapped to such events. Then, these genes were grouped into clusters with at least 75% of shared genes and a minimum size of 10 members. 974 genes (~23%) were assigned to an unique cluster. A broader selection of genes were filtered out because either they mapped to lineage-specific expansion in *P. vulgaris* or at least 25% of the cluster members had homologs in the G19833 genome. Fig. S6 shows the clusters distribution in terms of clusters size.

Those clusters were analysed looking for any statistically significant functional enrichment. Functional enrichment is provided for all clusters with at least one significant enriched term. Enrichment analyses of over-represented GO terms were performed by using FatiGo as implemented in Babelomics webserver [74] using the Fisher exact test for genome comparison and e-value cutoff of 0.001. Then, GO terms redundancy was reduced using REViGO webserver [75] setting a similarity threshold of 0.7, using the ratio of odd log as metric, as reference database the annotation the whole UniProt and as semantic similarity algorithm SimRel. Dataset S2, Additional file 2 contains the functionally enriched terms grouped by clusters.

10. Lineage specific expansions in *P. vulgaris*

We scanned single-gene trees from phylomes containing both bean genomes to detect genes family expansions predating the split of both accessions. We detected 661 BAT93 and 800 G19833 genes mapping to such events. Then, those genes were grouped into clusters with at least 75% of shared genes and a minimum size of 10 genes. 547 genes (~37%) were assigned to an unique cluster. Figure S7 shows the distribution of those clusters in terms of clusters size.

These clusters were analyzed looking for any statistically significant functional enrichment. We put together the functional annotation available for both genomes in order to carry out this analysis. Functional enrichment is provided for all clusters with at least one significant enriched term. Enrichment analyses of over-represented GO terms were performed by using FatiGo as implemented in Babelomics webserver using the Fisher exact test for genome comparison and e-value cutoff of 0.001. Then, GO terms

redundancy was reduced using REViGO webserver [75] setting a similarity threshold of 0.7, using the ratio of odd log as metric, as reference the whole UniProt database and as semantic similarity algorithm SimRel. Dataset S3 contains the functionally enriched terms grouped by clusters. There is a label to indicate which phylome was used to detect such cluster. Identical clusters (11) were labeled as *phylome_BAT93*.

11. Dating of duplications for *P. vulgaris* BAT93

We scanned the BAT93 phylome to detect and date duplication events, using a previously-described algorithm [76]. We focused on events assigned to four different relative evolutionary periods: basal to *P. vulgaris*, basal to legumes, basal to rosids, and basal to the split of rosids and asterids. Individual trees were scanned and all duplication events that involved the seed protein and others bean proteins were dated. All these pairs together with their relative age were used to analyze the correlation, if any, between the age of the events and the expression patterns among paralogous proteins. Additionally, we recorded for each seed protein how many paralogous sequences were found. This information will be used to detect variations in terms of expression regarding the number of detected paralogous.

The ratio given by the number of detected events per age divided by the total number of single-gene trees reconstructed in the phylome informs about massive gene duplications, including Whole Genome Duplication (WGD) events, at specific relative ages in evolution. As shown in Table S28, it is possible to trace back the ancestral polyploidization events predating the split of rosids and asterids [77] where proteins almost duplicated twice. However, we did not detect similar duplication patterns at any of the other 3 relative ages. This can be easily understood taking into account the duplications patterns shown in Figure S8 where two clear patterns are present in terms of 1) number of duplicated proteins, and 2) how many times each protein got duplicated at each relative age.

P. vulgaris BAT93 proteins duplicated at different relative ages were analyzed looking for any functional enrichment. Enrichment analyses for over-represented GO terms were performed using FatiGO as implemented in Babelomics webserver [74]. A Fisher exact test looking for overrepresented terms in specific sets of proteins against the whole annotated genome was used with an e-value cutoff of 0.001. Then, redundancy was reduced using REViGO webserver [75] setting a similarity threshold of 0.7, using the ratio of odd log as metric, as reference database the whole UniProt database and as semantic similarity algorithm SimRel. Dataset S4 contains the resulting functionally enriched terms grouped by relative age.

12. Assigning relative ages to *P. vulgaris* BAT93 proteins.

We scanned the BAT93 phylome to date speciation events, using a previously-described algorithm [76]. We only considered the furthest orthologous sequence, in terms of relative ages, for each seed protein. In this way, we were able to date 24,098 genes (~79%) of the BAT93 genome. For the remaining genes, we analyzed the BLAST output, after removing the limit of 150 sequences, to detect the most distant homologous sequence and date those genes according to the relative age of those homologs.

Supplementary Figures

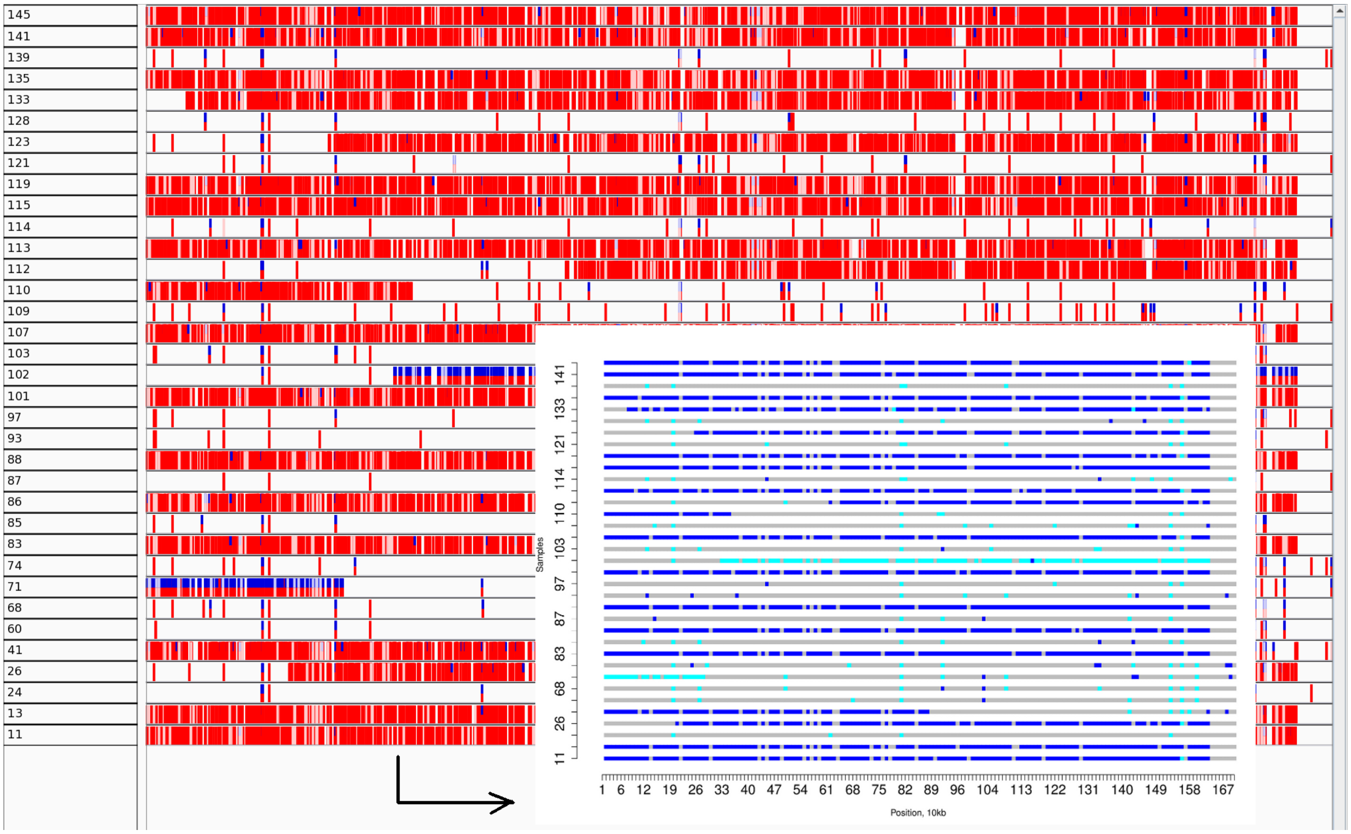

Figure S1. Genoype By Sequencing (GBS) profile. Profile for the scaffold00009 based on the Single Nucleotide Variants (SNVs) between progeny samples and the reference assembly. In the top part — variants profile view in the integrative genomics viewer (IGV) [78] and in the bottom part — profile view in the 10kb windows (in house script).

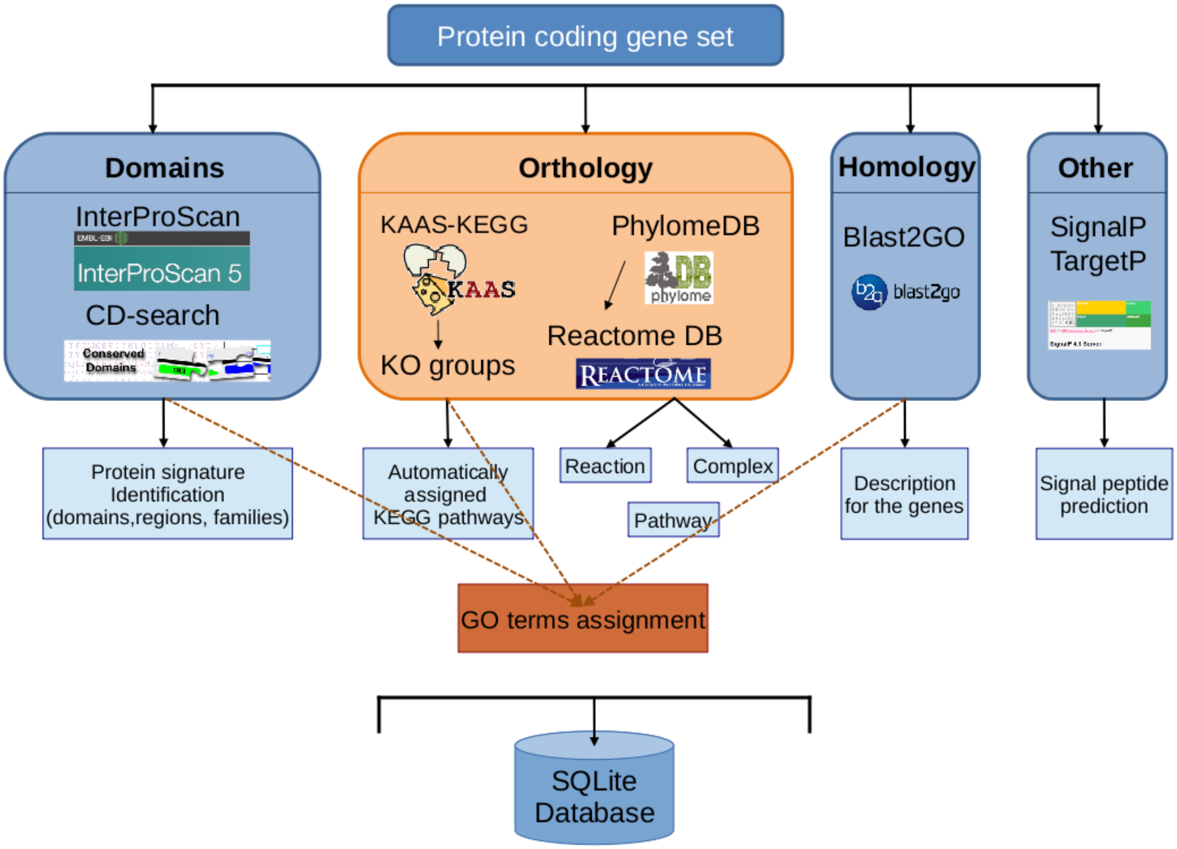

Figure S2. Functional annotation. Schematic diagram of the functional annotation workflow.

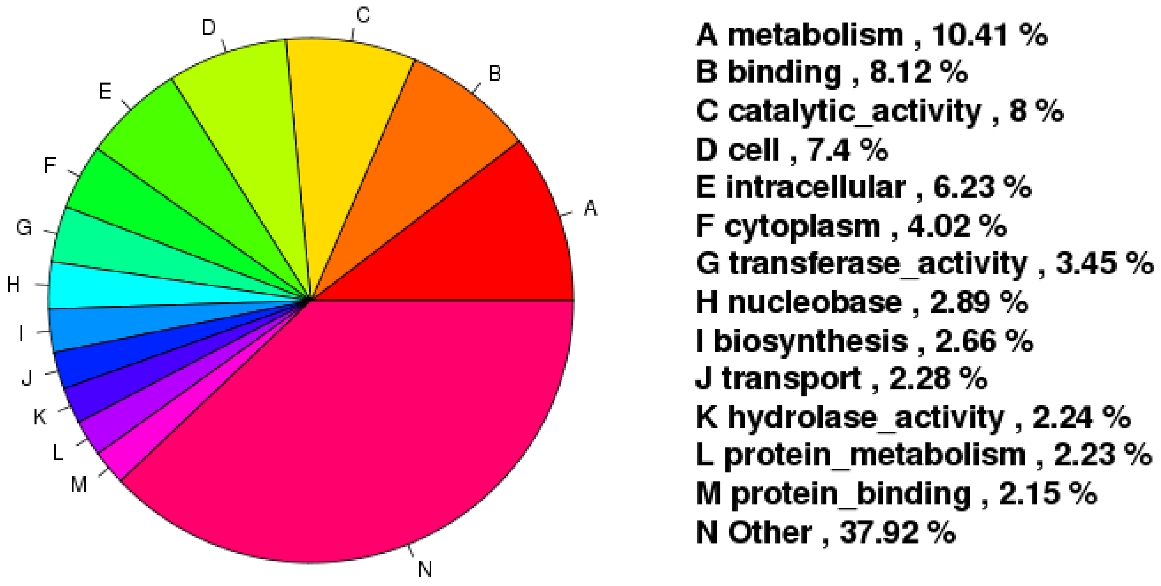

Figure S3. Gene ontology mapping of *P. vulgaris* genes. The GO terms were mapped into plant GO slims without top level categories – biological process, molecular function, cellular component.

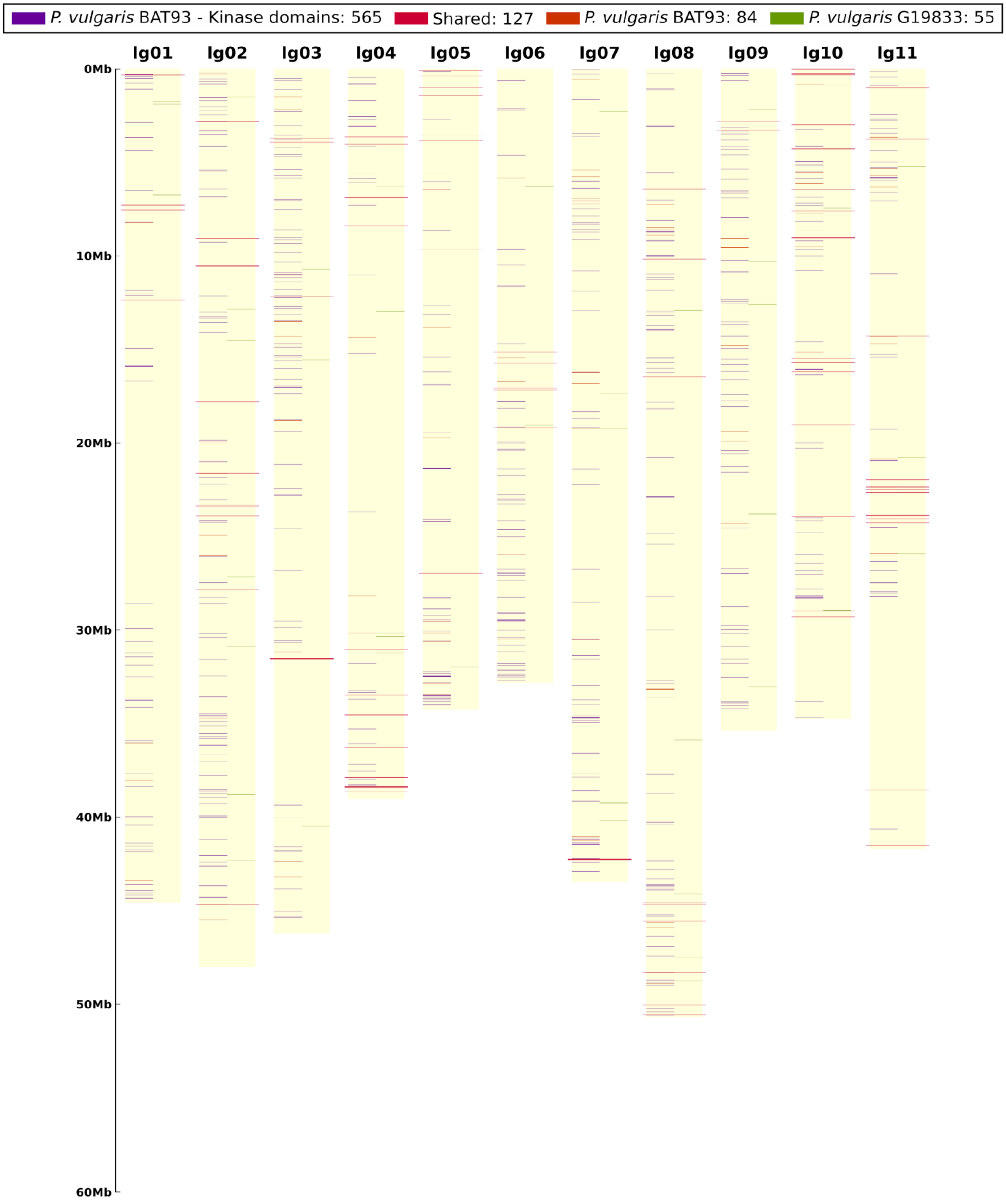

Figure S4. Resistance genes (R-genes) mapped to the linkage groups. R-genes detected for BAT93 and G19833 mapped to the linkage groups of BAT93. R-genes specific to BAT93 are located at the left side of

the vertical bars representing the linkage groups whereas those R-genes specific to G19833 are located at the right side. R-genes shared by both genomes are represented by lines entirely crossing the bars. R-genes containing at least one kinase domain are represented as a different category since those genes were not predicted for the G19833 genome. Given the repetitive nature of R-genes, G19833 genes were mapped into BAT93 genes using bidirectional BLAST hits rather than the more accurate and finer methods based on orthology predictions.

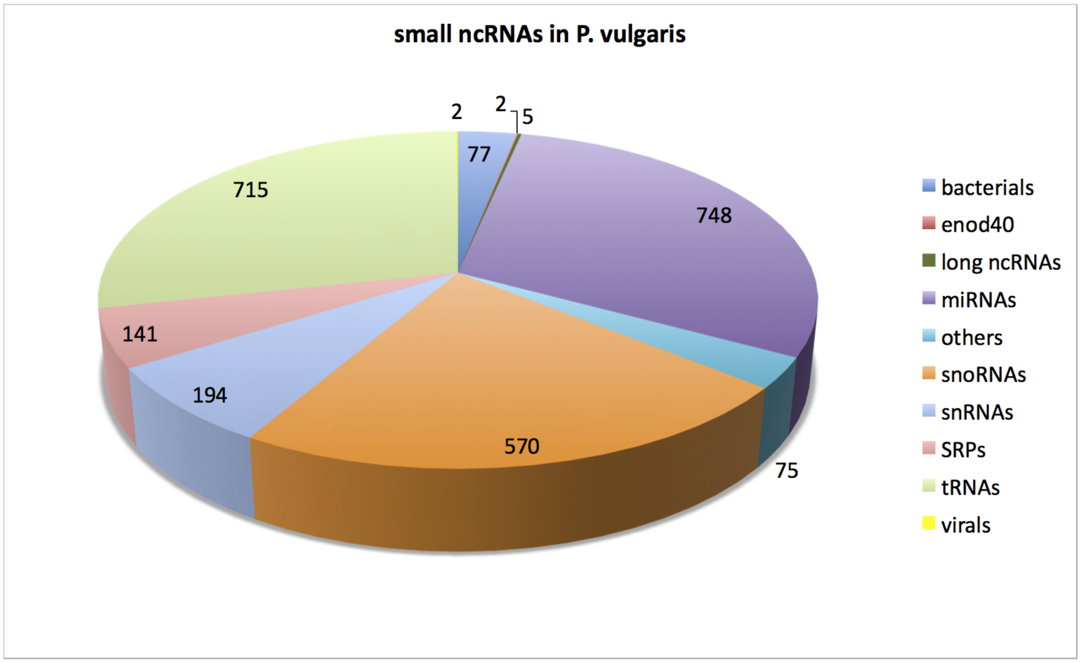

Figure S5. Small RNAs classification in *P. vulgaris*.

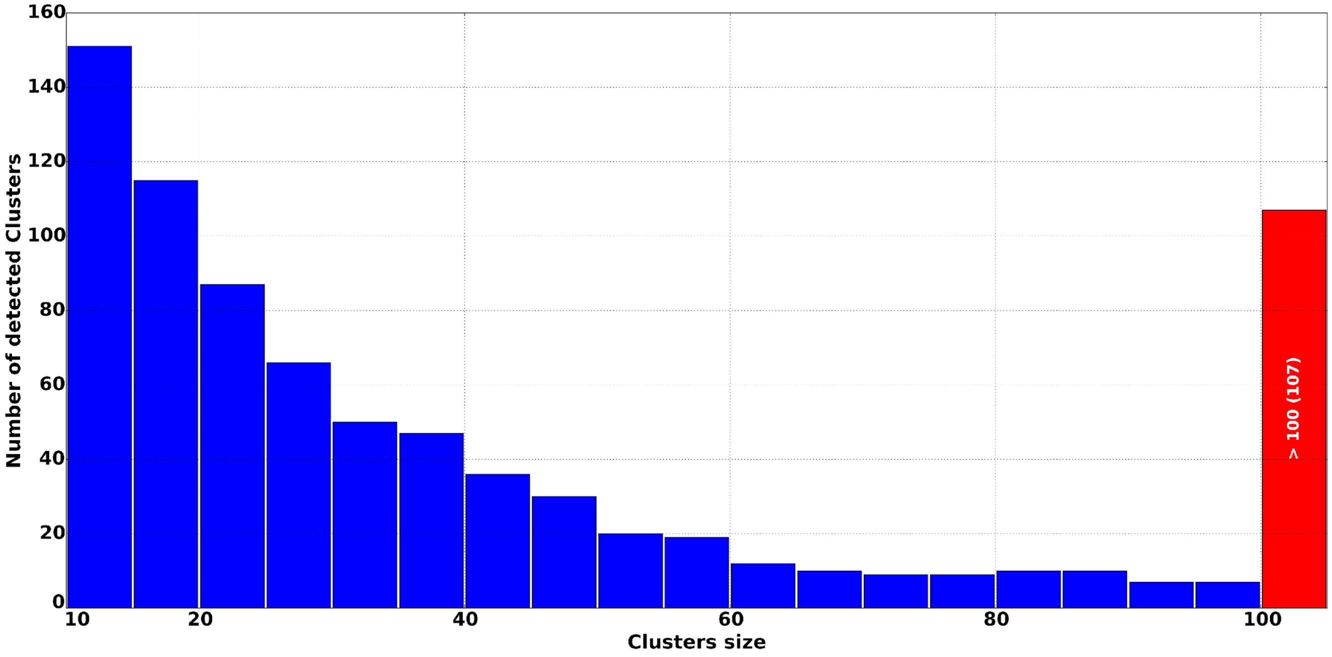

Figure S6. Clusters of specifically expanded proteins in BAT93 genome. Frequency and size of the different clusters of accession specific expanded family genes. On the plot, only clusters grouping 10 or more proteins were considered.

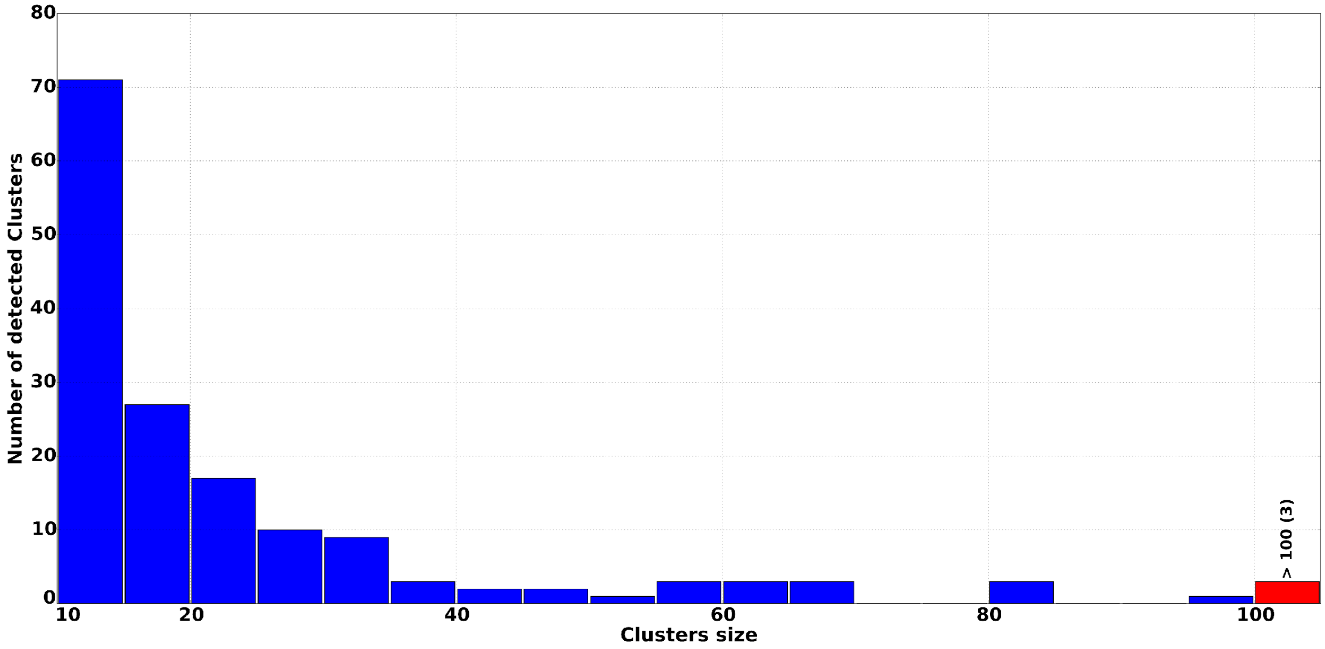

Figure S7. Clusters of specifically expanded proteins in the *P. vulgaris* lineage. Frequency and size of the different clusters of lineage-specific expanded family genes. On the plot, only clusters grouping 10 or more proteins were considered.

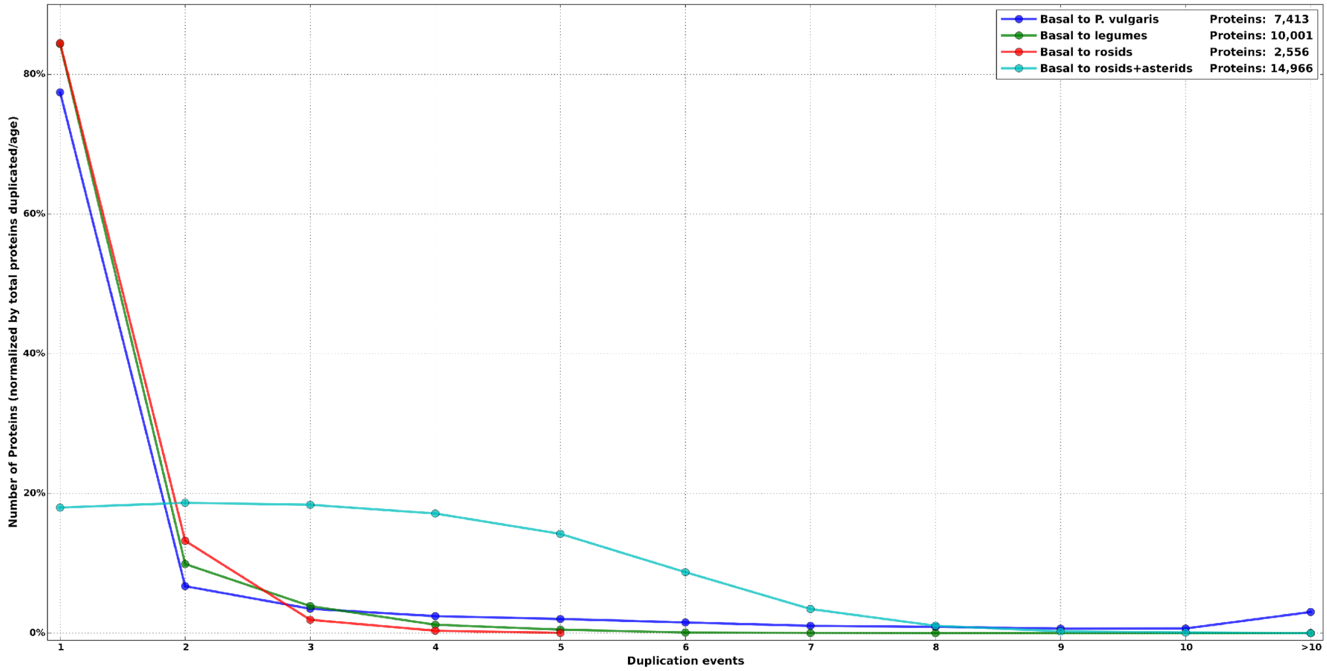

Figure S8. Duplication patterns at different evolutionary distances. Number of duplications events, by relative evolutionary ages, detected for BAT93 proteins. Only up to 10 events are shown in this plot.

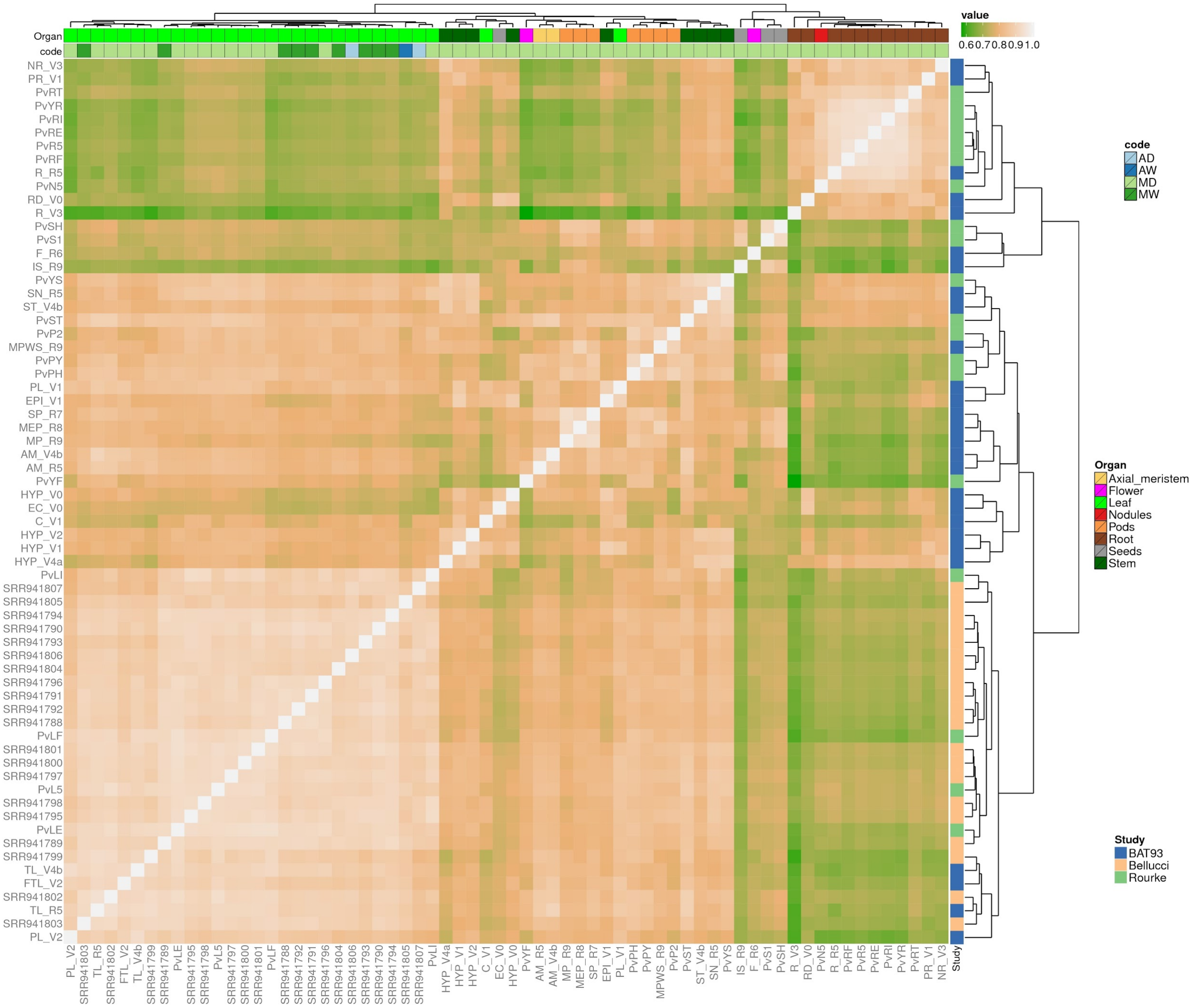

Figure S9. Hierarchical clustering of bean samples. The clustering is based on protein coding gene expression. The transcriptomic samples used include, in addition to those from BAT93 obtained here, samples from Bellucci et al [60] on a variety of Mesoamerican and Andean domesticated and wild accessions and O'Rourke et al[54] on Mesoamerican accession Negro Gamapa (right color panel)**.** The colors in the upper panel is the population code as it was defined in the Bellucci et al.: Mesoamerican domesticated (MD), Mesoamerican wild (MW), Andean domesticated(AD) and Andean wild (AW).

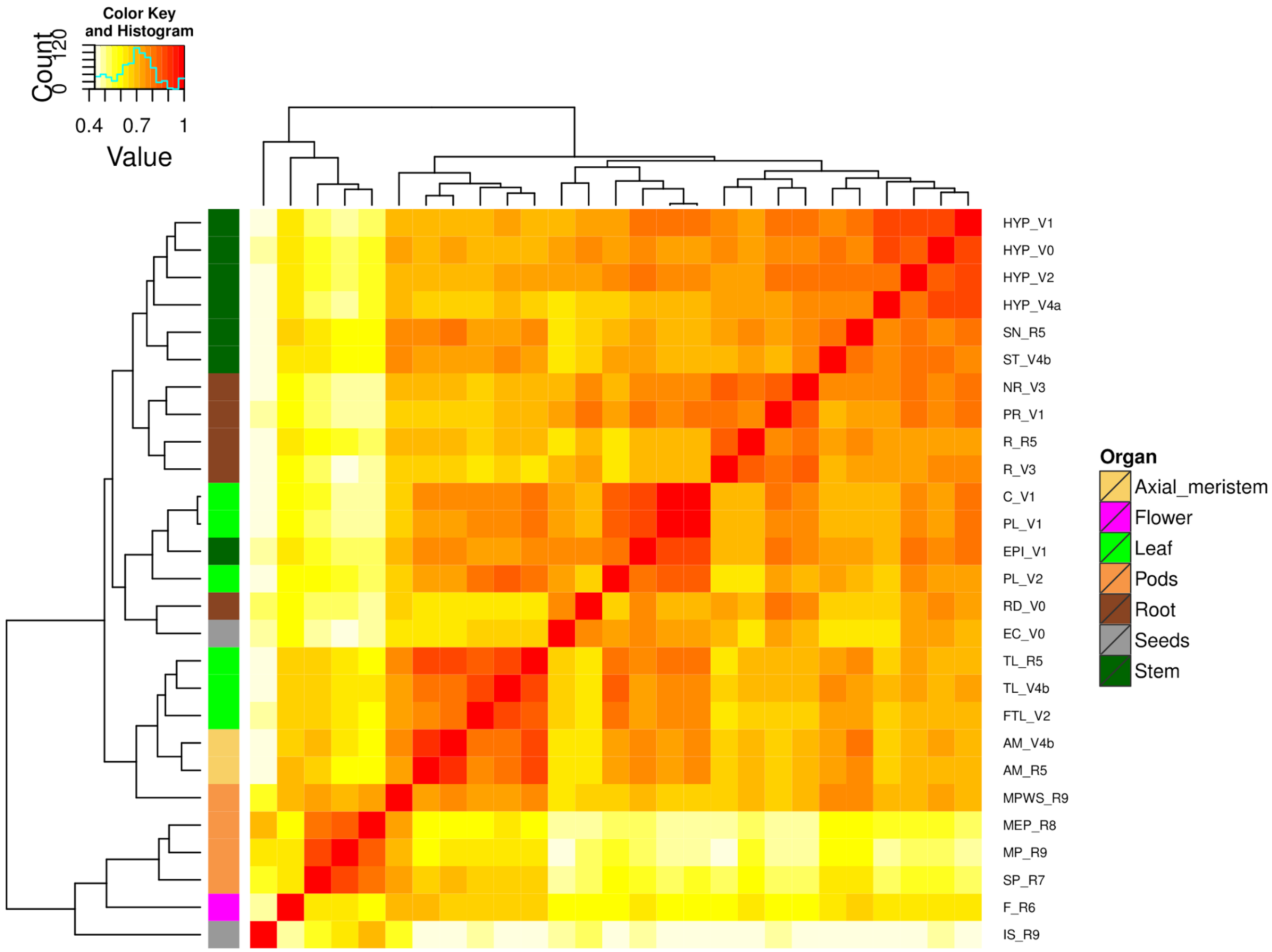

Figure S10. Hierarchical clustering of the BAT93 samples based on the expression of lncRNA genes.

A.

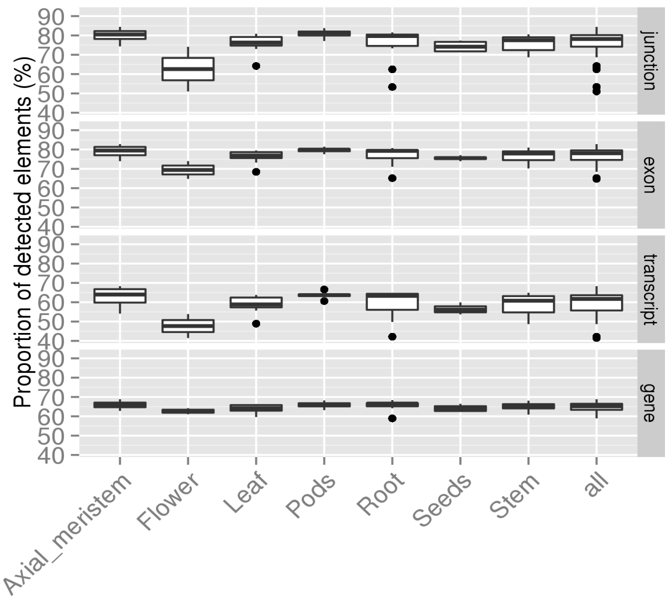

B.

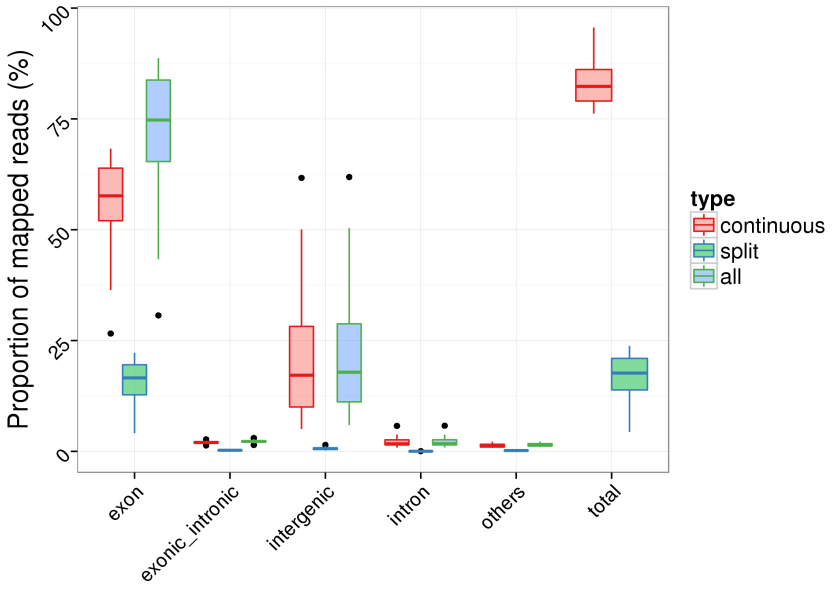

Figure S11. Detected genomic elements. A. Statistics on detected genomics elements for PCGs in all samples, and separately in samples belongs to each organ group. Each box plot is generated from values across all samples, thus capturing dispersion across different samples. The dots shows the cumulative value over all samples. **B.** Proportion of RNASeq reads mapping to the main genomic domains (exons, introns and intergenic regions)

A.

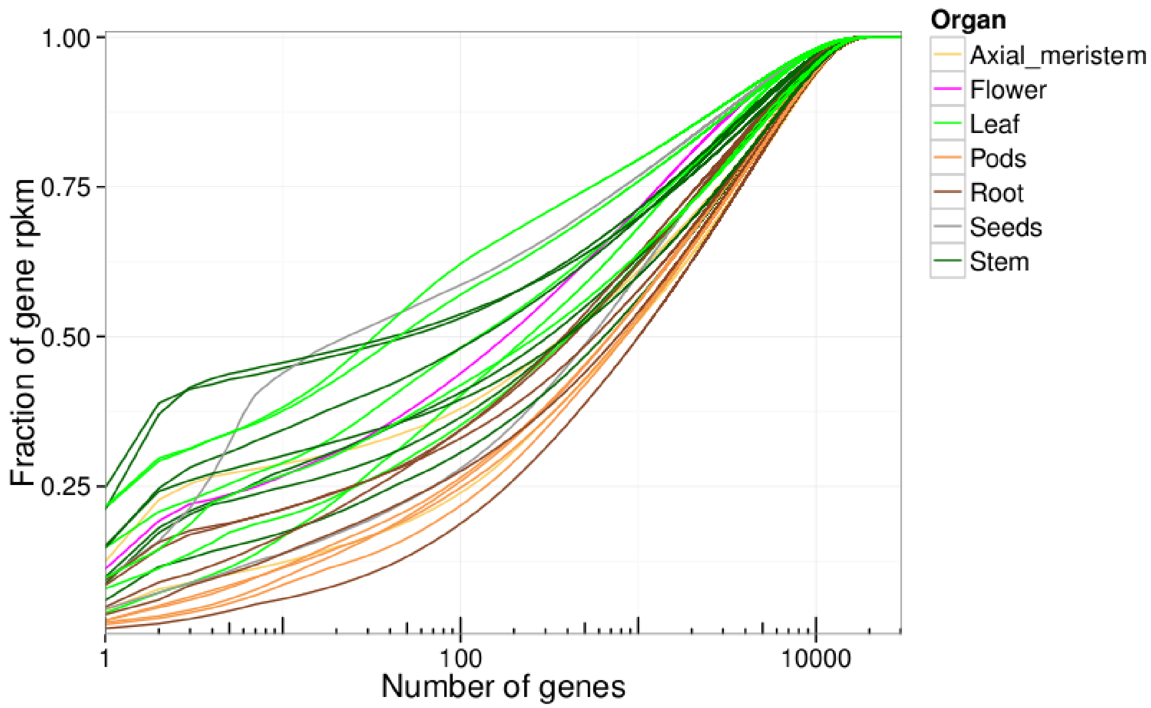

B.

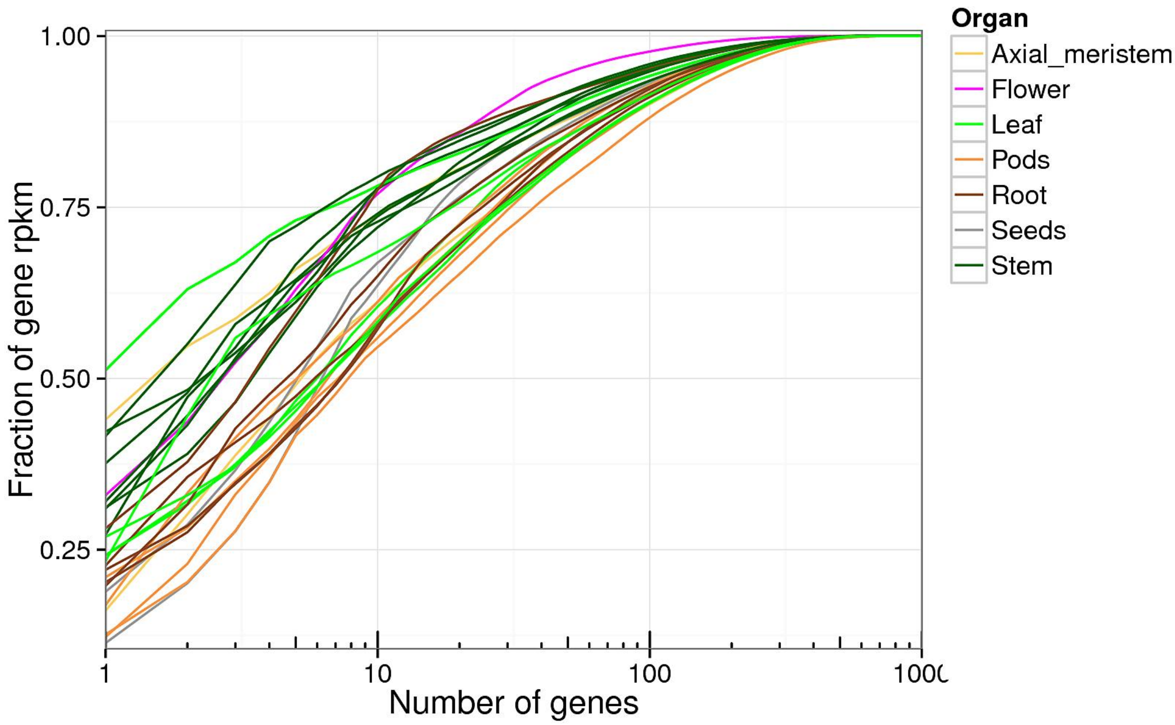

Figure S12. Cumulative gene expression distribution. Cumulative distribution of the average fraction of total protein coding gene/ lncRNA transcription contributed by genes when sorted from most-to-least

expressed in each tissue (x axis). Lines represent mean values across samples of the same organ. **A. Protein coding genes.** The gene that captures about 30% of the protein coding transritption in the Stem samples (HYP_V4a, SN_R5) is annotated as putative senescence-associated protein (PHASIBEAM10F000344), and the six genes that capture 50% of transritption in seeds (IS_R9) are annotated as phaseolins (PHASIBEAM10F000091, PHASIBEAM10F000196, PHASIBEAM10F000220, PHASIBEAM10F000387, PHASIBEAM10F024660, PHASIBEAM10F026545) **B. Long non-coding RNAs.** The gene that captures more than 50% of lncRNA expression in leafs (PL_V1) is XLOC_002036.

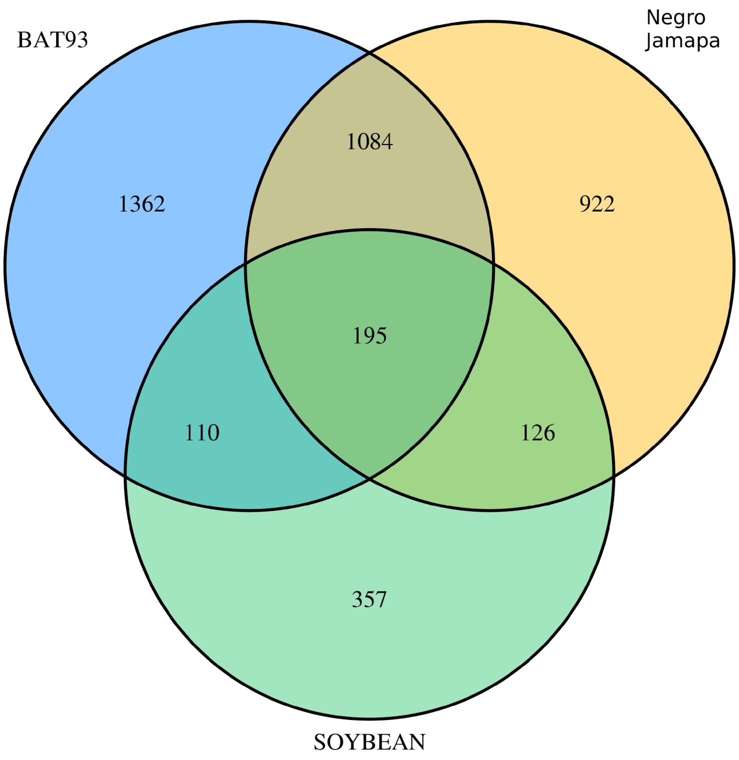

Figure S13. Venn diagram of the overlap between three sets of putative plant housekeeping genes. Housekeeping genes were obtained from this study (*P.vulgaris* BAT93) variety, from O'Rourke et al [54] (*P.vulgaris* variety Negro Jamapa) and from Severin et al. [53] (*Glycine max*.)

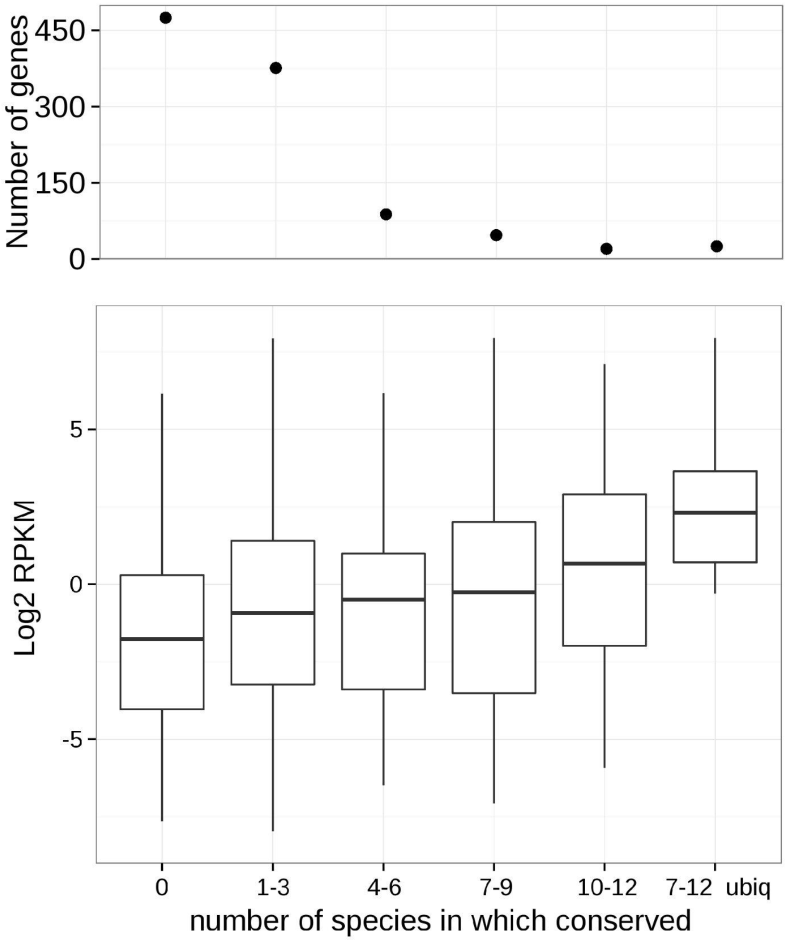

Figure S14. Expression of lncRNAs. Boxplots of average gene expression in lncRNAs. The vertical axis indicates average log RPKMs across tissues: genes are binned according to the number of species in which they have homologues. In the top panel the number of genes in each bin are given. The rightmost box depicts the core set of 25 lncRNAs that are ubiquitously expressed in all organs and conserved in at least 7 species.

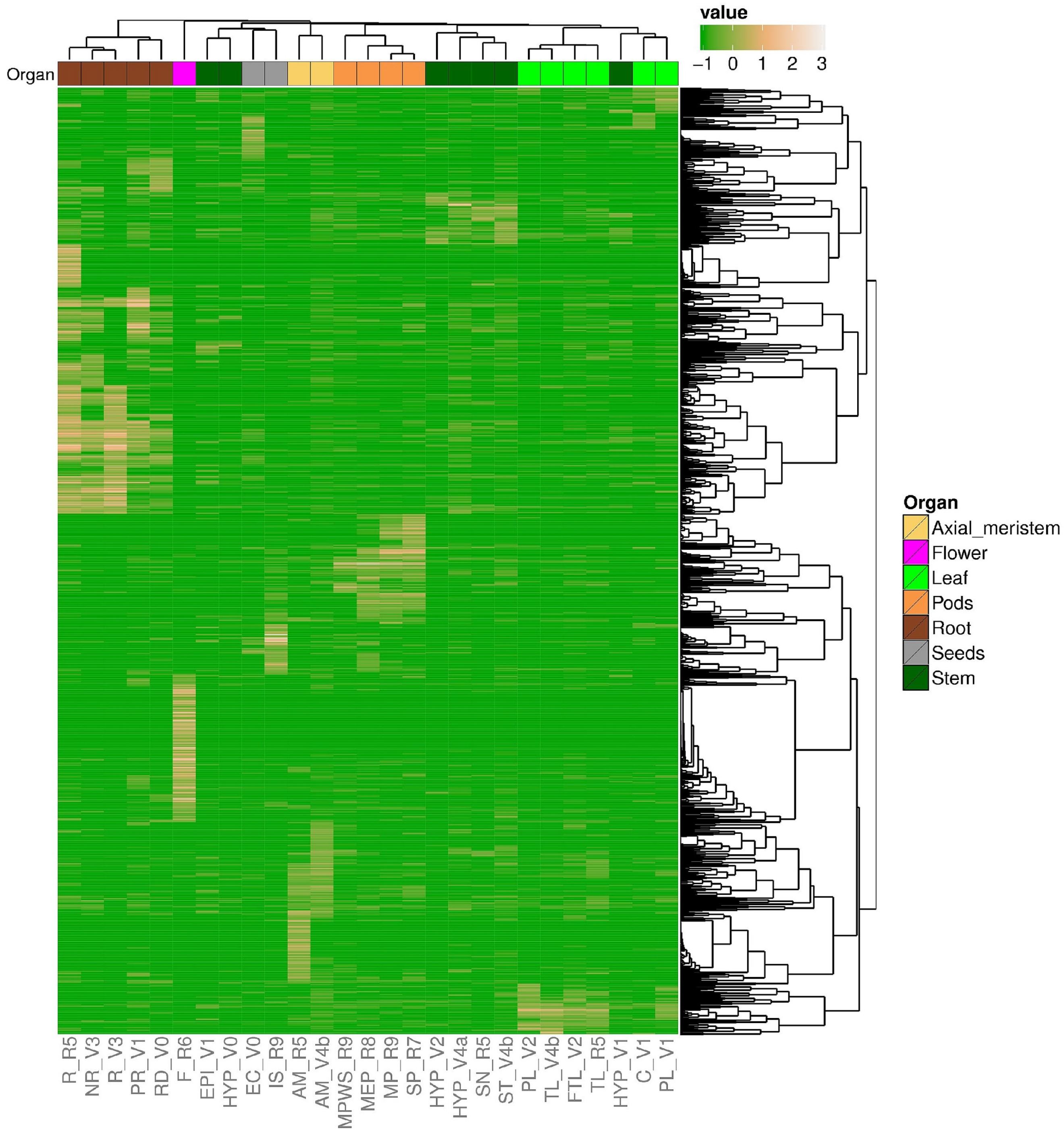

Figure S15. Organ-specific gene expression. Heat-map of log10(RPKM) values for genes specifically expressed only in one organ. Here we used 0.1 as a pseudo counts for the log transformation.

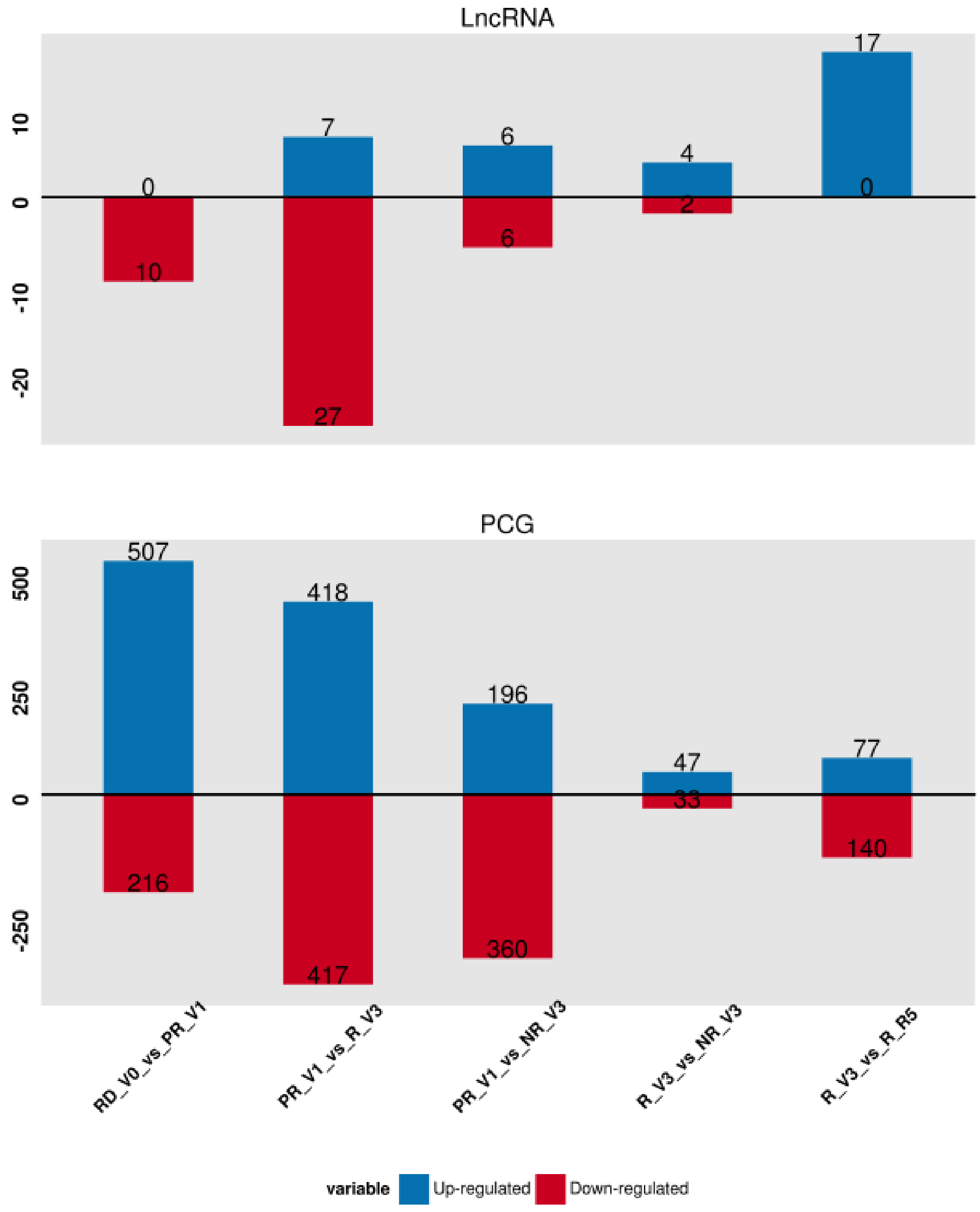

A. Root samples transcriptome dynamics

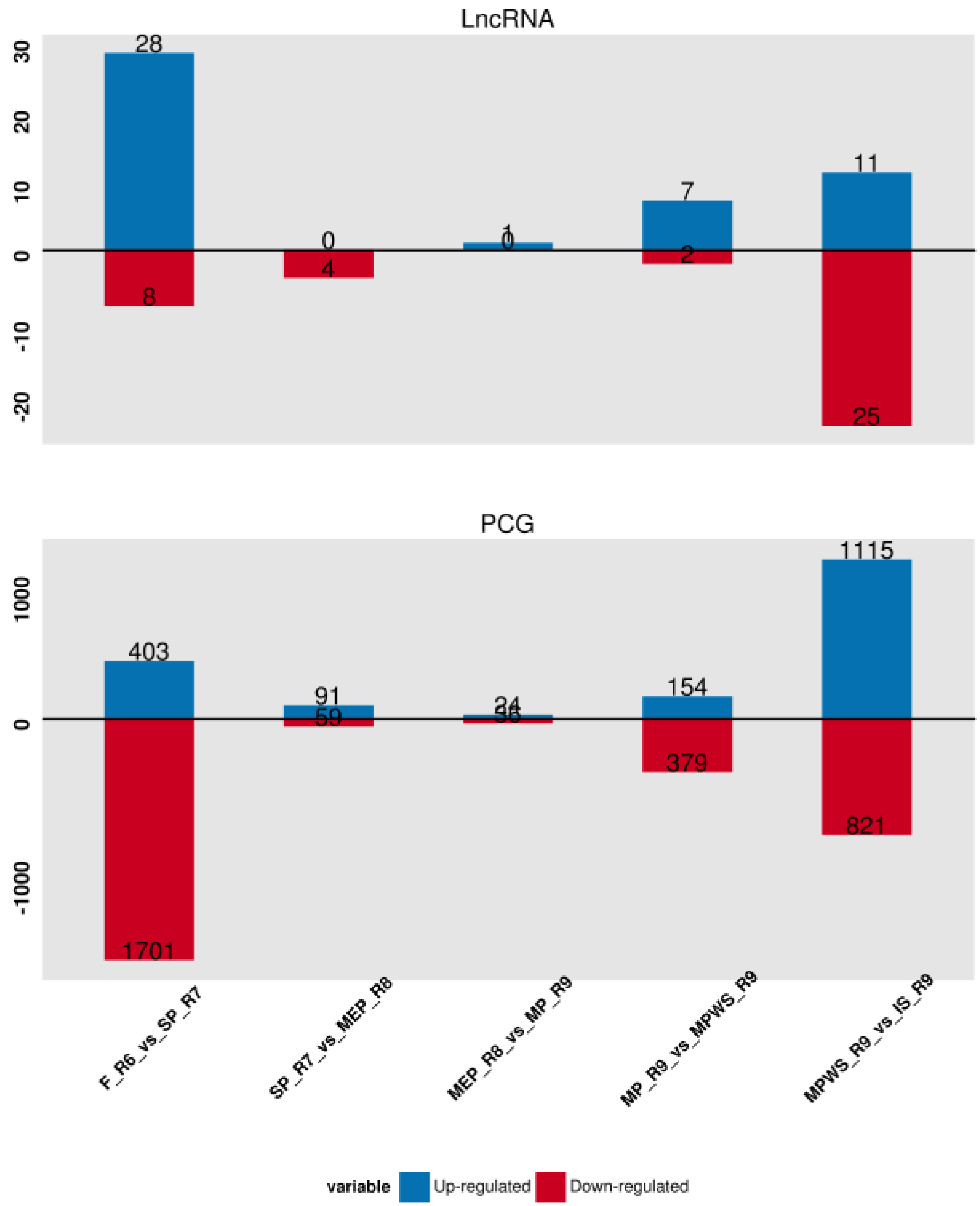

B. Pod/Seed samples transcriptome dynamics.

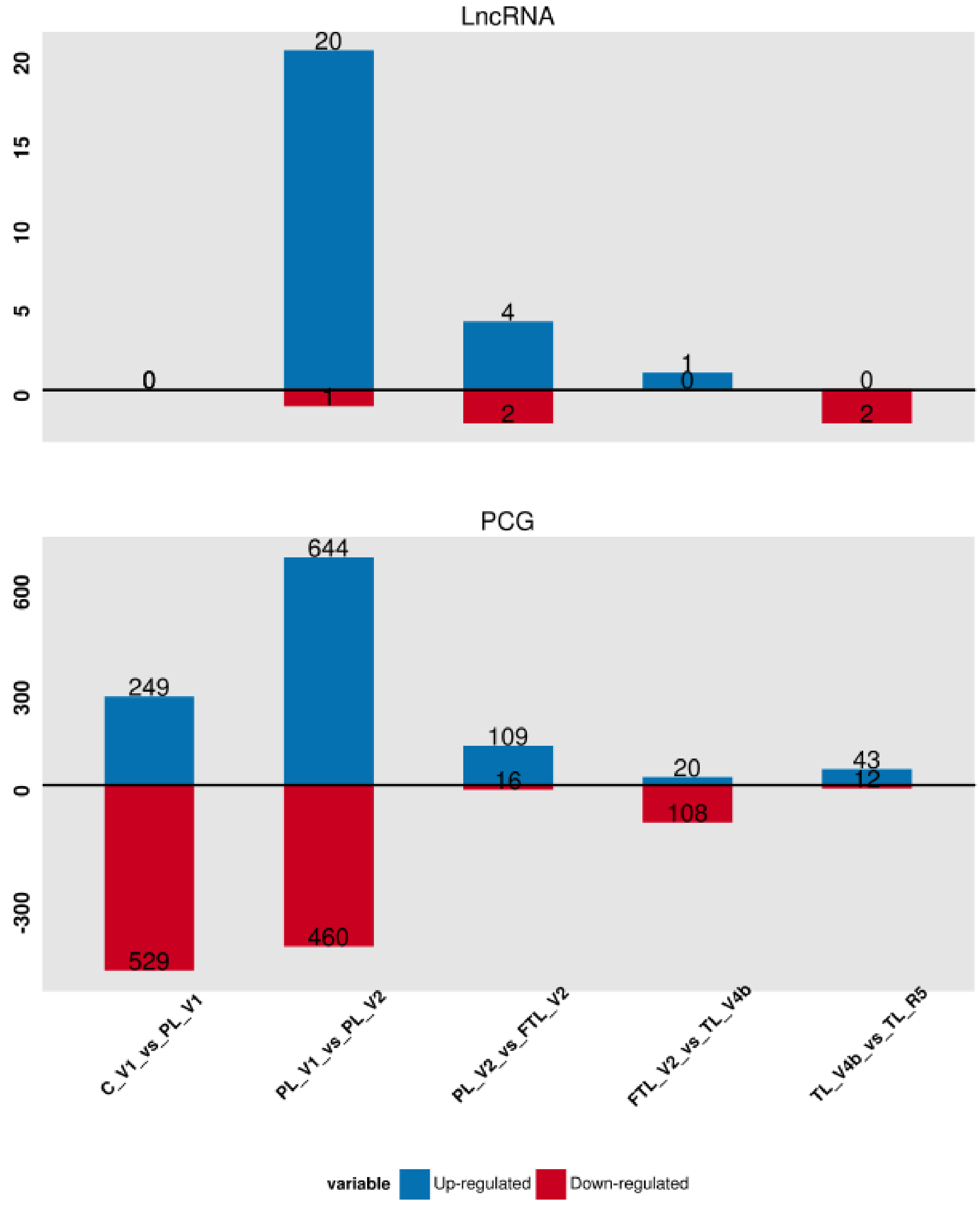

C. Leaf samples transcriptome dynamics.

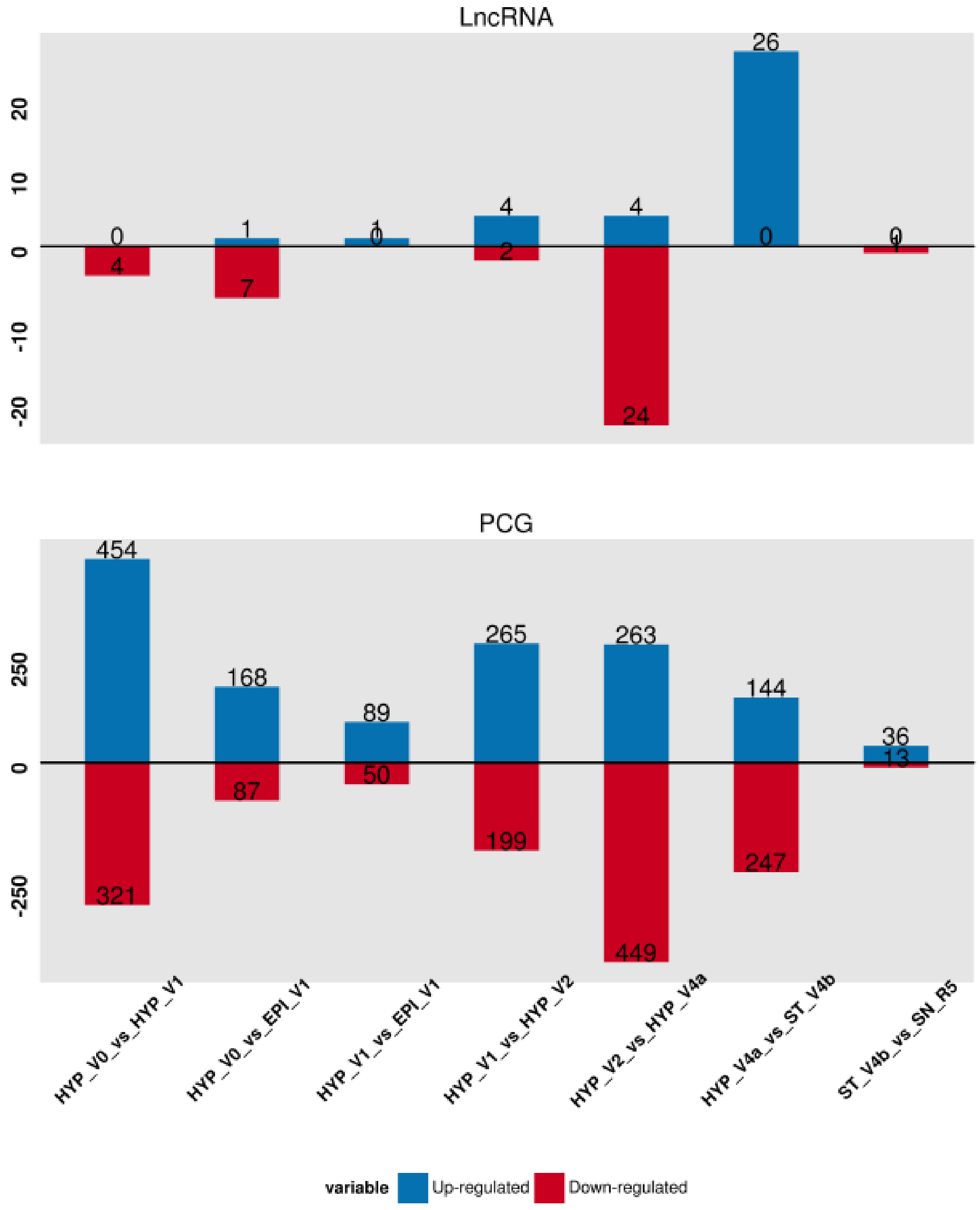

D. Stem samples transcriptome dynamics.

Figure S16. Transcriptome dynamics in the organ development. Number of up and down regulated genes comparing a given stage with the previous one in each organ. Number are shown separately for the protein-coding (PCG) and lncRNAs **A.** Root **B.** Pod/Seed **C.** Leaf **D.** Stem**.**

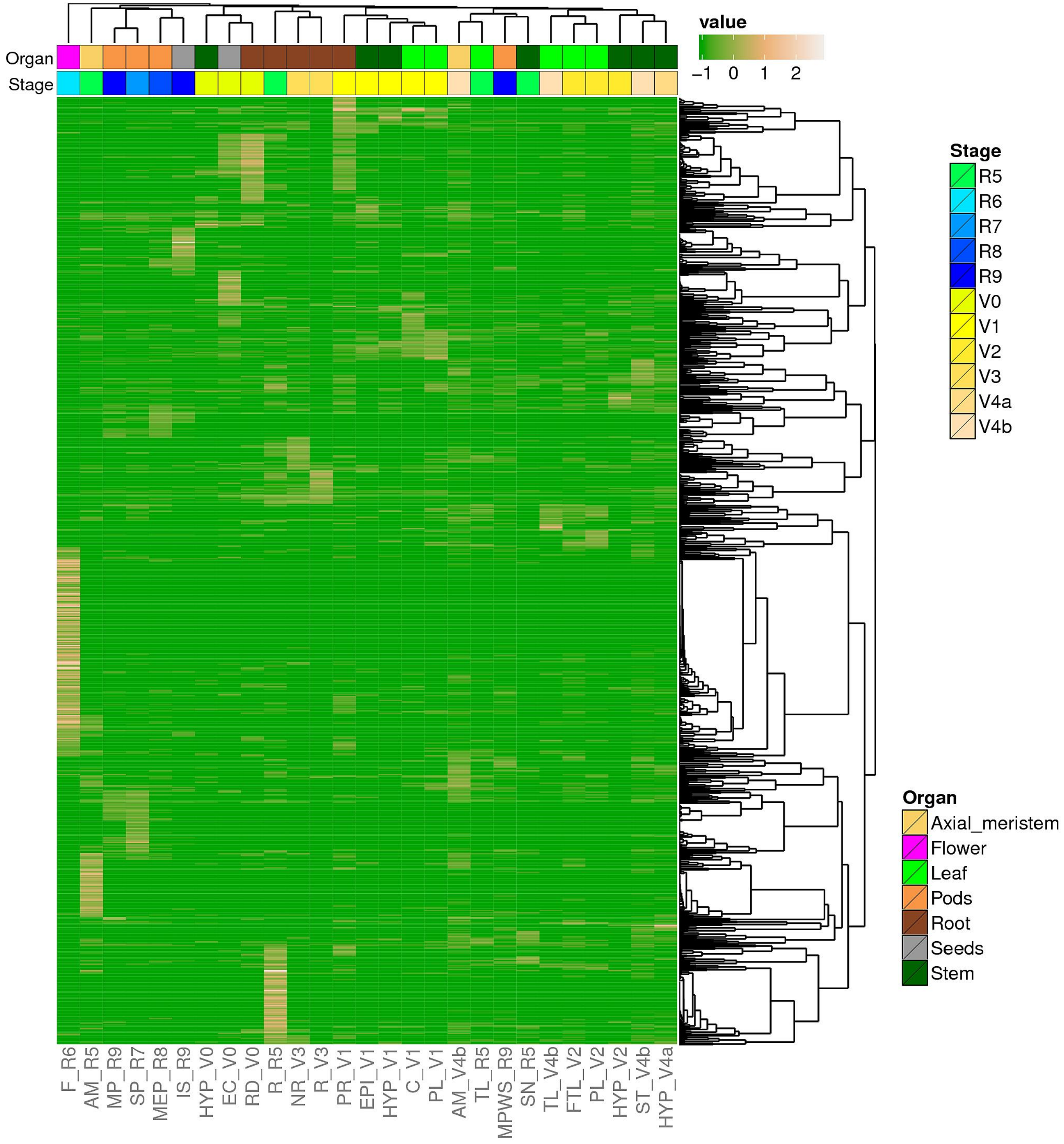

Figure S17. Stage-specific genes expression. Heat-map of log10(RPKM) values for genes specifically expressed only in one stage. Here we used 0.1 as a pseudo counts for the log transformation

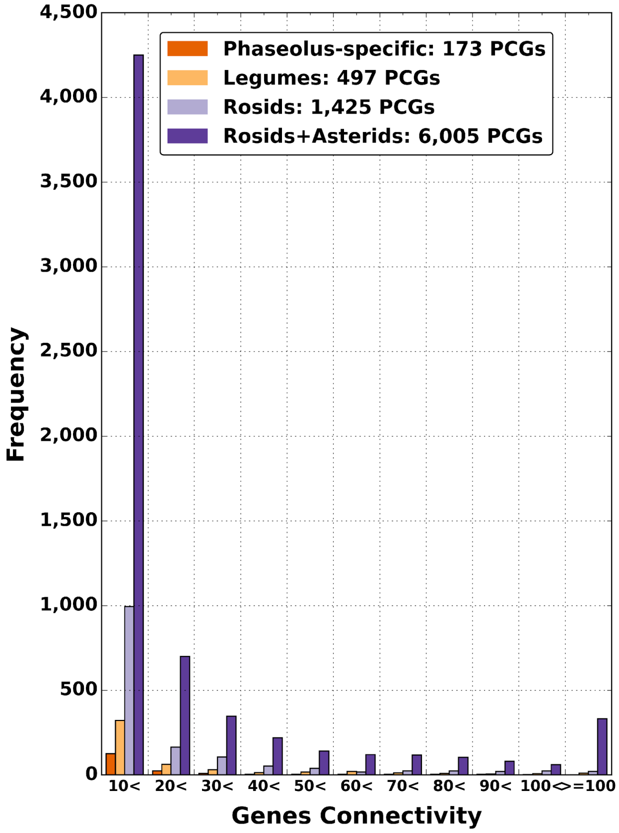

Figure S18. Gene connectivity for the co-expression network depending on the relative evolutionary age. The histogram shows the absolute number of genes assigned to one of the four evolutionary periods according to number of connections of those genes in the co-expression network.

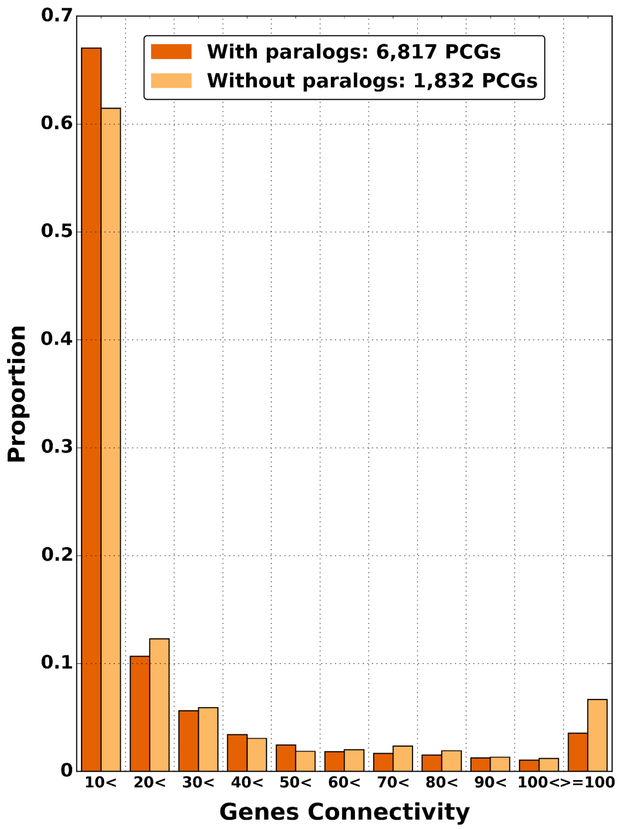

Figure S19. Genes with and without paralogous sequences in the co-expression network. The histogram shows the absolute number of genes divided into the ones with at least one paralog and the ones without any. Genes are grouped according to the number of connections in the co-expression network. Of note, only protein-coding genes were considered for this analysis.

Supplementary Tables

Table S1. Assembly input data. Paired data sets were filtered for redundancy. Column "Coverage" lists the sequence coverage for single reads and the library coverage for paired data.

Platform and read type	Insert size [kbp]	Number [million]	Coverage
Roche454 single reads	-	32	16.5
Roche454 pairs	8	2.36	29.6
	10	0.35	5.5
	20	0.31	9.7
SOLiD pairs	3	7.78	36.6
	5	3.99	31.3
Sanger pairs	1	0.17	0.3
Sanger BES	116	0.02	3.6

Supplementary Table S2. Summary table on the corrected homopolymers. Correction of homopolymers was performed using BAT93 genomic Illumina reads, 45x coverage.

**Number of corrected Ns**	6,517
**Number of corrected INDELs**	62,175
**polyA**	30,830(49.60%)
**polyC**	197 (0.32%)
**polyT**	30,914 (49.72%)
**polyG**	216 (0.35%)
**Max length**	17
**Min length**	3
**Mean length**	9
**Frequency**	1*10^5

Supplementary Table S3. Statistics genome assemblies before and after gap closing with GapCloser on the consensus-corrected assembly.

	#seq	N50 [bp]	L50	Average	Max [kb]	Sum [Mb]	%Ns
**Assembly 8.0, before gap closing**	134,674	6,360	18,155	3,181.3	251	428.4	18%
**Assembly 8.0, after gap closing**	114,804	10,159	12,622	3,966.09	251	455.3	13%

Supplementary Table S4. Summary on anchoring of scaffolds to linkage groups.

**Linkage group (LG)**	**Assembled length [Mb]**	**Assigned scaffolds**	**Anchored scaffolds (with markers)**	**% of assembly**
LG1	44.6	130	39	8.10%
LG2	48.0	190	60	8.70%
LG3	46.2	142	47	8.40%
LG4	39.0	193	35	7.10%
LG5	34.2	160	31	6.20%
LG6	32.8	98	32	5.90%
LG7	43.4	205	36	7.90%
LG8	50.6	221	49	9.20%
LG9	35.4	118	45	6.40%
LG10	34.7	181	25	6.30%
LG11	41.7	203	37	7.50%
**Total**	450.8	1,841	399	81%
**Unplaced scaffolds**	44.0	7,206	-	8%
**Unplaced contigs**	54.6	59,332	-	9.90%

Supplementary Table S5. Assembly metrics; all data was calculated with Ns.

	**n**	**L50**	**N50 [kb]**	**L90**	**N90**	**average**	**max [Mb]**	**Sum [Mb]**
**Assembly10**	68,379	324	433	8,892	2,023	8,038	3.2	549.6

Supplementary Table S6. Number and genomic coverage by mobile elements. TEs cover 33% of the genome, simple repeat cover ~2.5% of the genome.

	**#Number**	**%**	**bp(Mb)**	**%bp**
Mobile element	170,337	100	177.2	100
**Class I: Retroelement (RXX)**	129,652	76	146.4	82.6
DIRS (RYX)	201	0.1	0.09	0.05
LTR (RLX)	78,481	46	109	61.5
LINE (RIX)	16,564	9.7	13.8	7.8
SINE	374	0.2	0.12	0.07
Unclassified RXX	34,032	20	22.3	12.5
**Class II: DNA Transposones (DXX)**	15,758	9.2	14.1	12.6
TIR (DTX)	9,710	5.7	9.5	5.3
Helitrone (DHX)	3,145	1.9	3	1.7
Maverick (DMX)	153	0.1	0.1	0.05
Unclassified (DXX)	2,750	1.6	1.3	0.7
**Potential Host Gene**	17,547	10	11.3	6.3
**Unclassified**	7,380	4.3	5.4	3

Supplementary Table S7. Preparation of RNA-seq samples. Different total RNA samples of BAT93 growing at optimal conditions were collected at different times and stages of plant, pod (fruit), flower and seed development and then used for cDNA synthesis.

Sample*	Sowing date	Sowing hour	Collection date	Collection hour	Time to collection†	Plant developmental Stage‡	ng/ml
Left senescent trifoliate leaf	28/06/2010	9:30	31/08/2010	10:30	65 d	R9	342
Root neck	05/08/2010	9:30	13/08/2010	13:30	8 d	V3	143
Epicotyl	25/08/2010	12:30	31/08/2010	10:30	6 d	V2	128
Root neck	28/06/2010	9:30	13/08/2010	13:30	46 d	R8	173
Stem node	23/07/2010	9:30	27/08/2010	9:30	33 d	R5	219
Intermediate senescent trifoliate leaf	28/06/2010	9:30	25/08/2010	10:00	58 d	R9	263
Right senescent trifoliate leaf	28/06/2010	9:30	25/08/2010	10:00	58 d	R9	379
Stem	23/07/2010	9:30	27/08/2010	9:30	33 d	R5	244
Axial meristem	28/06/2010	9:30	05/08/2010	14:00	38 d	R6	402
Pod stage 0	28/06/2010	9:30	05/08/2010	14:00	38 d	R7	454
Pod stage 1	28/06/2010	9:30	05/08/2010	14:00	38 d	R7	504
Stem node	28/06/2010	9:30	05/08/2010	14:00	38 d	R7	374
Stem node	28/06/2010	9:30	13/08/2010	13:30	46 d	R8	533
Right trifoliate leaf	28/06/2010	9:30	13/08/2010	13:30	46 d	R8	-
Hypocotyl	14/06/2010	9:30	18/06/2010	14:00	4 d	V2	441
Epicotyl	05/08/2010	9:30	13/08/2010	13:30	8 d	V3	418
Pod stage 6	28/06/2010	9:30	25/08/2010	10:00	58 d	R9	888
Root	05/08/2010	9:30	13/08/2010	13:30	8 d	V3	490
Left trifoliate leaf	28/06/2010	9:30	13/08/2010	13:30	46 d	R8	814
Intermediate trifoliate leaf	23/07/2010	9:30	27/08/2010	9:30	33 d	R5	692
Embryo	25/08/2010	20:00	26/08/2010	8:00	12 h	V0	711
Pod stage 7	28/06/2010	9:30	25/08/2010	10:00	58 d	R9	568
Root	28/06/2010	9:30	10/08/2010	10:30	12 d	V4	823
Immature seed	28/06/2010	9:30	13/08/2010	13:30	46 d	R8	716
Root	28/06/2010	9:30	05/08/2010	14:00	38 d	R6	821
Epicotyl	27/08/2010	10:00	31/08/2010	10:00	4 d	V1	603
Open flower	28/06/2010	9:30	05/08/2010	14:00	38 d	R6	-
Left trifoliate leaf	28/06/2010	9:30	05/08/2010	14:00	38 d	R6	692
Pod stage 5	28/06/2010	9:30	13/08/2010	13:30	46 d	R8	890
Intermediate trifoliate leaf	28/06/2010	9:30	05/08/2010	14:00	38 d	R6	772
Axial meristem	23/07/2010	9:30	27/08/2010	9:30	33 d	R5	905
Hypocotyl	27/08/2010	10:00	31/08/2010	10:00	4 d	V1	932

Radicle	25/08/2010	10:00	27/08/2010	10:00	48 h	V1	1376
Primary leaf	14/06/2010	9:30	18/06/2010	14:00	4 d	V2	1219
Radicle	14/06/2010	9:30	18/06/2010	14:00	4 d	V2	1653
Pod stage 2	28/06/2010	9:30	05/08/2010	14:00	38 d	R7	1445
First trifoliate leaf	28/06/2010	9:30	06/07/2010	12:00	8 d	V3	2836
Pod stage 4	28/06/2010	9:30	05/08/2010	14:00	38 d	R7	2988
Primary leaf	05/08/2010	9:30	13/08/2010	13:30	8 d	V3	3258
Pod stage 3	28/06/2010	9:30	05/08/2010	14:00	38 d	R7	3635
First trifoliate leaf	28/06/2010	9:30	10/08/2010	10:30	12 d	V4	4570

[*] Stages of pod formation (R7): 0-1 = first pods with the flower’s corolla hanging or detached, 2 = pods from the upper part of the plant, 3-4 = pods with different in length and with little growth of the seeds; stages of pod filling (R8): 5 = pods with immature seeds start to acquire the seed features which characterize the cultivar; stages of maturity (R9): 6-7 = characterized by discoloration and drying of the pods.

[†] d: days; h: hours

[‡] Stages of the vegetative phase: V0= germination, V1=emergence, V2= primary leaves, V3= first trifoliate leaf, and V4= third trifoliate leaf; stages of the reproductive phase: R5= preflowering, R6= flowering, R7= pod formation, R8= pod filling, and R9= maturity.

Supplementary Table S8. Description of RNAseq samples. RNA-Seq samples used for annotation, transcriptomic and lncRNA analysis, 61 different samples in total from 34 different conditions (organs and development stages). The 27 samples with replicates (in blue color) were used for the expression analysis.

Sample name	Stage	Organ	Replicates	Collection time	Organ
EC_V0	V0	Embryo + Cotyledons	2	48 hours	Seed
HYP_V0	V0	Hypocotyl	2	48 hours	Stem
RD_V0	V0	Radicle	2	48 hours	Root
PL_V1	V1	Primary leaf	2	6 days	Leaf
EPI_V1	V1	Epicotyl	2	6 days	Stem
PR_V1	V1	Primary root	2	6 days	Root
C_V1	V1	Cotyledons	2	6 days	Leaf
HYP_V1	V1	Hypocotyl	2	6 days	Stem
HYP_V2	V2	Hypocotyl	2	10 days	Stem
FTL_V2	V2	First trifoliate leaf	2	10 days	Leaf
PL_V2	V2	Primary leaf	2	10 days	Leaf
R_V2	V2	Root	1	10 days	Root
NR_V3	V3	Neck of the root	2	14 days	Root
TL_V3	V3	Trifoliate leaf	1	14 days	Leaf
R_V3	V3	Root	2	14 days	Root
ST_V3	V3	Stem	1	14 days	Stem
TL_V4a	V4a	Trifoliate leaf	1	29 days	Leaf
HYP_V4a	V4a	Hypocotyl	2	29 days	Stem
R_V4a	V4a	Root	1	29 days	Root
TL_V4b	V4b	Trifoliate leaf	2	35 days	Leaf
SN_V4b	V4b	Stem node	1	35 days	Stem
ST_V4b	V4b	Stem	2	35 days	Stem
AM_V4b	V4b	Axial meristem	2	35 days	Axial mer.
TL_R5	R5	Trifoliate leaf	2	43 days	Leaf
SN_R5	R5	Stem node	2	43 days	Stem
R_R5	R5	Root	2	43 days	Root
AM_R5	R5	Axial meristem	2	43 days	Axial mer.
FB_R5	R5	Flower bud	1	50 days	Bud
F_R6	R6	Flower	2	53 days	Flower
SP_R7	R7	Small pod	2	60 days	Pod
MEP_R8	R8	Medium pod	2	64 days	Pod
IS_R9	R9	Immature seed	2	79 days	Seed
MPWS_R9	R9	Mature pod without seeds	2	79 days	Pod
MP_R9	R9	Mature pod	2	86 days	Pod

Supplementary Table S9. 454 samples description. Selected organs at different developmental stages for RNA collection.

		**Number of samples**	**Example**
Library 1	Developmental stages	41	Embryo, radicle, mature root, epi/hypocotyl, immature/mature pods, seeds, cotyledons, trifoliate leaves, senescent leaves.
Library 2	Biotic stress	41	Inoculation with different strains of *Pseudomonas syringae* pv. *phaseolicola*; *Xanthomonas campestris* pv. *phaseolicola*, *Rhizoctonia solani*, *Fusarium solani*
	Abiotic stress	80	P deficit, temperature stress, water deprivation, NifA/H mutants; presence/absence of Rhizobia and Mycorryza.

Supplementary Table S10. 454 Different total RNA samples of BAT93 from biotic stress treatments at different times were used for cDNA synthesis using QIAquick (Qiagen), and cDNA was then normalized using DSN normalization method[79]. Normalization included cDNA denaturation/reassociation, treatment by duplex-specific nuclease [80] and amplification of normalized by fraction PCR.

**Biotic stress**	**Treatment-Inoculation**	**Time after treatment***	**Organ**	**Time after sowing**
**B1-1**	Pool of 9 pathogenic races of *Pseudomonas syringae* pv. *phaseolicola*	48 h	Primary leaf	2 w
**B1-2**	Control for *P. syringae* pv. *phaseolicola*	48 h	Primary leaf	2 w
**B2-1**	Pathogenic isolate of *Xanthomonas campestris* pv. *phaseolicola*	0 h, 24 h, 48 h, 72 h, 6 d, 8 d, 10 d, 13 d, 15 d	Trifoliate leaf	7 w
**B2-2**	Control for *X. campestris* pv. *phaseolicola*	0 h, 24 h, 48 h, 72 h, 6 d, 8 d, 10 d, 13 d, 15 d	Trifoliate leaf	7 w
**B2-3**	Pathogenic isolate of *X. campestris pv. phaseolicola*	1 h, 4 d, 5 d, 6 d, 8 d, 9 d, 11 d, 12 d	Medium Pod	9 w
**B2-4**	Control for *X. campestris* pv. *phaseolicola*	1 h, 12 d	Medium Pod	9 w
**B2-5**	Pool of pathogenic isolates of *X. campestris pv. phaseolicola*	pool different times	Trifoliate leaf	7 w
**B2-6**	Control for *X. campestris pv. phaseolicola*	pool different times	Trifoliate leaf	7 w
**B3-1**	Pathogenic race 3 *P. syringae pv. phaseolicola*	0 h, 24 h, 48 h, 72 h, 96 h, 7 d, 9 d, 15 d	Primary leaf	2 w
**B3-2**	Pathogenic race 6 *P. syringae pv. phaseolicola*	0 h, 24 h, 48 h, 72 h, 96 h, 7 d, 9 d, 15 d	Primary leaf	2 w
**B3-3**	Control for *P. syringae pv. phaseolicola*	0 h, 24 h, 48 h, 72 h, 96 h, 7 d, 9 d, 15 d	Primary leaf	2 w
**B4-1**	Pathogenic isolate of *Uromyces appendiculatus*	24 h	Trifoliate leaf	3 w
**B4-2**	Control for *U. appendiculatus*	24 h	Trifoliate leaf	3 w
**B5-1**	Pathogenic isolate of *Fusarium solani f.* sp *pisi*	48 h	Root	2 w
**B5-2**	Control of *F. solani f.* sp *pisi*	48 h	Root	2 w
**B6-1**	Pathogenic isolate of *Rhizoctonia solani*	pool 8 h, 16 h and 24 h	Root	2 w
**B6-2**	Control of *R. solani*	pool 8 h, 16 h and 24 h	Root	2 w
**B6-3**	Pathogenic isolate of *R. solani*	pool 8 h, 16 h and 24 h	Cotyledons, root and stem	2 w
**B6-4**	Control of *R. solani*	pool 8 h, 16 h and 24 h	Cotyledons, root and stem	2 w
**B7-1**	No pathogenic isolate of binucleated *R. solani*	pool 8 h, 16 h and 24 h	Primary leaf	5 d
**B7-2**	Control of binucleated *R. solani*	pool 8 h, 16 h and 24 h	Primary leaf	5 d
**B7-3**	No pathogenic isolate of binucleated *R. solani*	pool 8 h, 16 h and 24 h	Root	5 d
**B7-4**	Control of binucleated *R. solani*	pool 8 h, 16 h and 24 h	Root	5 d
**B8-1**	Pathogenic isolate o*f Fusarium oxysporum f.* sp *phaseoli*	48 h	Primary leaf	2 w
**B8-2**	Control of *F. oxysporum f.* sp *phaseoli*	48 h	Primary leaf	2 w
**B9-1**	No pathogenic race 256 of *C. lindemuthianum*	pool intervals between 15 m (96 h)	Hypocotyl	2 w
**B9-2**	Pathogenic race 1088 of *C. lindemuthianum*	pool intervals between 15 m (96 h)	Hypocotyl	2 w
**B9-3**	Control of *C. lindemuthianum*	pool intervals between 15 m (96 h)	Hypocotyl	2 w
**B10-1**	Pathogenic race 9 *C. lindemuthianum*	48 h	Primary leaf	2 w
**B10-2**	Pathogenic race 6 *C. lindemuthianum*	48 h	Primary leaf	2 w
**B10-3**	Pathogenic race 453 *C. lindemuthianum*	48 h	Primary leaf	2 w
**B10-4**	Pathogenic race 19 *C. lindemuthianum*	48 h	Primary leaf	2 w
**B10-5**	Pathogenic race 102 *C. lindemuthianum*	48 h	Primary leaf	2 w
**B10-6**	Pathogenic race 73 *C. lindemuthianum*	48 h	Primary leaf	2 w
**B10-7**	Pathogenic race 7 *C. lindemuthianum*	48 h	Primary leaf	2 w
**B10-8**	Pathogenic race 38 *C. lindemuthianum*	48 h	Primary leaf	2 w

**B10-9**	Pathogenic race 3 *C. lindemuthianum*	48 h	Primary leaf	2 w
**B10-10**	Pathogenic race 89 *C. lindemuthianum*	48 h	Primary leaf	2 w
**B10-11**	Control of *C. lindemuthianum*	48 h	Primary leaf	2 w
**B11-1**	Pathogenic isolate of *Erysiphe diffusa*	48 h	Primary leaf	2 w
**B11-2**	Control of *E. diffusa*	48 h	Primary leaf	2 w

*m, minutes; h, hours; d, days; w, weeks.

Supplementary Table S11. 454 Sequencing. Different total RNA samples of BAT93 from abiotic stress and symbiotic association treatments at different times were used for cDNA synthesis using QIAquick (Qiagen), and cDNA was then normalized using DSN normalization method [79]. Normalization included cDNA denaturation/reassociation, treatment by duplex-specific nuclease [80] and amplification of normalized by fraction PCR.

Abiotic stress	Treatment-Inoculation	Time after treatment*	Organ	Time after sowing/inoculation*
**A1-1**	Phosphorus deficit	1 d	Root	2 w
**A1-2**	Phosphorus deficit	4 d	Root	2 w
**A1-3**	Phosphorus deficit and non-deficit recovery	pool 4 d and 8 d	Root	2 w
**A1-4**	Control of phosphorus deficit	pool 4 d and 8 d	Root	2 w
**A1-5**	Phosphorus deficit	1 d	Nodule	2 w
**A1-6**	Phosphorus deficit	4 d	Nodule	2 w
**A1-7**	Phosphorus deficit and non-deficit recovery	pool 4 d and 8 d	Nodule	2 w
**A1-8**	Control of phosphorus deficit	pool 4 d and 8 d	Nodule	2 w
**A2-1**	Phosphorus deficit: PvPHR1 dependent and independent transcriptome	1 d	Root	2 w
**A2-2**	Phosphorus deficit: PvPHR1 dependent and independent transcriptome	4 d	Root	2 w
**A2-3**	Control of phosphorus deficit	1 d	Root	2 w
**A2-4**	Control of phosphorus deficit	4 d	Root	2 w
**A3-1**	45ºC	15 m	Primary leaf and stem	2 w
**A3-2**	45ºC	30 m	Primary leaf and stem	2 w
**A3-3**	45ºC	60 m	Primary leaf and stem	2 w
**A3-4**	45ºC	15 m	Root	2 w
**A3-5**	45ºC	30 m	Root	2 w
**A3-6**	45ºC	60 m	Root	2 w
**A3-7**	45ºC	15 m	Nodule	2 w
**A3-8**	45ºC	30 m	Nodule	2 w
**A3-9**	45ºC	60 m	Nodule	2 w
**A3-10**	100 mM NaCl	24 h	Primary leaf and stem	2 w
**A3-11**	100 mM NaCl	4 d	Primary leaf and stem	2 w
**A3-12**	150 mM NaCl	24 h	Primary leaf and stem	2 w
**A3-13**	150 mM NaCl	4 d	Primary leaf and stem	2 w
**A3-14**	200 mM NaCl	24 h	Primary leaf and stem	2 w
**A3-15**	200 mM NaCl	4 d	Primary leaf and stem	2 w
**A3-16**	Control NaCl	24 h	Primary leaf and stem	2 w
**A3-17**	Control NaCl	4 d	Primary leaf and stem	2 w
**A3-19**	Tbgs 2 µM	5 h	Primary leaf and stem	2 w
**A3-20**	45ºC	15 m	Primary leaf and stem	1 w
**A3-21**	45ºC	30 m	Primary leaf and stem	1 w
**A3-22**	45ºC	60 m	Primary leaf and stem	1 w
**A4-1**	Drought (50% water lost at post-harvest)	72 h	Root	58 d
**A4-2**	Control of drought	72 h	Root	58 d
**A4-4**	Water deficit (1/12 water)	72 h	Root	5 d
**A4-5**	Control of water deficit	72 h	Root	5 d
**A4-6**	Drought (50% water lost at post-harvest)	72 h	Stem	58 d
**A4-7**	Control of drought	72 h	Stem	58 d
**A4-9**	Darkness and water deficit (1/12 water)	72 h	Stem	1 w
**A4-10**	Control of darkness and water deficit	72 h	Stem	1 w
**A4-11**	Drought (50% water lost at post-harvest)	72 h	Leaf	58 d
**A4-12**	Control of drought	72 h	Leaf	58 d
**A5-1**	Wild isolate *Rhizobium etli*	2 h, 12 h, 24 h	Root	2 d
**A5-2**	Wild isolate *R. etli*	48 h, 72 h, 5 d	Root	2 d
**A5-3**	Wild isolate *R. etli*	2 h, 12 h, 24 h	Root	2 d

**A5-4**	Control of *R. etli*	48 h, 72 h, 5 d	Root	2 d
**A5-5**	Mutant isolate *R. etli* CE168	2 h, 12 h, 24 h	Root	2 d
**A5-6**	Mutant isolate *R. etli* CE168	48 h, 72 h, 5 d	Root	2 d
**A5-7**	Mutant isolate *R. etli* CE395	2 h, 12 h, 24 h	Root	2 d
**A5-8**	Mutant isolate *R. etli* CE395	48 h, 72 h, 5 d	Root	2 d
**A5-9**	NOD factors (*R. etli*)	30 m, 1 h, 2 h, 4 h, 6 h, 9 h	Root	2 d
**A5-10**	NOD factors (*R. etli*)	24 h, 72 h	Root	2 d
**A5-11**	Control of NOD factors	30 m, 1 h, 2 h, 4 h, 6 h, 9 h	Root	2 d
**A5-12**	Control of NOD factors	24 h, 72 h	Root	2 d
**A6-1**	Isolate *R. etli*	24 h	Root	2 d
**A6-2**	Control *R. etli*	24 h	Root	2 d
**A6-3**	Control silenced *R. etli*	24 h	Root	2 d
**A6-4**	Silenced *R. etli*	24 h	Root	2 d
**A7-1**	Wild isolate *R. etli* CE3	12 d	Nodule	2 d
**A7-2**	Wild isolate *R. etli* CE3	25 d	Nodule	2 d
**A7-3**	Mutant isolate *R. etli* NifA	12 d	Nodule	2 d
**A7-4**	Mutant isolate *R. etli* NifA	25 d	Nodule	2 d
**A7-5**	Control of *R. etli* NifA	12 d	Nodule	2 d
**A7-6**	Control of *R. etli* NifA	25 d	Nodule	2 d
**A7-7**	Auxotrophic *R. etli* lys- mutant	12 d	Nodule	2 d
**A7-8**	Auxotrophic *R. etli* lys- mutant	25 d	Nodule	2 d
**A7-9**	Auxotrophic *R. etli* leu- mutant	12 d	Nodule	2 d
**A7-10**	Auxotrophic *R. etli* leu- mutant	25 d	Nodule	2 d
**A7-11**	*R. etli* OtsA^{-} mutant	12 d	Nodule	2 d
**A7-12**	*R. etli* OtsA^{-} mutant	25 d	Nodule	2 d
**A7-15**	*R. etli* OtsA+ mutant	12 d	Nodule	2 d
**A7-16**	*R. etli* OtsA+ mutant	25 d	Nodule	2 d
**A7-17**	*R. etli* OtsA^{-} mutant	15 d	Primary leaf	2 w
**A7-18**	*R. etli* OtsA+ mutant	15 d	Primary leaf	2 w
**A8-1**	Symbiotic arbuscular mycorrhiza	4 w	Root	2 d
**A8-2**	Symbiotic arbuscular mycorrhiza	4 w	Primary leaf	2 d
**A8-3**	Control of symbiotic arbuscular mycorrhiza	4 w	Root	2 d
**A8-4**	Control of symbiotic arbuscular mycorrhiza	4 w	Primary leaf	2 d

*m, minutes; h, hours; d, days; w, weeks.

Supplementary Table S12. 454 transcriptome assembly results.

**Reads**			
numberAssembled	1,830,138	numberSingleton	76,398
numberPartial	100,758	numberOutlier	17,436
numberRepeat	14,727	numberTooShort	64,221
**Isotigs**		**Isogroups**	
numberOfIsotigs	28,601	numberOfIsogroups	21,268
avgContigCnt	1.6	avgIsotigCnt	1.3
largestContigCnt	9	largestIsotigCnt	32
numberWithOneContig	17,959	numberWithOneIsotig	16,177
avgIsotigSize	1,047		
largestIsotigSize	10,971		

Supplementary Table S13. Accuracy of gene predictions. Accuracy was calculated on a bean artificial contig consisting of 138 concatenated bean test sequences with approximately 800 nucleotides of sequence between each of the gene models using the *ab initio* programs GeneID, AUGUSTUS and GlimmerHMM given two different plant-derived parameter files and a human matrix. The accuracy of SGP2 (homology evidence-based prediction tool that used the *A. thaliana* genome as reference) and that of AUGUSTUS (using RNASeq and transcript evidence i.e. "+hints") was also tested for accuracy on the same set of sequences (SN & SP: sensitivity & specificity at nucleotide level; SNe & SPe: sensitivity & specificity at exon level; SNg & SPg: sensitivity & specificity at gene level).

**Program/param**	**SN**	**SP**	**SNe**	**SPe**	**SNg**	**SPg**
GeneID bean	0.93	0.95	0.81	0.84	0.28	0.28
SGP2 bean / *Arabidopsis*	0.97	0.97	0.87	0.88	0.37	0.39
AUGUSTUS bean	0.94	0.97	0.84	0.88	0.32	0.37
AUGUSTUS+hints bean	0.98	0.96	0.89	0.89	0.41	0.43
Glimmer bean	0.87	0.97	0.67	0.85	0.25	0.23
GeneID *Arabidopsis*	0.85	0.95	0.67	0.78	0.13	0.18
GeneID human	0.53	0.94	0.24	0.67	0.04	0.09

Supplementary Table S14. Weights used by EVM to created consensus coding sequence (CDS) models.

**Type**	**Source**	**Weight**
**ABINITIO_PREDICTION**	AUGUSTUS	2
**ABINITIO_PREDICTION**	AUGUSTUSHints	4
**ABINITIO_PREDICTION**	GlimmerHMM	1
**ABINITIO_PREDICTION**	GeneID_v1.4	2
**ABINITIO_PREDICTION**	SGP2	3
**PROTEIN**	GeneWise	6
**TRANSCRIPT**	PASA	10

Supplementary Table S15. Assembly and annotation statistics. Statistics on protein gene annotation v10F and *P. vulgaris* genome assembly v.10.

	***P. vulgaris* (assembly 10 hardmasked)**	**scaffolds**	**contigs**
Genome length (Mbases)	549.604	494.957	54.647
# of scaffolds/contigs	68,379	9,047	59,332
% repeats nts. (Ns) in the genome	34.96	36.99	16.33
**# of genes**	**30,491**	29,569	922
gene density (genes/Kbase)	0.055	0.060	0.017
**# of transcripts**	**66,634**	65,685	949
Transcripts/gene (range)	2.19 (SD 2.47) (1-46) - 32.72% of the genes have > 1 transcript-)	2.22 (SD 2.50) (1-46) - 33.66% of the genes have > 1 transcript-)	1.03 (SD 0.20)(1-3) - 1.47% of the genes have > 1 transcript-)
Number of transcripts with UTRs	55,952 (83.97%)	55,613 (84.67%)	338 (35.62%)
**Number of proteins**	**53,904**	52,960	944
Avg. length of proteins (range)	443.55 aa. (SD 332.42) (36 – 5,456)	447.68 aa. (SD 333.88) (36 – 5,456)	158.02 aa. (SD 80.08) (50 – 518)
Avg. length of complete proteins (range)	451.14 aa. (SD 333.53) (36 – 5,456)	452.65 aa. (SD 333.72) (36 – 5,456)	168.15 aa. (SD 84.73) (50 – 518)
Number of partial proteins (not starting with "M")	988 (1.48%)	721 (1.10%)	267 (28.13%)
Number of partial proteins (no terminal STOP codon)	1,180 (1.77%)	1,027 (1.13%)	437 (46.40%)
Number of partial proteins (not starting with an M -and- no terminal STOP codon)	192 (0.29%)	92 (0.14%)	100 (10.54%)
Number of partial proteins (not starting with an M -or- no terminal STOP codon)	1,976 (2.97%)	1,372 (2.09%)	917 (63.65%)
Number of coding exons	401,852	400,379	1,473
Number of introns	343,822	334,694	524
Number of UTRs (spliced)	152,152	151,601	551
Number of single-exon genes	8,604 (28.22%)	8,024 (27.14%)	580 (62.91%)
Exons/transcript (range) (excludes single-exon genes)	6.78 (SD 5.29) (2 – 77)	6.80 (SD 5.29) (2 – 77)	2.42 (SD 0.74) (2 – 6)
Introns/transcript (range)	5.78 (SD 5.29) (1 – 76)	5.80 (SD 5.29) (1 – 76)	1.42 (SD 0.74) (1 – 5)
“spliced” UTRs/transcript (range)	2.72 (SD 0.94) (1 - 10)	2.73 (SD 0.94) (1 - 10)	1.63 (SD 0.55) (1 - 3)
Avg. length of introns (range)	519.59 (SD 1,206.36) (21 – 95,451)	520.13 (SD 1,207.22) (21 – 95,451)	172.95 (SD 147.91) (42 – 1,033)
Avg. length of exons (excluding single-exon genes) (range)	205.27 (SD 269.82) (1 – 7,991)	205.30 (SD 270.03) (1 – 7,991)	192.46 (SD 149.86) (3 – 1,554)
Avg. length of CDS (range)	1,330.66 (SD 997.25) (108 – 16,368)	1,343.04 (SD 998.64) (108 – 16,368)	474.30 (SD 240.21) (150 – 1,554)
Avg. length of UTRs (range)	312.31 (SD 419.18) (1 – 12,653)	312.79 (SD 419.71) (1 – 12,653)	179.96 (SD 186.77) (1 – 1,251)
Avg. length of primary transcripts (range)	5,193.23 (SD 5,183.28) (150 – 98,896)	5,258.41 (SD 5,191.76) (150 – 98,896)	681.95 (SD 360.46) (152 – 1,989)
G+C content exonic	44.45% (SD 4.92%)	44.45% (SD 4.87%)	44.53% (SD 7.67%)
G+C content intronic	31.26% (SD 4.86%)	31.26% (SD 4.85%)	29.60% (SD 9.07%)
G+C content genomic	36.30% (SD 8.93%)	36.31% (SD 8.93%)	33.58% (SD 8.16%)
G+C content UTRs	34,93% (SD 3.71%)	34,93% (SD 3.59%)	34.83% (SD 12.56%)

Supplementary Table S16. Functional annotation statistics. KO – KEGG orthology groups.

Number of transcripts/genes	66,634 / 30,491
**Annotated transcripts/genes**	**62,713 (94.12%) / 26,635 (87.35%)**
Transcripts/genes with Interpro signatures	57,206 / 23,157
Transcripts/genes with Blast2GO or KEGG definition	47,360 / 18,733
Transcripts/genes with Blast2GO definition	47,254 / 18,683
Transcripts/genes with KEGG definition	2,788 / 1,368
Transcripts/genes assigned to KO groups	16,233 / 7,847
Transcripts/genes with GO terms association	50,635 / 20,308
Conserved domains signatures	22,704 / 9,293
Conserved features signatures	24,583 / 9,630
Signal peptide site	3,075 / 2,367
Proteins/genes with reactome data	711 / 711

Supplementary Table S17. GO term annotation. **A**. Number of GO terms associated to each ontology **B**. Top 20 GO terms more frequently associated to proteins.

A

**Term type**	**#Proteins**
Biological process	40,279
Cellular component	31,465
Molecular function	43,684

B

**GO term id**	**GO term description**	**# Proteins**
	**Biological process**	
GO:0008152	metabolic process	5293
GO:0055114	oxidation-reduction process	4035
GO:0006468	protein phosphorylation	3450
GO:0006355	regulation of transcription, DNA-templated	3365
GO:0055085	transmembrane transport	1862
GO:0006200	ATP catabolic process	1503
GO:0006810	transport	1335
GO:0006508	proteolysis	1205
GO:0005975	carbohydrate metabolic process	1180
GO:0009651	response to salt stress	1126
GO:0046686	response to cadmium ion	1056
GO:0016310	phosphorylation	1020
GO:0009737	response to abscisic acid	888
GO:0007165	signal transduction	797
GO:0009409	response to cold	727
GO:0006351	transcription, DNA-templated	694
GO:0009414	response to water deprivation	679
GO:0034220	ion transmembrane transport	678
GO:0032259	methylation	670
GO:0006412	translation	647
	**Molecular function**	
GO:0005515	protein binding	9148
GO:0005524	ATP binding	7484
GO:0003677	DNA binding	4398
GO:0008270	zinc ion binding	3693
GO:0016772	transferase activity, transferring phosphorus-containing groups	3475
GO:0004672	protein kinase activity	3238
GO:0003824	catalytic activity	3161
GO:0004674	protein serine/threonine kinase activity	2729
GO:0003676	nucleic acid binding	2480
GO:0046872	metal ion binding	2486
GO:0003676		2480

GO:0005488	binding	2247
GO:0016491	oxidoreductase activity	1864
GO:0000166	nucleotide binding	1660
GO:0003700	sequence-specific DNA binding transcription factor activity	1658
GO:0016787	hydrolase activity	1366
GO:0003723	RNA binding	1296
GO:0043565	sequence-specific DNA binding	1136
GO:0046983	protein dimerization activity	1041
GO:0003682	chromatin binding	1040
GO:0005506	iron ion binding	776
	**Cellular component**	
GO:0005634	nucleus	7004
GO:0016021	integral component of membrane	5199
GO:0005886	plasma membrane	4871
GO:0016020	membrane	4132
GO:0005829	cytosol	3656
GO:0009507	chloroplast	2603
GO:0009506	plasmodesma	2580
GO:0005737	cytoplasm	2317
GO:0005739	mitochondrion	2051
GO:0005794	Golgi apparatus	1444
GO:0005622	intracellular	1425
GO:0009570	chloroplast stroma	1208
GO:0009941	chloroplast envelope	1193
GO:0005774	vacuolar membrane	1087
GO:0005783	endoplasmic reticulum	965
GO:0005773	vacuole	884
GO:0005618	cell wall	863
GO:0005730	nucleolus	830
GO:0005768	endosome	729
GO:0005802	trans-Golgi network	717

Supplementary Table S18. Conserved genes between Mesoamerican (BAT93) and Andean (G19833) bean accessions. Number of genes mapped between the two available bean genomes BAT93 and G19833 depending on the method employed. Total number of mapped genes is lower than the sum of different categories due to minor overlaps caused by genes with identical sequences for the longest transcript.

**Method**		**BAT93 genes mapped to G19833**	**G19833 genes mapped to BAT93**
**Orthology**	Reciprocal One-to-One Orthology	20,301	20,298
	One-to-One detected only from BAT93	637	639
	One-to-One detected only from G19833	550	551
**Identical Sequences**		13	13
**Synteny**	Previous protein	27	28
	Posterior protein	33	33
	Previous & posterior proteins	42	51
**Total**		21,600	21,604
**Placed on the linkage groups/chromosomes**		20,617	20,618

Supplementary Table S19. BAT93 and G19833 divergent PCGs at different identity levels. Cumulative PCG counts at different sequence identity levels before and after removing gaps from the orthologous gene alignments.

Sequence Identity threshold	Full alignments		Alignments without gaps	
	**BAT93 PCGs**	**G19833 PCGs**	**BAT93 PCGs**	**G19833 PCGs**
0.75 ≤	1,909	1,908	129	129
0.80 ≤	2,297	2,293	211	212
0.85 ≤	2,817	2,814	338	339
0.90 ≤	3,538	3,536	566	567
0.95 ≤	4,693	4,690	1,186	1,187

Supplementary Table S20. Functional enrichment for divergent BAT93 and G19833 PCGs at different identity levels. GO terms enriched for the divergent gene sets detected at different identity levels as shown in Supplementary Table S19.

	Sequence Identity Threshold	Ontology	GO Term	Name
***P. vulgaris* BAT93**				
**Full alignments including gaps**	0.75 ≤	Biological Process	GO:0006952	defense response
		Molecular Function	GO:0010333	terpene synthase activity
		Molecular Function	GO:0043531	ADP binding
	0.80 ≤	Biological Process	GO:0006952	defense response
		Molecular Function	GO:0010333	terpene synthase activity
		Molecular Function	GO:0030247	polysaccharide binding
		Molecular Function	GO:0043531	ADP binding
	0.85 ≤	Biological Process	GO:0006952	defense response
		Molecular Function	GO:0010333	terpene synthase activity
		Molecular Function	GO:0043531	ADP binding
	0.90 ≤	Molecular Function	GO:0043531	ADP binding
	0.95 ≤	Molecular Function	GO:0030247	polysaccharide binding
		Molecular Function	GO:0043531	ADP binding
**Alignments without gaps**	0.85 ≤	Molecular Function	GO:0043531	ADP binding
	0.90 ≤	Biological Process	GO:0006952	defense response
		Molecular Function	GO:0043531	ADP binding
	0.95 ≤	Biological Process	GO:0006952	defense response
		Molecular Function	GO:0030247	polysaccharide binding
		Molecular Function	GO:0043531	ADP binding
***P. vulgaris* G19833**				
**Full alignments including gaps**	0.75 ≤	Biological Process	GO:0006952	defense response
		Molecular Function	GO:0010333	terpene synthase activity
		Molecular Function	GO:0043531	ADP binding
	0.80 ≤	Biological Process	GO:0006952	defense response

		Molecular Function	GO:0010333	terpene synthase activity
		Molecular Function	GO:0043531	ADP binding
	0.85 ≤	Biological Process	GO:0006952	defense response
		Molecular Function	GO:0043531	ADP binding
	0.90 ≤	Biological Process	GO:0006952	defense response
		Molecular Function	GO:0043531	ADP binding
**Alignments without gaps**	0.80 ≤	Molecular Function	GO:0043531	ADP binding
	0.85 ≤	Biological Process	GO:0006952	defense response
		Molecular Function	GO:0043531	ADP binding
	0.90 ≤	Biological Process	GO:0006952	defense response
		Molecular Function	GO:0043531	ADP binding
	0.95 ≤	Biological Process	GO:0006952	defense response
		Molecular Function	GO:0030247	polysaccharide binding

Supplementary Table S21. Disease resistance genes. A. Disease resistance genes in P. vulgaris **B.** Disease resistance genes in other plants; data obtained from PRGDB [44] and from [45, 81, 82]

A.

R-gene type	Class	Number of proteins	Number of genes
**Cytoplasmic classes**			
CC-NBS-LRR	CNL	20	15
TIR-NBS-LRR	TNL	38	14
NBS-LRR	NL	83	23
RPW8-NBS-LRR	RPW8-NL	4	2
CC-NBS	CN	68	13
NBS	N	131	33
TIR	T	71	14
TIR-NBS	TN	0	0
With predicted NB-ARC domain		120	120
**Transmembrane classes**			
KIN-SerTHR	RLK	1458	434
KIN-GNK2	RLK-GNK2	8	6
KIN-LRR	RLP	407	153
**Other**			
MLO-like		27	10
PTO-like		18	15
**Total**		**2453**	**852**

B.

**R-gene class**	P. vulgaris, BAT93	P. vulgaris, G19833	G. max	C. arietinum	M. truncatula	A. thaliana	V. vinifera	C. melo
**CNL**	15	104	29	27	124	40	60	21
**TNL**	14	93	175	15	298	97	19	21
**NL**	23	155	52	1	147	11	111	10
**RPW8-NL**	2	NA	4	NA	2	6	10	3
**CN**	13	4	143	26	129	2	74	11
**N**	33	7	15	4	111	4	18	4
**T**	14	NA	12	6	10	38	7	6
**TN**	0	13	7	7	9	14	3	4

Supplementary Table S22. Number of small ncRNA in plants from Rfam database. For *P. vulgaris* we show the number of *de novo* predictions before and after validation with RNA-Seq ("filtered" column).

	A. thaliana	***G. max***	***M. truncatula***	***P. vulgaris***	***P. vulgaris* (filtered)**
**tRNA**	492	965	399	715	712
**rRNA sub**	77	311	91	149	145
**miRNA**	191	276	215	741	309
**snoRNA**	342	576	270	569	356
**snRNA**	59	171	127	195	165
**Nodulin**	1	8	1	2	2

Supplementary Table S23. LncRNA annotation statistics. Total number of predicted genes and transcripts and subsets formed by *ab initio* and homology predictions. In brackets the number of expressed lncRNAs (at least 0.1 RPKM in one of the 27 RNA-Seq libraries)..

	**Total**	***Ab initio***	**Homology**
**Transcripts**	1,858 (1,701)	1,801 (1,692)	59 (9)
**Genes**	1,033 (1,003)	995 (994)	38 (9)

Supplementary Table S24. Species used for the comparative genome analyses. Set of species used for comparative genome analyses. Columns include, in this order, scientific names, UniProt species code, the number of unique longest transcripts used in the analyses, the data source and the date in which data was retrieved. The species code for *P. vulgaris* G19833 is different from the species code registered in UniProt in order to differentiate the gene from the two bean accessions.

Species name	UniProt Species Code	Unique longest transcripts	Source	As in
Phaseolus vulgaris BAT93	**PHAVU**	30,405	PhasIbeAM - 10F	01/06/2014
Phaseolus vulgaris G19833	**PHAV1 ***	27,126	Phytozome v1.0	01/06/2014
Fragaria vesca	**FRAVE**	34,775	strawberrygenome.org	01/03/2011
Vitis vinifera	**VITVI**	29,894	Ensembl Plants - Release 15	01/09/2012
Theobroma cacao	**THECC**	28,839	Plaza - v2.5	01/09/2012
Cucumis melo	**CUCME**	27,376	melonomics.upv.es	01/04/2011
Populus trichocarpa	**POPTR**	41,186	Ensembl Plants - Release 15	01/09/2012
Arabidopsis thaliana	**ARATH**	27,233	Ensembl Plants - Release 17	01/04/2013
Prunus persica	**PRUVE**	27,950	PlantgDB	01/04/2013
Cajanus cajan	**CAJCA**	47,485	GigaDB	01/04/2013
Cicer arietinum	**CICAR**	27,494	ICRISAT	01/04/2013
Glycine max	**SOYBN**	53,821	Ensembl Plants - Release 17	01/04/2013
Medicago truncatula	**MEDTR**	43,205	Plaza - v2.5	01/09/2012
Solanum lycopersicum	**SOLLC**	34,635	International Tomato Annotation Group	01/02/2012

Supplementary Table S25. *P. vulgaris* orthologs. Detected orthologs between a given species and *P. vulgaris* BAT93. First column indicates how many trees have been used to detect such orthologs. Columns *unique* refers to the number of orthologs detected for each pair of species after removing redundancy. In one-to-many and many-to-many orthology relationships is possible to count more than once a given protein. Regarding to the ratios values, *all* column refers to the orthology ratio computed using all orthologous pairs meanwhile *unique* refers to the ratio computed using *unique* columns.

**Trees used for predict orthologs**	***Phaseolus vulgaris BAT93***		**Plant species**			**ratios**	
	**orthologs**	**unique**		**orthologs**	**unique**	**all**	**unique**
17,601	36,606	18,384	CICAR	22,600	17,567	1.62	1.05
18,365	34,746	19,832	THECC	22,512	14,820	1.54	1.34
20,880	57,344	22,039	CAJCA	37,408	22,097	1.53	1.00
16,105	29,479	17,549	CUCME	19,393	13,338	1.52	1.32
17,502	33,703	19,119	VITVI	23,390	15,167	1.44	1.26
13,612	29,871	15,243	MEDTR	20,755	15,470	1.44	0.99
17,827	32,801	19,361	FRAVE	23,237	15,127	1.41	1.28
18,312	35,559	19,766	PRUVE	25,836	16,558	1.38	1.19
17,411	34,576	19,465	SOLLC	26,161	16,956	1.32	1.15
15,790	27,302	17,355	ARATH	23,983	16,512	1.14	1.05
17,991	34,851	19,659	POPTR	34,604	22,433	1.01	0.88
22,101	37,580	22,994	SOYBN	41,890	36,217	0.90	0.63

Supplementary Table S26. ***P. vulgaris*** **orthologs.** Orthology ratios for a given species related to *P. vulgaris* BAT93. On this case, orthology relationships with 10 or more proteins for any of the species are discarded in order to avoid biases introduced by species-specific gene families expansions.

**Trees used for predict orthologs**	***Phaseolus vulgaris BAT93***		**Plant species**			**ratios**	
	**orthologs**	**unique**		**orthologs**	**unique**	**all**	**unique**
17,243	21,070	17,609	CICAR	19,255	16,797	1.09	1.05
18,139	30,744	19,293	THECC	20,825	14,581	1.48	1.32
20,130	24,002	20,653	CAJCA	22,490	20,023	1.07	1.03
15,968	27,174	17,028	CUCME	18,889	13,227	1.44	1.29
17,220	29,367	18,414	VITVI	20,989	14,535	1.40	1.27
13,166	16,665	13,598	MEDTR	17,570	14,749	0.95	0.92
17,616	29,728	18,708	FRAVE	21,822	14,683	1.36	1.27
17,980	30,550	19,034	PRUVE	22,975	15,784	1.33	1.21
17,159	30,786	18,567	SOLLC	24,538	16,590	1.25	1.12
15,660	25,873	16,870	ARATH	22,696	16,079	1.14	1.05
17,642	29,610	18,767	POPTR	30,681	21,655	0.97	0.87
21,700	25,354	21,962	SOYBN	39,215	35,824	0.65	0.61

Supplementary Table S27. GO terms for legume genes. Functional enriched GO Terms for BAT93 proteins found either in all legumes species used in this project or at least in one of them. Of note that proteins with homologs in at least one legume species is any of the studied one but not *P. vulgaris*.

**Homology profile**	**Go Term**	**Ontology**	**Level**	**Name**
All legumes species	GO:0045735	Molecular function	1	Nutrient reservoir activity
All legumes species	GO:0009405	Biological process	2	Pathogenesis
At least one legume species	GO:0003676	Molecular function	3	Nucleic acid binding
At least one legume species	GO:0003964	Molecular function	6	RNA-directed DNA polymerase activity
At least one legume species	GO:0004523	Molecular function	8	RNA-DNA hybrid ribonuclease activity
At least one legume species	GO:0008270	Molecular function	6	Zinc ion binding
At least one legume species	GO:0016301	Molecular function	4	Kinase activity
At least one legume species	GO:0045735	Molecular function	1	Nutrient reservoir activity
At least one legume species	GO:0006259	Biological process	6	DNA metabolic process
At least one legume species	GO:0006278	Biological process	8	RNA-dependent DNA replication
At least one legume species	GO:0009405	Biological process	2	Pathogenesis
At least one legume species	GO:0009987	Biological process	1	Cellular process
At least one legume species	GO:0015074	Biological process	7	DNA integration
At least one legume species	GO:0016310	Biological process	5	Phosphorylation
At least one legume species	GO:0044260	Biological process	4	Cellular macromolecule metabolic process
At least one legume species	GO:0090304	Biological process	5	Nucleic acid metabolic process
At least one legume species	GO:0090305	Biological process	6	Nucleic acid phosphodiester bond hydrolysis
At least one legume species	GO:0090501	Biological process	7	RNA phosphodiester bond hydrolysis
At least one legume species	GO:0090502	Biological process	8	RNA phosphodiester bond hydrolysis, endonucleolytic

Supplementary Table S28. Ratio of duplication events at the four relative evolutionary ages. Columns include also the number of duplication events detected for each age, the number of trees with such events, the number of genes involved in such duplication events, and the total number of trees present in the BAT93 phylome. In this analysis, only the *P. vulgaris* BAT93 accession was used.

Relative Age	Duplication events detected	Trees with duplication events	Genes involved in duplications	Trees in the BAT93 phylome	ratio
Basal to P. vulgaris	16,124	7,413	8,366	27,986	0.5761
Basal to legumes	12,417	10,001	11,993		0.4437
Basal to rosids	3,023	2,556	4,764		0.1080
Basal to rosids + asterids	50,677	14,966	16,912		1.8108

Supplementary Table S29. Ratio of duplication events at the four relative evolutionary ages after removing *P. vulgaris* specific-expansions of more than 10 members. Such filtering has been introduced to avoid any biases regarding the % of genes duplicated at each relative age. Columns include also the number of duplication events detected for each age, the number of trees with such events, the number of genes involved in such duplication events, and the total number of trees present in the BAT93 phylome. In this analysis, only the *P. vulgaris* BAT93 accession was used.

Relative Age	Duplication events detected	Trees with duplication events	Genes involved in duplications	Trees in the BAT93 phylome	ratio
Basal to P. vulgaris	5,265	3,887	5,820	24,460	0.2152
Basal to legumes	11,942	9,748	11,048		0.4882
Basal to rosids	2,983	2,524	4,489		0.1220
Basal to rosids + asterids	50,644	14,942	16,742		2.0705

Supplementary Table S30. Expression of protein coding genes (PCGs) and long non-coding RNAs (lncRNAs). **A. Number of expressed genes.** Genes were considered to be expressed at a threshold ≥0.1 RPKMs (or ≥1 RPKMs). The number of the expressed genes was calculated either individually by samples – we calculated number of genes expressed in at least one sample (column "Any"), or simultaneously in all samples (column "All"); or by organs – column "Any" contains number of genes expressed in at least one organ and "All" those genes expressed in all organs. **B. Organ-specific genes.** Gene considered to be organ-specific with average expression greater or equal to 0.1 RPKMs in a given organ and smaller in the rest of organs. **C. Stage-specific genes.** Gene considered to be stage-specific with average expression greater or equal to 0.1 RPKMs in a given stage and smaller in the rest.

A.

	**Total**	**Expression by samples**				**Expression by organs**			
		**Any**		**All**		**Any**		**All**	
		**0.1**	**1**	**0.1**	**1**	**0.1**	**1**	**0.1**	**1**
**PCGs**	30,491	23,198 (76%)	21,472 (70.4%)	14,305 (47%)	9,881 (32.4%)	22,714 (74.5%)	20,525 (67.3%)	17,299 (56.7%)	12,261 (40.2%)
**lncRNAs**	1,003	1,001 (99.8%)	684 (68.2%)	212 (21%)	75 (7.5%)	915 (91.2%)	521 (51.9%)	281 (28%)	99 (9.87%)

B.

**Organ**	**PCGs**	**lncRNAs**
**Axial meristem**	185	22
**Flower**	172	5
**Leaf**	59	17
**Pod**	85	43
**Root**	306	36
**Seed**	70	41
**Stem**	60	7
**Total**	**937**	**171**

C.

Stage	PCGs	lncRNAs
**V0**	74	20
**V1**	44	22
**V2**	30	4
**V3**	36	21
**V4**	56	6
**R5**	120	12
**R6**	169	4
**R7**	50	16
**R8**	23	12
**R9**	22	10
**Total**	**624**	**130**

Supplementary Table S31. Proportion of genes in the co-expression network at different relative evolutionary ages. The proportions were computed with respect to all genes used to construct the network. A fisher exact test was used to detect over/under-represented groups of genes at a given evolutionary age.

Relative Age	Genes per age	proportion (n = 30,404)	Genes per age – network	proportion (n = 8,638)	Fisher exact test (p-value)
Basal to P. vulgaris	5,699	0.1878	178	0.0206	-6.124e-12
Basal to legumes	2,400	0.0791	510	0.0590	2.325e-10
Basal to rosids	4,627	0.1524	1,483	0.1717	1.28e-05
Basal to rosids + asterids	17,627	0.5807	6,467	0.7487	4.048e-11

Supplementary Table S32. Proportion of genes in the largest modules from the co-expression network at different relative evolutionary ages. Proportion of genes from the 11 modules in the co-expression network with 100 or more connections with respect to all genes used to build the network. Proportions are given at different relative evolutionary ages. Statistical significance of under/over-representation of a genes assigned to a given age as compared to the whole genome was computed using a Fisher's exact test. Of note only protein-coding genes were taken into consideration since the evolutionary dating was only available for this type of sequences. Protein-coding genes represent more than 97% of the total genes present in the network.

**Whole genome proportions**		**Phaseolus-specific**			**Legumes**		
Number of protein-coding genes		Number of protein-coding genes		proportion	Number of protein-coding genes		proportion
30,353		5,699		0.1874	2,400		0.0789
**Modules**		**Module proportions**					
**ID**	Number of protein coding genes	**Phaseolus-specific**			**Legumes**		
		Number of protein-coding genes	proportion	Fisher's exact test	Number of protein-coding genes	proportion	Fisher's exact test
1	1,263	15	0.0119	2.4840E-87	38	0.0301	1.0790E-12
2	1,132	13	0.0115	3.5380E-79	48	0.0424	1.3850E-06
3	770	15	0.0195	1.6550E-47	87	0.1130	1.1830E-03
4	708	23	0.0325	2.2590E-35	46	0.0650	0.18000
5	600	5	0.0083	1.7220E-45	19	0.0317	3.2320E-06
6	459	6	0.0131	3.2180E-32	8	0.0174	1.8590E-08
7	414	5	0.0121	1.1940E-29	10	0.0242	4.7060E-06
8	326	15	0.0460	9.3070E-14	42	0.1288	1.9360E-03
9	198	1	0.0051	1.3540E-16	11	0.0556	0.28790
10	188	5	0.0266	2.6530E-11	22	0.1170	0.07674
11	146	3	0.0205	7.7070E-10	14	0.0959	0.44020
**Whole genome proportions**		**Rosids**			**Rosids+Asterids**		
Number of protein-coding genes		Number of protein-coding genes		proportion	Number of protein-coding genes		proportion
30,353		4,627		0.1522	17,627		0.5798
**Modules**		**Module proportions**					
**ID**	Number of protein coding genes	**Rosids**			**Rosids+Asterids**		
		Number of protein-coding genes	proportion	Fisher's exact test	Number of protein-coding genes	proportion	Fisher's exact test
1	1,263	118	0.0934	1.4210E-09	1,092	0.8646	4.1960E-103
2	1,132	146	0.1290	0.03130	925	0.8171	3.3130E-62
3	770	204	0.2649	2.7750E-15	464	0.6026	0.2230
4	708	166	0.2345	1.5100E-08	473	0.6681	2.5180E-06
5	600	49	0.0817	3.4830E-07	527	0.8783	1.5410E-56
6	459	55	0.1198	0.05713	390	0.8497	3.7110E-35
7	414	47	0.1135	0.02734	352	0.8502	5.0240E-32

8	326	94	0.2883	6.0420E-10	175	0.5368	0.11470
9	198	55	0.2778	6.2490E-06	131	0.6616	0.02071
10	188	45	0.2394	2.1630E-03	116	0.6170	0.37530
11	146	25	0.1712	0.48990	104	0.7123	1.3200E-03

SUPPLEMENTARY DATASETS

Dataset S1. BAT93/Jalo EEP558 F5 samples used for GBS analysis. In green – samples used for initial genotyping and misassembly correction.

Dataset S2. GO terms enrichment for the strain-specific expanded gene families in the BAT93 genome.

Dataset S3. GO terms enrichment for the lineage-specific expanded gene families of P. vulgaris.

Dataset S4. GO terms enrichment for proteins duplicated at the four relative ages proposed in this study.

Dataset S5. Mapping of RNA-seq reads to the reference assembly BAT93 v.10

Dataset S6. Protein coding and long non-coding RNA genes expression values, read counts

Dataset S7. Protein coding and long non-coding RNA genes expression values, RPKM normalization

Dataset S8. Organ-specific, stage-specific and housekeeping gene lists

Dataset S9. Housekeeping genes GO terms enrichment

Dataset S10. Organ-specific genes GO terms enrichment

Dataset S11. Stage-specific genes GO terms enrichment

Dataset S12. Number of differentially expressed protein-coding and long non-coding genes in pairwise samples comparison. In the columns numbers reflect UP regulated genes in a given conditions and in the rows – number of DOWN regulated genes

Dataset S13. Number of differentially expressed protein-coding and long non-coding genes in pairwise organ comparison. In the columns numbers reflect UP regulated genes in a given conditions and in the rows – number of DOWN regulated genes

Dataset S14. Number of differentially expressed protein-coding and long non-coding genes in pairwise stages comparison. In the columns numbers reflect UP regulated genes in a given conditions and in the rows – number of DOWN regulated genes

Dataset S15. Up and Down regulated genes in the Root development

Dataset S16. GO terms enrichment in the Root development Definition of up or down regulated genes are always in the respect to the first sample. Here are the categories related to the Biological Process only.

Dataset S17. Up and Down regulated genes in the Pod/Seed development

Dataset S18. GO terms enrichment in the Pod/Seed development. Definition of up or down regulated genes are always in the respect to the first sample. Here are the categories related to the Biological Process only.

Dataset S19. Up and Down regulated genes in the Leaf development

Dataset S20. GO terms enrichment in the Leaf development. Definition of up or down regulated genes are always in the respect to the first sample. Here are the categories related to the Biological Process only.

Dataset S21. Up and Down regulated genes in the Stem development

Dataset S22. GO terms enrichment in the Stem development. Definition of up or down regulated genes are always in the respect to the first sample. Here are the categories related to the Biological Process only.

Dataset S23. Co-expression modules with >100 genes

Dataset S24. GO terms enrichment for the co-expression modules with >100 genes

SUPPLEMENTARY REFERENCES

1. Kelly JD, Gepts P, Miklas PN, Coyne DP. **Tagging and mapping of genes and QTL and molecular marker-assisted selection for traits of economic importance in bean and cowpea**. *Field Crops Research* 2003; 135–154.

2. Sánchez Valdez I, Ibarra Pérez FJ, Rosales Serna R, Singh SP, Acosta Gallegos JA. **Pinto Saltillo: new bean variety for the Highlands of Mexico.** *Agric Técnica en México* 2001; **27**:73–75.

3. Freyre R, Skroch PW, Geffroy V, Adam-Blondon AF, Shirmohamadali A, Johnson WC,et al. **Towards an integrated linkage map of common bean. 4. Development of a core linkage map and alignment of RFLP maps**. *Theor Appl Genet* 1998, **97**:847–856.

4. Grisi MCM, Blair MW, Gepts P, Brondani C, Pereira PAA, Brondani RP V. **Genetic mapping of a new set of microsatellite markers in a reference common bean (Phaseolus vulgaris) population BAT93 x Jalo EEP558**. *Genet Mol Res* 2007, **6**:691–706.

5. Fernandez F, Gepts P. & Lopes M. **Etapas de desarrollo de la planta de frijol (Phaseolus vulgarisL.).** Cali, Centro Internacional de Agricultura Tropical, 1986. 34p.

6. Kami J, Poncet V, Geffroy V, Gepts P. **Development of four phylogenetically-arrayed BAC libraries and sequence of the APA locus in Phaseolus vulgaris**. *Theor Appl Genet* 2006, **112**:987–998.

7. Kim H, San Miguel P, Nelson W, Collura K, Wissotski M, Walling JG,et al. **Comparative physical mapping between Oryza sativa (AA genome type) and O. punctata (BB genome type)**. *Genetics* 2007, **176**:379–390.

8. Minoche AE, Dohm JC, Himmelbauer H. **Evaluation of genomic high-throughput sequencing data generated on Illumina HiSeq and Genome Analyzer systems**. *Genome Biology* 2011:R112.

9. Dohm JC, Minoche AE, Holtgräwe D, Capella-Gutiérrez S, Zakrzewski F, Tafer H, et al. **The genome of the recently domesticated crop plant sugar beet (Beta vulgaris).** *Nature* 2014, **505**:546–9.

10. Li H, Durbin R. **Fast and accurate long-read alignment with Burrows-Wheeler transform**. *Bioinformatics* 2010, **26**:589–595.

11. Li H, Handsaker B, Wysoker A, Fennell T, Ruan J, Homer N, Marth G, et al. **The Sequence Alignment / Map (SAM) Format and SAMtools 1000 Genome Project Data Processing Subgroup**. *Bioinformatics* 2009, **25**:1–2.

12. Luo R, Liu B, Xie Y, Li Z, Huang W, Yuan J, et al. **SOAPdenovo2: an empirically improved memory-efficient short-read de novo assembler.** *Gigascience* 2012, **1**:18.

13. McKenna A, Hanna M, Banks E, Sivachenko A, Cibulskis K, Kernytsky A, et al. **The genome analysis toolkit: A MapReduce framework for analyzing next-generation DNA sequencing data**. *Genome Res* 2010, **20**:1297–1303.

14. Boetzer M, Henkel C V., Jansen HJ, Butler D, Pirovano W. **Scaffolding pre-assembled contigs using SSPACE**. *Bioinformatics* 2011, **27**:578–579.

15. Hiroyoshi I, Seishi N. **AntMap: Constructing Genetic Linkage Maps Using an Ant Colony Optimization Algorithm**. *Breed Sci Vol 56 , No 4 371-377* 2007.

16. Flutre T, Duprat E, Feuillet C, Quesneville H. **Considering transposable element diversification in de novo annotation approaches**. *PLoS One* 2011, **6**.

17. Wicker T, Sabot F, Hua-Van A, Bennetzen JL, Capy P, Chalhoub B,et al. **A unified classification system for eukaryotic transposable elements.** *Nat Rev Genet* 2007, **8**:973–982.

18. Ellinghaus D, Kurtz S, Willhoeft U. **LTRharvest, an efficient and flexible software for de novo detection of LTR retrotransposons.** *BMC Bioinformatics* 2008, **9**:18.

19. Steinbiss S, Willhoeft U, Gremme G, Kurtz S. **Fine-grained annotation and classification of de novo predicted LTR retrotransposons**. *Nucleic Acids Res* 2009, **37**:7002–7013.

20. **RepeatMasker Open-3.0**

21. Jurka J, Kapitonov V V., Pavlicek A, Klonowski P, Kohany O, Walichiewicz J. **Repbase Update, a database of eukaryotic repetitive elements**. *Cytogenet Genome Res* 2005, **110**:462–467.

22. Kent WJ. **BLAT - The BLAST-like alignment tool**. *Genome Res* 2002, **12**:656–664.

23. Altschul SF, Gish W, Miller W, Myers EW, Lipman DJ. **Basic local alignment search tool.** *J Mol Biol* 1990, **215**:403–410.

24. Marco-Sola S, Sammeth M, Guigó R, Ribeca P. **The GEM mapper: fast, accurate and versatile alignment by filtration**. *Nature Methods* 2012.

25. Trapnell C, Williams BA, Pertea G, Mortazavi A, Kwan G, van Baren MJ, et al. **Transcript assembly and abundance estimation from RNA-Seq reveals thousands of new transcripts and switching among isoforms**. *Nat Biotechnol* 2011, **28**:511–515.

26. Haas BJ, Salzberg SL, Zhu W, Pertea M, Allen JE, Orvis J, et al. **Automated eukaryotic gene structure annotation using EVidenceModeler and the Program to Assemble Spliced Alignments.** *Genome Biol* 2008, **9**:R7.

27. Blanco E, Parra G, Guigó R. **Using geneid to identify genes.** *Curr Protoc Bioinformatics* 2007, **Chapter 4**:Unit 4.3.

28. Parra G, Agarwal P, Abril JF, Wiehe T, Fickett JW, Guigo R. **Comparative gene prediction in human and mouse**. *Genome Res* 2003, **13**:108–17.

29. Stanke M, Keller O, Gunduz I, Hayes A, Waack S, Morgenstern B. **AUGUSTUS: A b initio prediction of alternative transcripts**. *Nucleic Acids Res* 2006, **34**(WEB. SERV. ISS.).

30. Majoros WH, Pertea M, Salzberg SL. **TigrScan and GlimmerHMM: Two open source ab initio eukaryotic gene-finders**. *Bioinformatics* 2004, **20**:2878–2879.

31. Finn RD, Bateman A, Clements J, Coggill P, Eberhardt RY, Eddy SR, et al. **Pfam: The protein families database**. *Nucleic Acids Research* 2014.

32. Hunter S, Jones P, Mitchell A, Apweiler R, Attwood TK, Bateman A, et al. **InterPro in 2011: New developments in the family and domain prediction database**. *Nucleic Acids Res* 2012, **40**.

33. Kanehisa M, Goto S, Sato Y, Furumichi M, Tanabe M. **KEGG for integration and interpretation of large-scale molecular data sets**. *Nucleic Acids Res* 2012, **40**.

34. Croft D, Mundo AF, Haw R, Milacic M, Weiser J, Wu G,et al.**The Reactome pathway knowledgebase**. *Nucleic Acids Res* 2014, **42**.

35. Huerta-Cepas J, Capella-Gutiérrez S, Pryszcz LP, Marcet-Houben M, Gabaldón T. **PhylomeDB v4: Zooming into the plurality of evolutionary histories of a genome**. *Nucleic Acids Res* 2014, **42**.

36. Götz S, García-Gómez JM, Terol J, Williams TD, Nagaraj SH, Nueda MJ, et al. **High-throughput functional annotation and data mining with the Blast2GO suite**. *Nucleic Acids Res* 2008, **36**:3420–3435.

37. Petersen TN, Brunak S, von Heijne G, Nielsen H. **SignalP 4.0: discriminating signal peptides from transmembrane regions**. *Nature Methods* 2011:785–786.

38. Jones P, Binns D, Chang HY, Fraser M, Li W, McAnulla C, et al. **InterProScan 5: Genome-scale protein function classification**. *Bioinformatics* 2014, **30**:1236–1240.

39. Nawrocki EP, Kolbe DL, Eddy SR. **Infernal 1.0: Inference of RNA alignments**. *Bioinformatics* 2009, **25**:1335–1337.

40. Griffiths-Jones S, Moxon S, Marshall M, Khanna A, Eddy SR, Bateman A. **Rfam: Annotating non-coding RNAs in complete genomes**. *Nucleic Acids Res* 2005, **33**(DATABASE ISS.).

41. Langmead B, Salzberg SL. **Fast gapped-read alignment with Bowtie 2**. *Nature Methods* 2012:357–359.

42. Anders S, Pyl PT, Huber W. *HTSeq A Python Framework to Work with High-Throughput Sequencing Data*. 2014.

43. Schmieder R, Lim YW, Edwards R. **Identification and removal of ribosomal RNA sequences from metatranscriptomes**. *Bioinformatics* 2012, **28**:433–435.

44. Sanseverino W, Hermoso A, D'Alessandro R, Vlasova A, Andolfo G, Frusciante L,et al. **PRGdb 2.0: Towards a community-based database model for the analysis of R-genes in plants**. *Nucleic Acids Res* 2013, **41**.

45. Schmutz J, McClean PE, Mamidi S, Wu GA, Cannon SB, Grimwood J, et al. **A reference genome for common bean and genome-wide analysis of dual domestications.** *Nat Genet* 2014, **46**:707–13.

46. Esteve-Codina A, Kofler R, Palmieri N, Bussotti G, Notredame C, Pérez-Enciso M. **Exploring the gonad transcriptome of two extreme male pigs with RNA-seq**. *BMC Genomics* 2011:552.

47. Jin J, Liu J, Wang H, Wong L, Chua NH. **PLncDB: Plant long non-coding RNA database**. *Bioinformatics* 2013:1068–1071.

48. Notredame C, Higgins DG, Heringa J. **T-Coffee: A novel method for fast and accurate multiple sequence alignment.** *J Mol Biol* 2000, **302**:205–217.

49. Kong L, Zhang Y, Ye ZQ, Liu XQ, Zhao SQ, Wei L, et al. **CPC: Assess the protein-coding potential of transcripts using sequence features and support vector machine**. *Nucleic Acids Res* 2007, **35**.

50. Mortazavi A, Williams BA, McCue K, Schaeffer L, Wold B. **Mapping and quantifying mammalian transcriptomes by RNA-Seq.** *Nat Methods* 2008, **5**:621–628.

51. Dobin A, Davis CA, Schlesinger F, Drenkow J, Zaleski C, Jha S, et al. **STAR: Ultrafast universal RNA-seq aligner**. *Bioinformatics* 2013, **29**:15–21.

52. Li B, Ruotti V, Stewart RM, Thomson JA, Dewey CN. **RNA-Seq gene expression estimation with read mapping uncertainty**. *Bioinformatics* 2009, **26**:493–500.

53. Severin AJ, Woody JL, Bolon Y-T, Joseph B, Diers BW, Farmer AD, et al. **RNA-Seq Atlas of Glycine max: a guide to the soybean transcriptome.** *BMC Plant Biol* 2010, **10**:160.

54. O'Rourke J, Iniguez LP, Fu F, Bucciarelli B, Miller SS, Jackson S, et al. **An RNA-Seq based gene expression atlas of the common bean**. *BMC Genomics* 2014, **15**:866.

55. McCarthy DJ, Chen Y, Smyth GK. **Differential expression analysis of multifactor RNA-Seq experiments with respect to biological variation**. *Nucleic Acids Res* 2012, **40**:4288–4297.

56. Lund SP, Nettleton D, McCarthy DJ, Smyth GK. **Detecting differential expression in RNA-sequence data using quasi-likelihood with shrunken dispersion estimates**. *Stat Appl Genet Mol Biol* 2012, **11**.

57. Friedman J, Hastie T, Tibshirani R. **Sparse inverse covariance estimation with the graphical lasso**. *Biostatistics* 2008, **9**:432–441.

58. Clauset A, Newman M, Moore C. **Finding community structure in very large networks**. *Physical Review E* 2004.

59. Huerta-cepas J, Dopazo J, Huynen M a., Gabaldón .: **Evidence for short-time divergence and long-time conservation of tissue-specific expression after gene duplication**. *Brief Bioinform* 2011, **12**:442–448.

60. Bellucci E, Bitocchi E, Ferrarini A, Benazzo A, Biagetti E, Klie S, et al. **Decreased Nucleotide and Expression Diversity and Modified Coexpression Patterns Characterize Domestication in the Common Bean.** *Plant Cell* 2014; **26**:1901–1912.

61. Kodama Y, Shumway M, Leinonen R; International Nucleotide Sequence Database Collaboration. **The Sequence Read Archive: explosive growth of sequencing data.** *Nucleic Acids Res.* 2012; **40(Database issue)**:D54-6

62. Smith TF, Waterman MS. **Identification of common molecular subsequences.** *J Mol Biol* 1981, **147**:195–197.

63. Edgar RC. **MUSCLE: a multiple sequence alignment method with reduced time and space complexity.** *BMC Bioinformatics* 2004, **5**:113.

64. Katoh K, Toh H. **Recent developments in the MAFFT multiple sequence alignment program**. *Brief Bioinform* 2008, **9**:286–298.

65. Lassmann T, Frings O, Sonnhammer ELL. **Kalign2: High-performance multiple alignment of protein and nucleotide sequences allowing external features**. *Nucleic Acids Res* 2009, **37**:858–865.

66. Landan G, Graur D. **Heads or tails: A simple reliability check for multiple sequence alignments**. *Mol Biol Evol* 2007, **24**:1380–1383.

67. Wallace IM, O'Sullivan O, Higgins DG, Notredame C. **M-Coffee: Combining multiple sequence alignment methods with T-Coffee**. *Nucleic Acids Res* 2006, **34**:1692–1699.

68. Capella-Gutiérrez S, Silla-Martínez JM, Gabaldón T. **trimAl: A tool for automated alignment trimming in large-scale phylogenetic analyses**. *Bioinformatics* 2009, **25**:1972–1973.

69. Gascuel O. **BIONJ: an improved version of the NJ algorithm based on a simple model of sequence data**. *Mol Biol Evol* 1997, **14**:685–695.

70. Guindon S, Dufayard JF, Lefort V, Anisimova M, Hordijk W, Gascuel O. **New algorithms and methods to estimate maximum-likelihood phylogenies: Assessing the performance of PhyML 3.0**. *Syst Biol* 2010, **59**:307–321.

71. Akaike H. **A new look at the statistical model identification**. *IEEE Trans Automat Contr* 1974, **19**.

72. Gabaldón T. **Large-scale assignment of orthology: back to phylogenetics?** *Genome Biol* 2008, **9**:235.

73. Huerta-Cepas J, Dopazo J, Gabaldón T. **ETE: a python Environment for Tree Exploration.** *BMC Bioinformatics* 2010, **11**:24.

74. Medina I, Carbonell J, Pulido L, Madeira SC, Goetz S, Conesa A, et al. **Babelomics: An integrative platform for the analysis of transcriptomics, proteomics and genomic data with advanced functional profiling**. *Nucleic Acids Res* 2010, **38**(SUPPL. 2).

75. Supek F, Bošnjak M, Škunca N, Šmuc T. **Revigo summarizes and visualizes long lists of gene ontology terms**. *PLoS One* 2011, **6**.

76. Huerta-Cepas J, Gabaldón T. **Assigning duplication events to relative temporal scales in genome-wide studies**. *Bioinformatics* 2011, **27**:38–45.

77. Yang Y, Moore MJ, Brockington SF, Soltis DE, Wong GK. **Dissecting molecular evolution in the highly diverse plant clade Caryophyllales using transcriptome sequencing**. 2015.

78. Thorvaldsdóttir H, Robinson JT, Mesirov JP. **Integrative Genomics Viewer (IGV): High-performance genomics data visualization and exploration**. *Brief Bioinform* 2013, **14**:178–192.

79. Zhulidov PA, Bogdanova EA, Shcheglov AS, Vagner LL, Khaspekov GL, Kozhemyako Vbet al. **Simple cDNA normalization using kamchatka crab duplex-specific nuclease.** *Nucleic Acids Res* 2004, **32**:e37.

80. Shagin DA, Rebrikov D V., Kozhemyako VB, Altshuler IM, Shcheglov AS, Zhulidov PA,et al. **A novel method for SNP detection using a new duplex-specific nuclease from crab hepatopancreas**. *Genome Research* 2002:1935–1942.

81. Varshney RK, Song C, Saxena RK, Azam S, Yu S, Sharpe G, et al. **Draft genome sequence of chickpea (Cicer arietinum) provides a resource for trait improvement**. *Nat Biotechnol* 2013, **31**(January):240–246.

82. Schmutz J, Cannon SB, Schlueter J, Ma J, Mitros T, Nelson W, et al. **Genome sequence of the palaeopolyploid soybean.** *Nature* 2010, **463**:178–183.
